# Newly developed monoclonal antibodies for marmoset immunosuppression enable KSHV lytic replication and salivary shedding: A promising model for KSHV pathogenesis studies

**DOI:** 10.1371/journal.ppat.1014698

**Published:** 2026-10-26

**Authors:** Li Han, Esther Rodriguez, Caitlin Shaw, Joe Kurian, Saverio Capuano, Gabriela M. Escalante, Murali Muniraju, Kyle Jung, Lorraine Z. Mutsvunguma, Kathleen Engelman, Jae Jung, Corinna N. Ross, Ivana G. Reidel, Javier Gordon Ogembo

**Affiliations:** 1 Department of Immuno-Oncology, Beckman Research Institute of City of Hope, Duarte, California, United States of America; 2 Irell & Manella Graduate School of Biological Sciences of City of Hope, Duarte, California, United States of America; 3 Wisconsin National Primate Research Center, University of Wisconsin-Madison, Madison, Wisconsin, United States of America; 4 Department of Microbial Sciences in Health, and Global Center for Pathogen and Human Health Research, Cleveland Clinic Research, Cleveland, Ohio, United States of America; 5 MassBiologics, UMass Chan Medical School, Boston, Massachusetts, United States of America; 6 Southwest National Primate Research Center, Texas Biomedical Research Institute, San Antonio, Texas, United States of America; University of Wisconsin-Madison, UNITED STATES OF AMERICA

## Abstract

Kaposi sarcoma-associated herpesvirus (KSHV) is the causative agent of Kaposi sarcoma, a malignancy that primarily affects immunocompromised individuals, and is also linked to multiple other diseases. Progress in understanding KSHV pathogenesis and developing effective vaccines or therapies has been severely limited by the lack of a reliable *in vivo* model that recapitulates infection, persistence, and disease. Previous work suggested that the common marmoset (*Callithrix jacchus*), a small New World primate, can support KSHV infection and pathogenesis, but the reproducibility and utility of this model has not been rigorously established. Here, we sought to validate the common marmoset as a non-human primate model for KSHV studies by evaluating viral infection under both immunocompetent and immunosuppressed conditions. We first tested the use of intravenous and oral tacrolimus-based immunosuppression, confirming infection in challenged marmosets but also encountering tacrolimus dosing inconsistencies and toxicity. To overcome these limitations, we developed and validated a targeted immunosuppression approach using two novel mouse–marmoset chimeric monoclonal antibodies, anti-CD4 [TAWA] and anti-CD8 [OCHO], to selectively deplete T cell subsets. Altogether, we demonstrated across four independent experiments that KSHV can establish systemic infection in both immunocompetent and immunosuppressed conditions marmosets, evidenced by viremia in peripheral blood mononuclear cells, detectable seroconversion, and tissue persistence. The chimeric antibodies mediated efficient, subset‑specific depletion of CD4^+^ and CD8^+^ T cells and enabled a quantifiable, well‑tolerated immunosuppressive regimen. Moreover, we showed for the first time that monoclonal antibody-treated marmosets exhibit KSHV shedding in saliva and lytic replication, indicating that infection in this model is not merely latent but supports a full, human‑like replication cycle. Together these findings validate the common marmoset as a susceptible and translationally relevant non-human primate for studying KSHV infection and introduce a practical immunosuppressive tool for dissecting viral pathogenesis and immunity *in vivo*, providing a platform for testing vaccine and therapeutics.

## Author summary

Kaposi sarcoma-associated herpesvirus (KSHV) causes Kaposi sarcoma, a cancer that affects the skin and internal organs, and is associated with several other diseases that primarily occur in people with weakened immune systems. Studying KSHV has been difficult because the virus naturally infects humans, and suitable animal models are limited. Prior studies showed that common marmosets, a small type of monkey, can be experimentally infected with KSHV, but this animal model has remained underdeveloped. To improve this model, we carefully characterized KSHV infection in marmosets and generated new antibody-based tools to temporarily weaken (i.e., immunosuppress) their immune systems. Using these tools, we demonstrated that KSHV establishes long-term infection in both healthy and immunosuppressed marmosets. Importantly, we showed for the first time that immunosuppressed marmosets can shed KSHV virus in their saliva, indicating KSHV is actively replicating, similar to what is seen in infected humans. Our findings validate the marmoset as a relevant model for KSHV infection and provide a valuable platform in which to study viral biology and to evaluate vaccines and potential treatments.

## Introduction

Kaposi sarcoma-associated herpesvirus (KSHV), also known as human herpesvirus 8 (HHV-8), is a key etiological agent linked to several malignancies, including primary effusion lymphoma, multicentric Castleman disease, and the endothelial cancer Kaposi sarcoma (KS) [1–4]. KSHV-associated malignancies disproportionately affect immunocompromised individuals [5–9]. This includes at-risk populations such as people >50 years old for classical KS in the Mediterranean and Eastern Europe regions [10], or <5 [11] years old for endemic KS; transplant patients taking immunosuppressive drugs [12]; or people infected with HIV [6,13-14]. Indeed, patients with HIV/AIDS have a 10,000‑fold higher relative risk of developing KS than HIV-negative individuals [13,15], making KS the most frequent cancer in patients with HIV/AIDS [16–18]. Despite substantial advances in understanding KSHV molecular biology and disease mechanisms, an absence of robust *in vivo* models continues to impede progress in elucidating viral pathogenesis and evaluating vaccine or therapeutic strategies [19].

Various humanized mouse models have been used to study systemic KSHV transmission and B cell- and T cell-mediated responses, but these models have several limitations: the mice require a high viral titer to be infected; they do not effectively model host responses to infection and are incapable of generating humoral responses; and they do not develop KS-like disease due to the inability of KSHV to infect murine endothelial cells *in vivo* [20–22]. Some groups have reported successful infection of humanized mice with KSHV through the oral route [21], but the reproducibility of this model remains to be confirmed. Additionally, an ideal KSHV animal model should not only recapitulate the viral life cycle but also enable the interrogation of the role of T cells in KSHV pathogenesis. T cells play a key role in controlling viral reactivation once latency is established [23,24], and their dysfunction markedly increases the risk of KS and other KSHV-associated diseases [23–25]. In light of these complex requirements, non-human primates (NHPs) have emerged as a promising option. NHPs have been instrumental in studying human diseases due to their close genetic and physiological resemblance to humans [26]. The rhesus macaque in particular has been extensively used to study infection with rhesus rhadinovirus, the rhesus KSHV-homologue herpesvirus, as a key surrogate KSHV challenge model [27,28]. In parallel, KSHV infection has also been studied in the common marmoset (*Callithrix jacchus*), a robust animal model that has emerged over the last years for biomedical research across various fields, including the modelling of human infectious diseases, because of its small size, ease of handling and breeding, and lower associated costs compared to other NHPs [29,30].

The first reported attempt to model KSHV infection and KS development in marmosets was successful in demonstrating the establishment of persistent KSHV infection in challenged marmosets [31]. In that early study, KSHV infectivity in marmosets was assessed after intravenous or oral challenge using rKSHV.219 recombinant virus, as well as after intravenous challenge in the presence of the immunosuppressive drug tacrolimus, to simulate the immunosuppression commonly seen in people receiving transplants or those living with HIV/AIDS. In marmosets, both intravenous and oral KSHV challenges resulted in the establishment of latent infection in multiple tissues, with one orally challenged marmoset also developing a KS-like lesion. Marmosets challenged intravenously in the presence of tacrolimus also became infected, but at much lower levels and in fewer types of tissues than marmosets challenged in the absence of tacrolimus. Tacrolimus induces immunosuppression by binding to the immunophilin FKBP12 and forming a complex that inhibits calcineurin, a critical enzyme in T cell activation pathways [32]; however, this process can also interfere with the KSHV life cycle [33]. It is possible, then, that the administration of tacrolimus before KSHV challenge interfered with the establishment of infection. Additionally, a sample size of n = 2 marmosets/group limited the statistical power of that study, restricting its ability to draw definitive conclusions about infection rates and disease progression. However, a subsequent study currently in preprint, in which immunocompetent marmosets were infected using a wildtype KSHV BAC16 virus (a bacterial artificial chromosome [BAC]-clone virus derived from rKSHV.219) and two KSHV BAC16 mutants, demonstrated that persistent KSHV infection can be established in healthy marmosets, reviving interest in this animal model [34]. Together, these studies provide a strong foundation and support for further development of common marmosets as a model for KSHV infection.

Building upon the previous studies [31,34], we aimed to confirm the reproducibility of KSHV challenge in the common marmoset and to develop novel immunosuppressive tools to support this model. We first conducted two experiments to examine how tacrolimus-mediated immunosuppression affects KSHV infection outcomes when administered *after* viral challenge, rather than before as tested by previous authors [31]. However, we soon identified key limitations: daily intravenous tacrolimus proved highly toxic, while its oral administration failed to achieve therapeutic blood levels in the marmosets. To overcome these limitations, we developed and characterized chimeric anti-CD4 [TAWA] and anti-CD8 [OCHO] monoclonal antibodies (mAbs), which incorporate mouse-derived variable regions and marmoset IgG constant regions. These mAbs effectively and safely depleted CD4^+^ and CD8^+^ T cell populations in marmosets over extended periods, eliminating the need for daily drug administration and avoiding the risks associated with long-term immunosuppressive therapy [35–38]. We then evaluated KSHV infection outcomes in marmosets treated intravenously with the chimeric mAbs under two different regimens, as well as in untreated control marmosets. In parallel, we validated a flow cytometry mAb panel compatible with the systemic presence of anti-CD4 [TAWA] and anti-CD8 [OCHO] mAbs, which enabled the tracking of major immune cell populations in marmosets, including T cells, B cells, monocytes, and natural killer (NK) cells. Collectively, our study introduces novel immunosuppressive tools that enhance the utility of the common marmoset as a preclinical model for KSHV infection. We demonstrate that KSHV infection can be reliably established in marmosets under both immunocompetent and immunosuppressed conditions in a larger cohort than that of prior studies. Importantly, we demonstrate for the first time that immunosuppressed marmosets can exhibit lytic viral replication detectable in saliva, highlighting the model’s ability to recapitulate key features of KSHV biology. These findings significantly advance the utility of the common marmoset model for dissecting KSHV pathogenesis and evaluating future candidate vaccines and antiviral therapies.

## Results

### Establishment of KSHV infection in common marmosets treated with intravenous or oral tacrolimus

To build upon the previously reported KSHV infection model [31], we challenged two common marmosets, Cj062 (female) and Cj074 (male), with two intravenous doses of rKSHV.219 (1 × 10⁷ iSLK infectious units [IU]) administered three months apart (day [D]0 and D88), at the Wisconsin National Primate Research Center (WNPRC). Beginning on D218, we administered the immunosuppressant tacrolimus as a daily intravenous dose, which continued through the end of the study on D304 (Fig 1A). To monitor KSHV viremia, we used quantitative PCR (qPCR) to measure copies of the KSHV gene LANA-1 in DNA extracted from peripheral blood mononuclear cells (PBMCs) isolated at various bleeding times (Fig 1B). In both marmosets, we detected low levels of PBMC viremia on D30 following the first viral challenge, but KSHV was undetectable by D45. A more pronounced PBMC viremia peak occurred approximately one month after the second viral challenge, on D112 in Cj074 and D127 in Cj062. By the study endpoint, we detected no PBMC viremia in either marmoset. As a secondary KSHV infection indicator, we also used enzyme-linked immunosorbent assay (ELISA) to assess KSHV seroconversion by measuring blood IgG levels against the KSHV lytic antigen viral capsid antigen (VCA), and the latent antigen LANA-1. Anti-KSHV VCA IgG levels followed similar kinetics to that of PBMC viremia, with observable peaks immediately after each challenge (Fig 1C), suggesting a strong correlation between the observable peaks in both techniques and the KSHV challenge timepoints. In contrast, only one animal displayed an anti-LANA-1 response, which peaked prior to the second challenge and remained elevated until tacrolimus administration, after which it plateaued at lower levels (Fig 1D). As an indicator of KSHV viral load at study endpoint, we used qPCR to quantify the LANA-1 gene in DNA extracted from marmoset tissues. Although the tacrolimus immunosuppression regimen did not produce clear evidence of viral replication (i.e., increased PBMC viremia or anti-KSHV VCA IgG after D218), detectable viral loads in both lymphoid (Fig 1E) and non-lymphoid (Fig 1F) tissues collected at necropsy (D304) confirmed long-term infection was established. Note that for this and all subsequent qPCR used to quantify viral load, we report LANA-1 copy numbers as copies per microgram of DNA when Ct values fell within the linear range of the standard curve in both technical replicates; if Ct values for one or both technical replicates fell outside of the linear range, we did not quantify copy numbers and instead display results as the change in cycle threshold (Ct), defined as the difference between the LANA-1 gene Ct and the β-actin housekeeping (HK) Ct (ΔCt = Ct_LANA-1_ – Ct_HK_) in a heatmap that displays ΔCt for all tested tissues in marmosets for all studies included in this manuscript (S10 Fig). In this initial tacrolimus experiment, we obtained quantifiable viral loads in the spleens and livers of both marmosets, and in the bone marrow, tonsil, thymus, and axillary lymph node tissues of one of the two marmosets. We also detected traces of KSHV DNA in other tissues such as mesenteric lymph nodes, lungs, tongue, and colon in at least one of the marmosets, as summarized in S10 Fig. Additionally, to further confirm splenic infection, we performed KSHV LANA-1 immunohistochemistry (IHC) and evaluated LANA-1 expression. In this and all subsequent KSHV challenge experiments, samples were classified using an arbitrary IHC scoring system based on three distinct staining criteria: a) color – cell must display the characteristic red-brown diaminobenzidine (DAB) staining signal; b) localization – the DAB signal must be clearly defined and restricted to the cell nucleus, as confirmed by counterstaining; and c) distribution – positive cells must be detectable in at least three independent fields of view at 10× magnification. Based on these criteria, samples were assigned one of four IHC scores (S11A Fig): IHC 0 (LANA-1 negative) – sample failed to meet color and localization criteria; IHC 1 (LANA-1 inconclusive) – sample met the color and localization criteria but fails to meet the distribution criterion; IHC 2 (LANA-1 positive) – sample met all the three criteria, with two or fewer LANA-1-positive cells observed per field; and IHC 3 (LANA-1 positive frequent) – sample met all three criteria, with more than two LANA-1-positive cells observed per field. In the initial tacrolimus-treated challenge experiment, LANA-1 expression was detected in the spleen of animal Cj062 (IHC 2, S12 Fig), consistent with the detection of anti-KSHV LANA-1 IgG responses. In contrast, LANA-1 staining in Cj074 was classified as inconclusive (IHC 1, S11A Fig).

**Fig 1:**
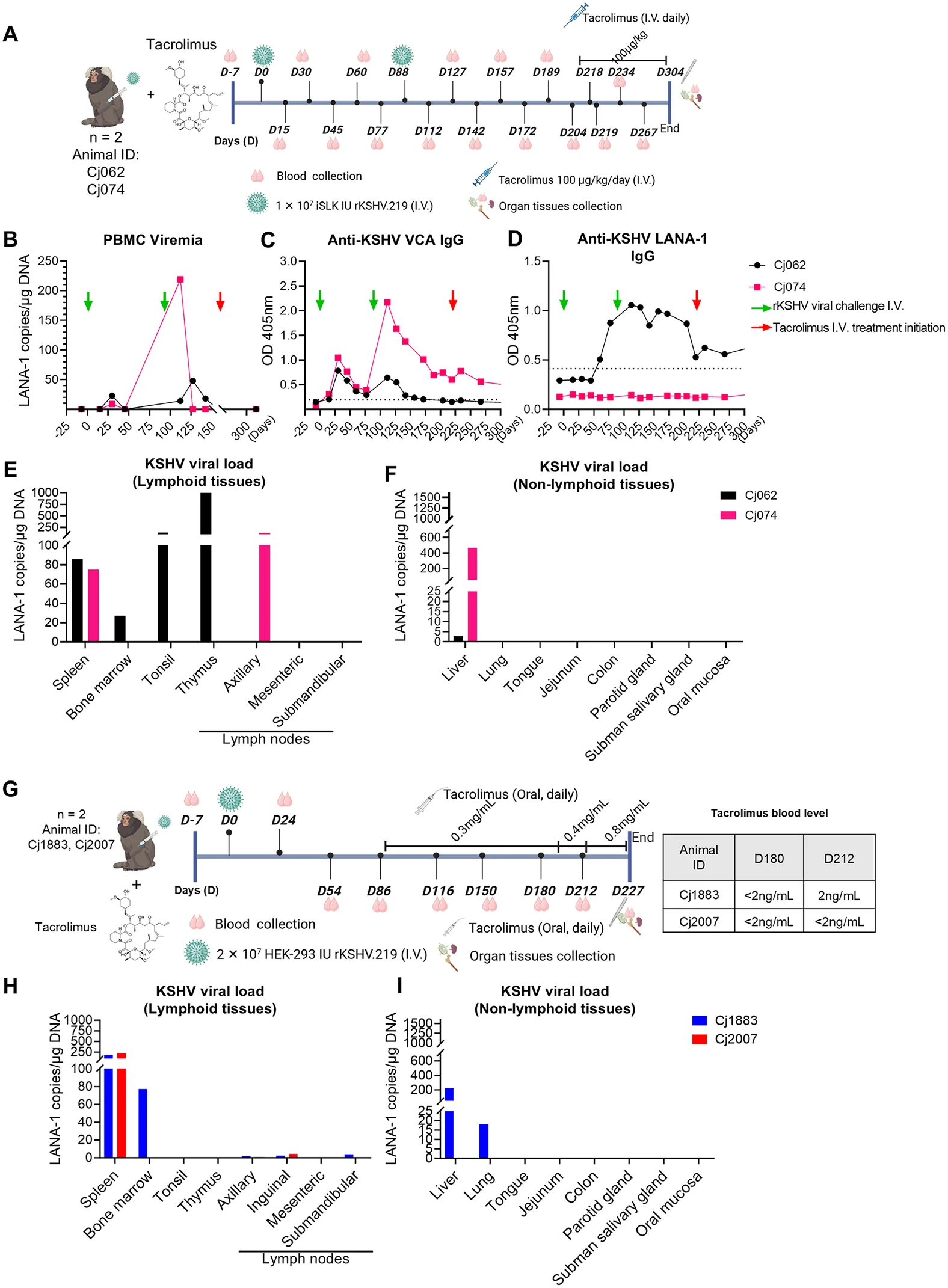
Intravenous (I.V.) rKSHV infection followed by I.V. or oral tacrolimus treatment in common marmosets. **(A)** Experimental marmoset treatment and sample collection schedule with marmosets treated with I.V. tacrolimus. n = 2 marmosets were challenged twice I.V. with rKSHV.219 (1 × 10^7^ iSLK IU) at the indicated timepoints. Marmosets were subsequently treated I.V. with tacrolimus (100 μg/kg) daily from D218 until the end of the study. Blood samples were collected throughout the study on the indicated timepoints; multiple tissues were collected for analysis at the indicated study endpoint. *Created in BioRender. Han, L. (2026) https://BioRender.com/t01n936*. **(B)** Quantification of LANA-1 gene copy numbers per µg of DNA isolated from PBMCs (i.e., PBMC viremia) of challenged marmosets over time using qPCR. Each datapoint represents the mean of two replicates Viral challenge and tacrolimus treatment initiation are indicated by green and red arrows, respectively. **(C-D)** Measurement of anti-KSHV VCA IgG (C) and anti-KSHV LANA-1 IgG (D) levels in plasma diluted 1/25 from challenged marmosets over time using ELISA. Each datapoint represents the mean of two replicates. Cut-off values were calculated as described in Materials and Methods and are represented using a dotted line. Viral challenge and tacrolimus treatment initiation are indicated by green and red arrows, respectively. **(E-F)** Quantification of LANA-1 gene DNA copy numbers per µg of DNA (i.e., KSHV viral load) isolated from various lymphoid (E) and non-lymphoid (F) tissues of challenged marmosets at euthanasia using qPCR. Each bar represents the mean of two replicates. **(G)** Experimental marmoset treatment, sample collection schedule and quantification of tacrolimus blood levels in marmosets treated with oral tacrolimus. Left: n = 2 marmosets were challenged once I.V. with rKSHV.219 (2 × 10^7^ HEK-293 IU). Marmosets were subsequently treated orally with tacrolimus (0.3 mg/kg) daily beginning on D87, after which the daily tacrolimus dose was increased on D192 (0.4 mg/kg) and again on D213 (0.8 mg/kg), and administered until the end of the study. Blood samples were collected throughout the study on the indicated timepoints; multiple tissues were collected for analysis at the indicated study endpoint. Right: Tacrolimus blood levels were measured on D180 and D212. *Created in BioRender. Han, L. (2026) https://BioRender.com/r0qo4u5*. **(H-I)** Quantification of LANA-1 gene DNA copy numbers per µg of DNA (i.e., KSHV viral load) isolated from various lymphoid (H) and non-lymphoid (I) tissues of challenged marmosets at euthanasia using qPCR. Each bar represents the mean of two replicates.

Regarding the general health of the marmosets during the study, we noted that daily intravenous administration of tacrolimus resulted in several adverse events, including reduced appetite, weight loss, and loss of mobility, particularly in the hind limbs due to pain associated with the daily injections. To mitigate the adverse events observed with daily intravenous tacrolimus administration, we next evaluated the feasibility of oral tacrolimus delivery. We intravenously challenged two common marmosets at WNPRC, Cj2007 (female) and Cj1833 (male), with a single dose of rKSHV.219 (2 × 10⁷ HEK-293 IU) on D0 (Fig 1G). We initiated tacrolimus treatment on D87 and administered it daily with food at an initial dose of 0.3 mg/kg/day. When we measured blood levels of tacrolimus on D180, they were below the detection threshold (<2 ng/mL), well below the therapeutic range of 5–15 ng/mL required for effective immunosuppression [39,40]. As a result, we increased the dose to 0.4 mg/kg/day on D192, and further to 0.8 mg/kg/day on D213, continuing through the end of the protocol (D227); however, therapeutic levels were not achieved. Throughout the study, we detected neither PBMC viremia nor anti-KSHV VCA or LANA-1 IgG production at any of the blood collection timepoints (S1A-C Fig). However, in Cj1883 at necropsy, we did confirm KSHV infection was established, as evidenced by quantifiable KSHV viral load in spleen, bone marrow, and inguinal and submandibular lymph nodes (Fig 1H), as well as in liver and lung (Fig 1I), with traces of KSHV DNA also present in all other tested tissues (S10 Fig). We also confirmed KSHV infection was established in marmoset Cj2007 by detecting viral DNA in several tissues (S10 Fig), although viral load in this marmoset was only quantifiable in spleen and inguinal lymph nodes (Fig 1H, 1I), demonstrating a lower viral distribution among tissues. Regarding LANA-1 protein expression in the spleen as assessed by IHC, Cj1883 was classified as LANA-1 positive (IHC 2, S12 Fig), while Cj2007 was classified as inconclusive (IHC 1, S11A Fig).

Regarding the general health of the marmosets, oral tacrolimus was not associated with the severe adverse events previously observed for the intravenous route of administration, suggesting a more tolerable safety profile. However, oral administration failed to achieve blood levels within the therapeutic range, which may have limited its immunosuppressive efficacy.

Consistent with the mechanism of action of tacrolimus [41], which inhibits T cell activation without depleting the cellular population, we observed no significant changes in immune cell frequencies before or after tacrolimus administration in either study (S1D-E Fig). In addition, although we found a suspicious skin lesion in one marmoset (Cj062; intravenous tacrolimus), we could not confirm it as a KS-like skin lesion using conventional immunohistochemistry (IHC) staining against KSHV LANA-1 using the collected skin biopsies (S2 Fig).

Altogether, the data we obtained in this experimental cohort confirmed that the administration of 2 × 10⁷ IU of rKSHV-219, even in a single intravenous dose, is sufficient to establish KSHV infection in common marmosets. However, findings from both the intravenous and oral tacrolimus experiments underscore the challenges associated with using tacrolimus to achieve effective immunosuppression in this model. These limitations, together with the fact that KSHV pathologies are associated with immunosuppressive settings [5,6], highlighted the need to develop alternative immunosuppressive strategies, such as targeted T cell depletion using mAbs, to support sustained viral replication and pathogenesis studies in marmosets.

### Development of marmoset-chimerized anti-CD4 and anti-CD8 mAbs for *in vivo* T cell depletion in common marmosets

Over the past few decades, the ability of anti-CD4 and anti-CD8 mAb therapies to deplete CD4^+^ and CD8^+^ T cell populations in NHPs has been well established [42]. However, most of the NHP studies using this immunosuppression method have been conducted in rhesus macaques, and only a single prior report describes the use of anti-CD8 [6F10] in marmosets [37]. To establish an effective CD4^+^ and CD8^+^ T cell immunosuppressive regimen in marmosets, and given the limited availability of reported marmoset-specific antibodies, we first evaluated the capacity of seven distinct primate-reactive mAbs to bind to marmoset PBMCs (Fig 2A). These included three anti-CD4 mAbs: mouse anti-human CD4 [OKT4], rhesusized anti-human CD4 [CD4R1], and mouse anti-rhesus CD4 [19Thy5D7]; and four anti-CD8 mAbs: mouse anti-rhesus CD8alpha [7Pt-3F9], rhesusized anti-rhesus CD8beta [CD8b255R1], rhesusized anti-rhesus CD8alpha [M-T807R1], and mouse anti-marmoset CD8 [6F10]. By using flow cytometry analysis, we revealed that mouse anti-rhesus CD4 [19Thy5D7] and mouse anti-marmoset CD8 [6F10] exhibited the best binding to marmoset PBMCs, effectively detecting their respective target cell populations. These results identified these two mAbs as optimal candidates for subsequent T cell depletion studies in marmosets.

**Fig 2:**
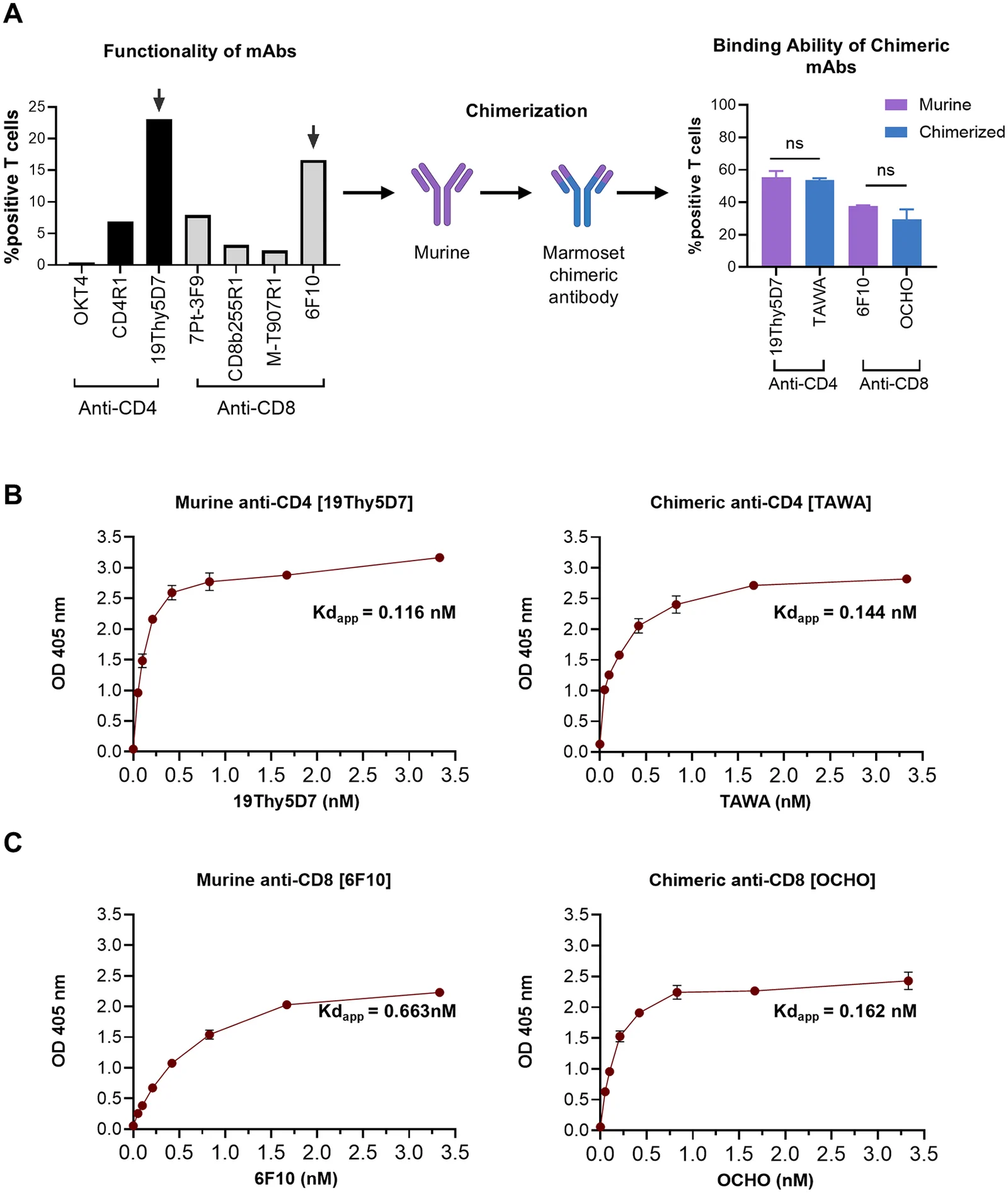
Confirmation of affinity retention of the selected anti-CD4 and anti-CD8 mAbs after marmoset-chimerization. **(A)** Quantification of CD4^+^ and CD8^+^ T cells in marmoset PBMCs by flow cytometry using murine mAbs before and after marmoset-chimerization. Multiple murine mAbs were tested for their ability to bind and detect CD4^+^ and CD8^+^ T cells in marmoset PBMCs by flow cytometry (left bar graph); each bar represents the percentage binding of one replicate. Anti-CD4 [19Thy5D7] and anti-CD8 [6F10] (indicated by arrows), which displayed the highest binding, underwent chimerization by replacement of the murine antibody constant regions with marmoset antibody constant regions (middle schematic). After chimerization, the ability of the new marmoset-chimerized mAbs anti-CD4 [TAWA] and anti-CD8 [OCHO] to bind T cells in marmoset PBMCs was re-tested using flow cytometry, and compared to the parental murine mAbs (right bar graph); each bar represents the mean ± standard deviation of three replicates. Statistical significance was determined using unpaired t-test; ns = not significant. **(B-C)** Comparison of binding affinity of anti-CD4 (B) and anti-CD8 (C) murine vs. marmoset-chimerized mAbs to rhesus CD4 (B) and marmoset CD8 (C) by ELISA. The apparent Kd values (Kd_app_) included in each graph were estimated by assessing serial dilutions of the murine parental mAbs (left) and their marmoset-chimerized versions (right). Kd_app_ was calculated in Graphpad Prism using non-linear regression and assuming a one-site specific binding. Each datapoint included in the graphs represents the mean of two replicates.

The use of murine mAbs for *in vivo* T cell depletion has been associated with the induction of anti-mouse antibodies, which can be toxic and lead to rapid mAb clearance, ultimately reducing therapeutic efficacy [43]. To circumvent this phenomenon, we generated marmoset-chimerized versions of the selected mouse anti-rhesus CD4 [19Thy5D7] and mouse anti-marmoset CD8 [6F10] antibodies (Fig 2A). Chimerization was performed in the Nonhuman Primate Reagent Resource (NHPRR) laboratory at MassBiologics under the guidance of Dr. Engelman and involved replacing all murine IgG constant regions with marmoset IgG constant regions. We renamed the resulting chimeric mAbs as anti-CD4 [TAWA] and anti-CD8 [OCHO]. We used flow cytometry analysis to confirm that both chimeric mAbs retained their binding capacity for marmoset PBMCs (Fig 2A). Moreover, the binding affinities (apparent Kd, Kd_app_) estimated by ELISA (Fig 2B, 2C) demonstrated that anti-CD4 [TAWA] retained comparable CD4 affinity to that of mouse anti-CD4 [19Thy5D7] (Fig 2B), and anti-CD8 [OCHO] displayed even greater CD8 affinity than that of mouse anti-CD8 [6F10], observed as a lower Kd_app_ value (Fig 2C). This latter improvement could be due to the greater purity of the recombinantly produced chimeric antibody relative to the hybridoma-derived parental clone [44].

To validate the specificity of anti-CD4 [TAWA] and anti-CD8 [OCHO] for their respective target populations, we performed a second flow cytometry analysis using marmoset splenocytes, incorporating co-staining with anti-CD3 [Sp34-2] (S3A-B Fig). In parallel, we stained additional marmoset splenocytes with anti-CD3 [Sp34-2] and either anti-CD4 [L200] or anti-CD8 [HIT8a], well-characterized anti-human antibodies used to define CD4⁺ and CD8⁺ T cell populations, respectively, with reported marmoset cross-reactivity (S1 Table) [45–47]. We included human PBMCs as positive controls to confirm the overall panel performance. We found that anti-CD4 [TAWA] recognized T cell populations at similar frequencies in marmoset splenocytes compared to anti-CD4 [L200] (S3A Fig), as did anti-CD8 [OCHO] compared to anti-CD8 [HIT8a] (S3B Fig). Additionally, we used density dot plots to demonstrate that both anti-CD4 [TAWA] (S3A Fig) and anti-CD8 [OCHO] (S3B Fig) exhibited binding to only a small subset of CD3^−^ cells, confirming T cells are their main target. Although recognition of other CD8⁺ cell types such as NK cells can explain the binding of anti-CD8 [OCHO] to a small CD3^−^ cell population [48], the recognition of a small CD4^+^CD3^−^ cell population could be due to the presence of accessory cells in B- and T-zone stroma in spleen [49, 50].

Because our *in vivo* study required longitudinal monitoring of CD4^+^ and CD8^+^ T cell population frequencies using flow cytometry, it was critical to ensure that the antibodies included in the flow cytometry panel would not be hindered by the anti-CD4 [TAWA] and/or anti-CD8 [OCHO] mAbs used for depletion through possible epitope competition. To address this, we performed a competition assay between conjugated versions of the marmoset chimeras, anti-CD4 [TAWA]-AF647 and anti-CD8 [OCHO]-AF488, and the flow cytometry mAbs, anti-CD4 [L200]-PerCP-Cy5.5 and anti-CD8 [HIT8a]-BUV805, respectively (S3C-D Fig). We stained marmoset splenocytes under various conditions: co-incubation of both anti-CD4 (TAWA + L200) or both anti-CD8 (OCHO + HIT8a) antibodies; sequential staining with the chimeric mAb followed by the flow cytometry mAb (TAWA → L200 or OCHO → HIT8a); the reverse sequential order (L200 → TAWA or HIT8a → OCHO); and single staining with each antibody as controls to determine maximal signal. We co-stained all samples with anti-CD3 to specifically identify T cell populations. We found the binding of anti-CD4 [L200] to be preserved among all tested conditions (S3C Fig, right panel), including the sequential “TAWA → L200” setup, which reflects the expected *in vivo* experimental conditions. Notably, anti-CD4 [TAWA] binding remained stable in this condition as well (S3C Fig, left panel). However, we observed reduced anti-CD4 [TAWA] signal when L200 was co- or pre-incubated, suggesting potential steric or allosteric interference between the two antibodies [51], thus future studies should be cautious regarding the order of incubation of these two mAbs. In contrast, we observed no competition between anti-CD8 [OCHO] and anti-CD8 [HIT8a], as both antibodies maintained consistent binding across all experimental conditions (S3D Fig, **left and right panels**), indicating non-overlapping epitopes.

Finally, to leverage flow cytometry to study changes in immune cell populations in subsequent experiments in which anti-CD4 [TAWA] and anti-CD8 [OCHO] are used as immunosuppressive tools, we tested an antibody panel composed of previously reported marmoset cross-reactive antibodies [45–47] originally developed for human or rhesus macaque cellular markers (S1 Table), in the absence of marmoset-specific mAbs. This panel included the compatible anti-CD4 [L200] and anti-CD8 [HIT8a] mAbs and we applied it in parallel in marmoset and human samples using the gating strategies described in S4A Fig and S4B Fig, respectively. This resulted in the successful identification of major immune cell subsets in both species, including T cells (CD3^+^), B cells (CD20^+^), classical monocytes (CD14^+^CD16^-^), non-classical monocytes (CD14^+^CD16^+^), and NK cells (CD3^-^CD20^-^, CD8^+/-^CD56^+^). To validate the accuracy of the panel, we assessed these major immune cell subsets in PBMCs and splenocytes from five marmosets and PBMCs from five human donors (S4C Fig). The frequencies of the studied immune cell populations fell within the expected normal ranges for both marmoset [46,52,53] and human [54] samples, confirming the panel’s reliability.

In summary, our results support the use of anti-CD4 [TAWA] and anti-CD8 [OCHO] as tools for *in vivo* T cell depletion in marmosets and the use of an antibody panel including anti-CD4 [L200] and anti-CD8 [HIT8a] as a reliable resource for post-depletion flow cytometry analysis in this model.

### Establishment of KSHV infection in common marmosets treated with a novel mAb-based CD4^+^ and CD8^+^ T cell depletion regimen

We evaluated the *in vivo* effect of our novel marmoset-chimerized mAbs on T cell depletion and subsequent establishment of KSHV infection in four common marmosets (females: Cj2077, Cj2279; males: Cj1983, Cj2245) at WNPRC. All marmosets were intravenously challenged with rKSHV.219 (5 × 10^5^ HEK-293 IU) on D0, after receiving an intravenous cocktail of anti-CD4 [TAWA] and anti-CD8 [OCHO], each administered at a dose of 10 mg/kg body weight, on D-14, D-9, and D-7 (Fig 3A).

**Fig 3:**
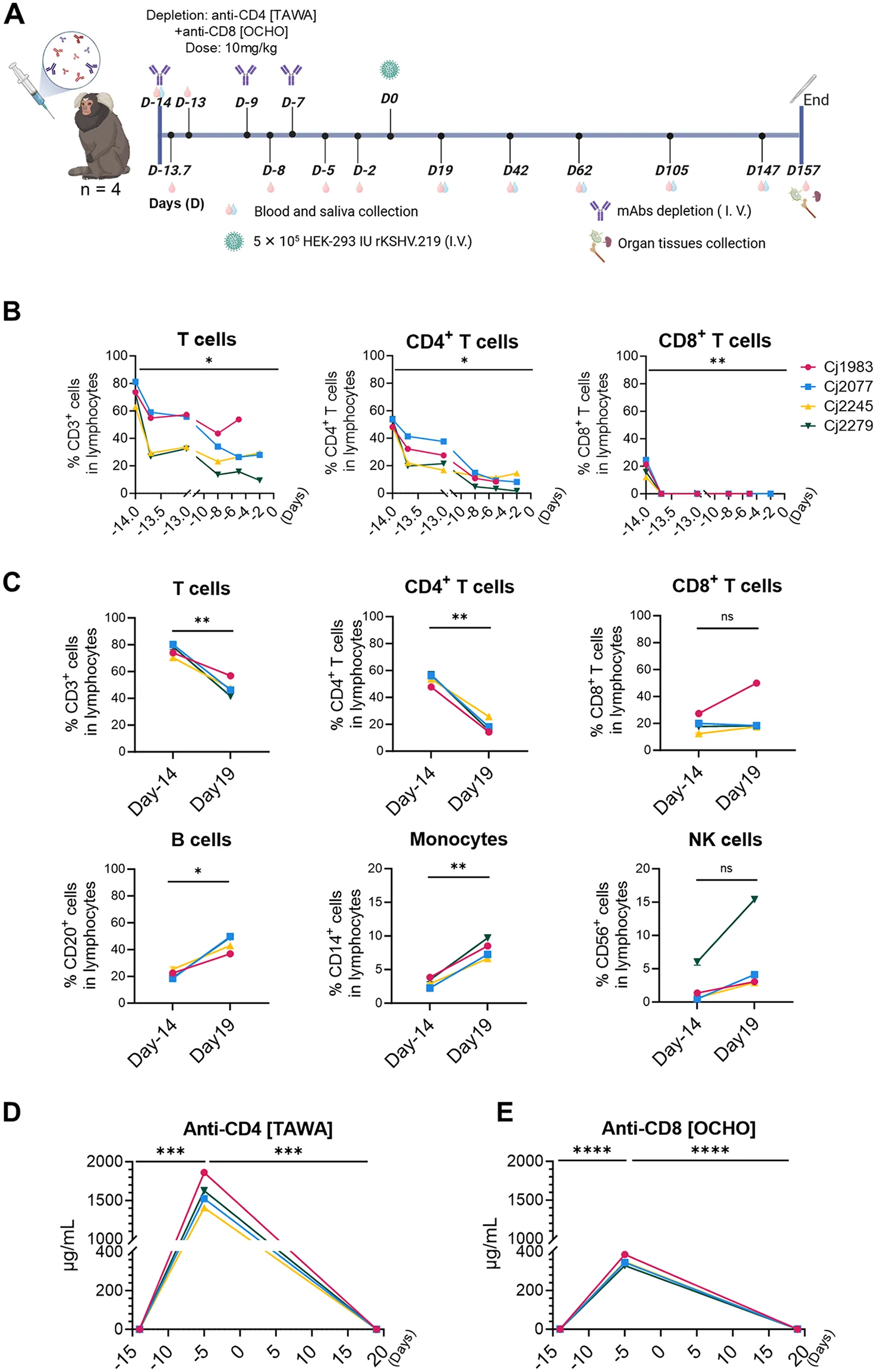
Chimeric mAb-mediated immunosuppression after cocktail administration, and intravenous (I.V.) rKSHV infection of common marmosets. **(A)** Experimental marmoset treatment and sample collection schedule. n = 4 marmosets were treated I.V. with a cocktail of the chimeric mAbs anti-CD4 [TAWA] and anti-CD8 [OCHO] (10 mg/kg each) on the indicated timepoints, beginning on D-14. Marmosets were subsequently challenged I.V. with rKSHV.219 (5 × 10^5^ HEK-293 IU) on D0. Blood samples were collected throughout the study on the indicated timepoints; multiple tissues were collected for analysis at the indicated study endpoint. *Created in BioRender. Han, L. (2026) https://BioRender.com/wbmj2i2*. **(B)** Quantification of total T cells (CD3^+^), CD4^+^ and CD8^+^ T cells in the PBMCs of mAb-treated marmosets over time using flow cytometry. Each datapoint represents the mean of three technical replicates. Statistical significance was determined using one-sample t-test followed by paired t-test; * = p < 0.05, ** = p < 0.01. *Note that these results were obtained prior to flow cytometry panel optimization and CD8*^*+*^ *T cell population frequency could be underestimated, thus results should be interpreted with caution (refer to the main text for clarity).* **(C)** Quantification of total T cell (CD3^+^), CD4^+^ T cell, CD8^+^ T cell, B cell (CD20^+^), monocyte (CD14^+^), and NK cell (CD56^+^) populations in PBMCs of mAb-treated marmosets on D-14 and D19 using a validated antibody panel for flow cytometry (S1 Table). Each datapoint represents the mean of three technical replicates. Statistical significance was determined using paired t-test; ns = not significant, * = p < 0.05, ** = p < 0.01. **(D-E)** Quantification of anti-CD4 [TAWA] **(D)** and anti-CD8 [OCHO] **(E)** plasma levels in mAb-treated marmosets over time using ELISA. Each datapoint represents the mean of two technical replicates. Statistical significance was determined by one-sample t-test, followed by the paired t-test; *** = p < 0.001, **** = p < 0.0001.

To assess the efficacy of the immunosuppressive regimen, we first used flow cytometry to evaluate the variation in T cell populations in PBMCs collected from D-14 to D-2 (Fig 3B). It should be noted that this analysis was performed before validating the flow cytometry panel described above (S1 Table), and samples were stained using only anti-CD3 [SP34-2], anti-CD4 [L200], and anti-CD8 [6F10], the latter being the parental clone of anti-CD8 [OCHO] and the only mAb available in our group to bind to marmoset CD8^+^ cells at that time. From D-14 to D-2, we observed a persistent decline in the frequency of total T cells (CD3^+^) (Fig 3B, left panel) and CD4^+^ T cells (Fig 3B, center panel). Remarkably, the CD8^+^ T cell population (Fig 3B, right panel) showed an apparent complete depletion within 8 h after mAb administration, which was maintained until D-2. However, considering that anti-CD8 [OCHO] and anti-CD8 [6F10] bind to the same epitope, the possible systemic presence of the marmoset-chimerized anti-CD8 could have resulted in an inaccurate evaluation of the CD8^+^ T cell population in this assay. To address this limitation, once we validated the flow cytometry panel (S1 Table), we re-evaluated the immune cell populations using the remaining PBMCs, which were only available for D-14, corresponding to the first dose, and D19, corresponding to 1-month post-final cocktail dose (Fig 3C). We observed a similarly significant decrease in the frequency of both total T cells (CD3^+^) and CD4^+^ T cells from D-14 to D19, but saw no significant variation in the frequency of the CD8^+^ T cell population. When we analyzed other immune subsets on D19 (Fig 3C, bottom panels), we observed an apparent increase in the frequencies of B cells, monocytes, and NK cells. Although these results show that treatment with anti‑CD4 [TAWA] and anti‑CD8 [OCHO] did not deplete other lymphocyte subsets, the apparent increasing trend in those populations should be interpreted with caution. Because all data are expressed as a percentage of total lymphocytes, a reduction in T cell frequency can artifactually appear as an increase in the relative frequency of other, otherwise stable, lymphocyte populations.

Next, we used ELISA to assess the systemic presence of anti-CD4 [TAWA] and anti-CD8 [OCHO] in plasma samples collected at three timepoints: pre-depletion (D-14), two days after the third cocktail dose (D-5), and 1 month after the final cocktail dose (D19) (Fig 3D, 3E). Plasma antibody levels were undetectable for both mAbs on D-14 and D19. On D-5, anti-CD4 [TAWA] reached an average concentration of 1605.0 µg/mL (Fig 3D) and anti-CD8 [OCHO] reached an average concentration of 351.7 µg/mL (Fig 3E). Despite both antibodies being administered at identical doses (10 mg/kg), the plasma concentration of anti-CD4 [TAWA] was approximately 4.5-fold higher than that of anti-CD8 [OCHO] on D-5. This disparity may reflect a more rapid clearance or greater *in vivo* consumption of anti-CD8 [OCHO].

These results show that anti-CD4 [TAWA] could successfully deplete the CD4^+^ T cell population for at least 1 month when using the tested administration regimen. Although it was not possible to directly confirm efficient depletion of the CD8^+^ T cell population, the systemic availability of anti-CD8 [OCHO] by D-5, together with previous reports from other authors demonstrating the capacity of the parental clone (6F10) to deplete this cell population in marmosets [37], plausibly suggest the CD8^+^ T cell population was reduced by the time of infection (D0).

Given the importance of precisely defining the immune status of the marmosets at the time of KSHV infection and the lack of additional PBMC samples available for testing due to sampling blood volume limits inherent to this animal model [30], we assessed anti-CD4 [TAWA] and anti-CD8 [OCHO] immunosuppression in a separate subset of marmosets allocated to a non-related project at the Texas Biomedical Research Institute Southwest National Primate Research Center (SNPRC). We assigned nine marmosets to two groups: three females and two males to an immunocompetent group that received PBS as a negative control and three females and one male to an immunosuppressed group that received a cocktail of anti-CD4 [TAWA] and anti-CD8 [OCHO] at the same dose as before (10 mg each/kg body weight) using a functionally equivalent passive immunization schedule as in our previous KSHV experimental cohort in Fig 3A. Marmosets were bled at baseline before starting the immunosuppression regimen (D0), and then on D14, D28, and D56 post-final cocktail dose (Fig 4A).

**Fig 4:**
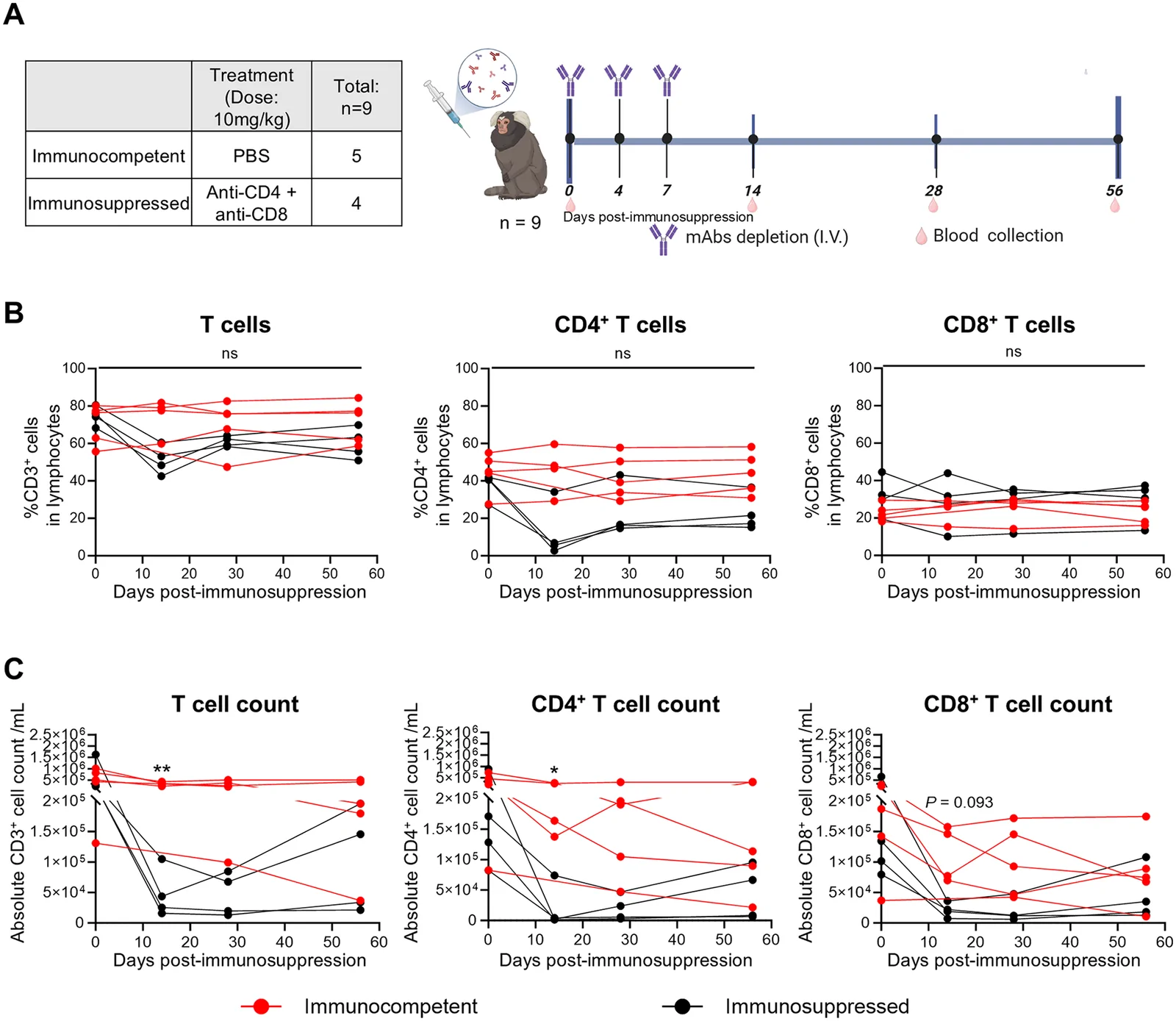
Confirmation of effective anti-CD4 [TAWA] and anti-CD8 [OCHO]-mediated depletion of CD4 and CD8 T cells in marmoset lymphocytes *in vivo.* **(A)** Experimental marmoset immunosuppression and sample collection schedule. n = 9 marmosets were divided into two groups: an immunocompetent group (n = 5) and an immunosuppressed group (n = 4). The immunosuppressed group was treated intravenously (I.V.) with a cocktail of the chimeric mAbs anti-CD4 [TAWA] and anti-CD8 [OCHO] (10 mg/kg each) on the indicated timepoints. Blood samples were collected on the indicated timepoints. *Created in BioRender. Han, L. (2026)* **https://BioRender.com/we2o9qj*.* **(B)** Quantification of total T cells (CD3^+^), CD4^+^, and CD8^+^ T cells in PBMCs of mAb-treated marmosets over time using flow cytometry. Each datapoint represents the mean of three replicates. Statistical significance of immunosuppressed vs. immunocompetent groups at each timepoint was determined by one-sample t-test, followed by the paired t-test; ns = not significant. **(C)** Derived absolute cell counts of total T cells (CD3^+^), CD4^+,^ and CD8^+^ T cells per mL of blood. These values were calculated by applying the frequency values obtained by flow cytometry to the number of PBMCs obtained per mL of blood as reported by SNPRC. Each datapoint represents the mean of three replicates. Statistical significance of immunosuppressed vs. immunocompetent groups at each timepoint was determined by two-way ANOVA; * = p < 0.05, ** = p < 0.01.

We used flow cytometry to monitor the frequency of T cell populations in PBMCs over the study period. In the immunocompetent group, T cell populations remained stable (Fig 4B). In contrast, in the immunosuppressed group, we observed a decreasing trend in the frequency of total T cells (CD3^+^) and CD4^+^ T cells by D14, followed by a gradual recovery, although differences between the two groups did not reach statistical significance when assessed by frequency alone. Because frequency-based analysis can mask changes if there are slight variations in other leukocyte populations present, and because cell count data (PBMCs/mL of blood) were available for each timepoint in this experiment (S5A Fig), we re-analyzed the data using absolute cell counts. We confirmed that absolute numbers of CD3^+^ (*p < 0.01*) and CD4^+^ (*p < 0.05*) T cells were significantly reduced at D14 in the immunosuppressed group compared to the immunocompetent group (Fig 4C). CD8^+^ T cell counts showed a similar, strongly consistent trend across treated marmosets (p = 0.093). By D28, all T cell populations showed evidence of recovery, which continued through the final timepoint (D56). Consistent with our initial analysis, we observed no differences in the frequencies (S5B Fig) or absolute counts (S5C Fig) of B cells, NK cells, or monocytes between groups at any timepoint. Collectively, these data emphasize the value of using absolute cell counts in depletion studies and strongly suggest that both CD4⁺ and CD8⁺ T cell populations were efficiently but transiently depleted in our KSHV infection experiment using mAb-mediated immunosuppression.

To determine whether KSHV infection was successfully established in the four immunosuppressed marmosets included in our original chimeric mAb cocktail experiment (Fig 3A), we monitored PBMC viremia, seroconversion, and KSHV viral load in saliva during the entire length of the study. Additionally, we assessed tissue viral loads and LANA-1 expression in the spleen at endpoint. We did not detect PBMC viremia in any marmoset at any of the blood collection timepoints throughout the study, except at the endpoint (D157), wherein we detected traces of LANA-1 in PBMCs from all four marmosets, as shown in the summary heatmap (S10 Fig). Only Cj2245 showed notable seroconversion, evidenced by an increase in anti-KSHV VCA IgG production between D42 and D62 (Fig 5A); no marmoset displayed anti-KSHV LANA-1 IgG (Fig 5B). However, when we used qPCR to quantify KSHV viral load in DNA extracted from marmoset saliva, each marmoset presented at least one peak in KSHV viral load in saliva between D19 and D42 (Fig 5C). Cj2245 also showed a second and higher peak at D62, which paralleled the observed increase in anti-KSHV VCA IgG. Together, these results suggest that experimental KSHV infection in marmosets can undergo lytic replication and salivary shedding as is observed in humans. Additionally, we observed detectable KSHV viral load in splenocytes from all four marmosets, at levels ranging from 10 to 500 copies/µg of DNA (Fig 5D). As a negative control, we confirmed that LANA-1 could not be detected in splenocytes collected at SNPRC from six unrelated, uninfected marmosets (Fig 5D). Infection in spleen was confirmed by KSHV LANA-1 IHC in two out of four marmosets (Cj1983 and Cj2279, S11A Fig and S12 Fig). Although we detected the presence of KSHV across lymphoid tissues in all four rKSHV-infected marmosets (S10 Fig), we observed quantifiable viral loads only in the bone marrow and mesocolic lymph nodes of Cj2279 (Fig 5E). Notably, among non-lymphoid tissues, we detected KSHV viral load in the livers of all four marmosets (Fig 5F) and observed traces in the lungs of three marmosets (S10 Fig).

**Fig 5:**
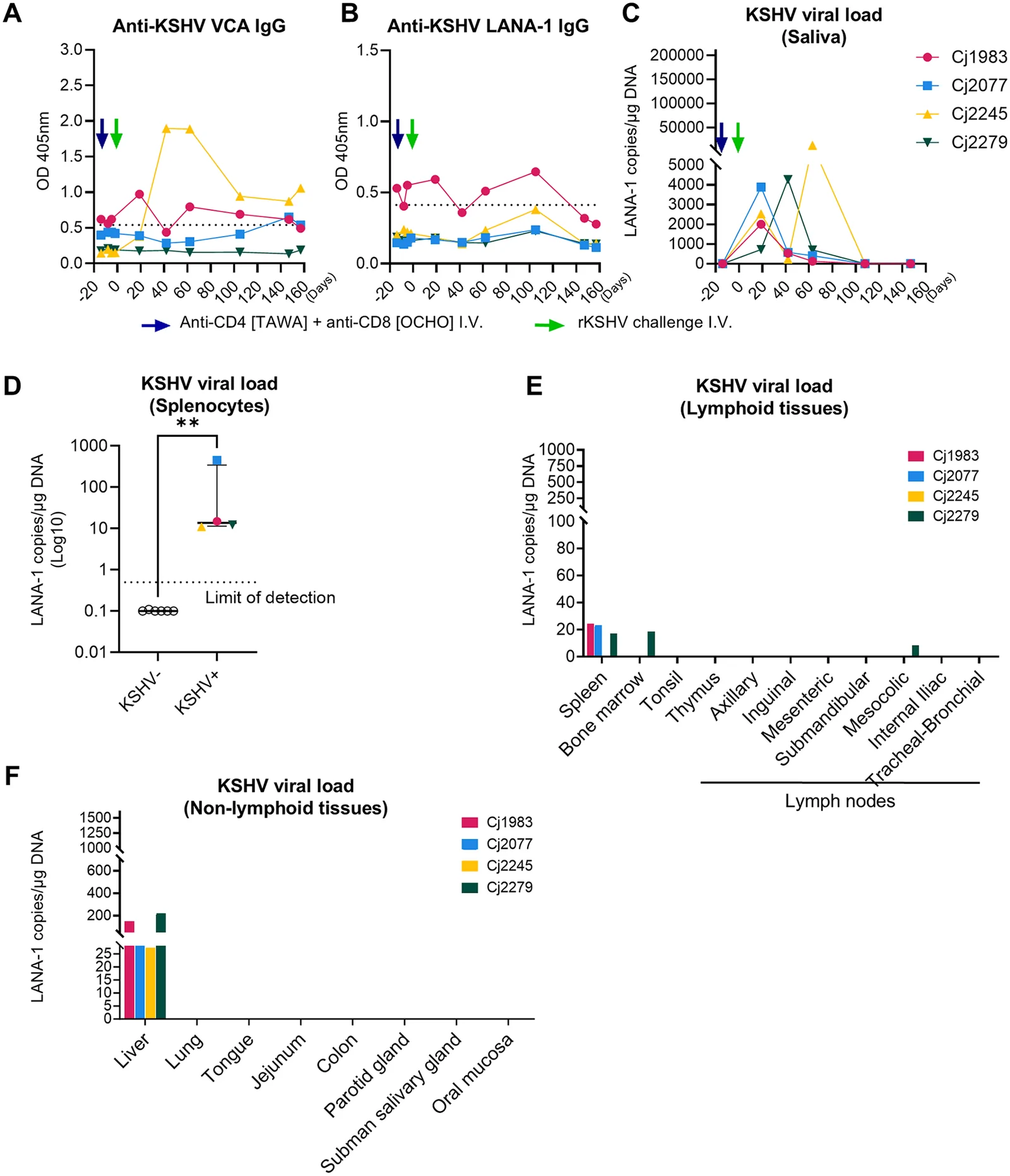
Anti-KSHV VCA and anti-KSHV LANA-1 IgG production and KSHV viral loads in chimeric mAb cocktail-treated marmosets upon intravenous (I.V.) rKSHV challenge. **(A-B)** Measurement of anti-KSHV VCA IgG **(A)** and anti-KSHV LANA-1 IgG **(B)** levels in plasma diluted 1/25 from challenged marmosets over time using ELISA. Each datapoint represents the mean of two technical replicates. Cut-off value was calculated as described in Materials and methods and are represented using a dotted line. **(C)** Quantification of LANA-1 gene copy numbers per µg of DNA isolated from saliva (i.e., KSHV viral load) of challenged marmosets (described in Fig 3) over time using qPCR. Each datapoint represents the mean of four technical replicates. **(D)** Quantification of LANA-1 gene DNA copy numbers per µg of DNA isolated from splenocytes (i.e., KSHV viral load) of challenged marmosets at euthanasia (KSHV+, described in Fig 3) using qPCR. KSHV viral load was also quantified in splenocytes from n = 6 unrelated, uninfected marmosets (KSHV–) as a control. Each datapoint represents an individual marmoset; the median and interquartile range are indicated for each group. Statistical significance was determined using two-tailed Mann-Whitney test; ** = p < 0.01. **(E-F)** Quantification of LANA-1 gene DNA copy numbers per µg of DNA (i.e., KSHV viral load) isolated from various lymphoid **(E)** and non-lymphoid **(F)** tissues of challenged marmosets at euthanasia (described in Fig 3) using qPCR. Each bar represents the mean of two technical replicates.

Together, our data demonstrates the successful depletion of CD4⁺ T cells upon administration of anti-CD4 [TAWA], and suggest a similar outcome for CD8^+^ T cells upon administration of anti-CD8 [OCHO]. Furthermore, these data confirm that KSHV infection can be established in common marmosets following a single viral challenge, with possible viral replication and broad tissue dissemination detectable within a three-month period.

### CD8^+^ T cell depletion at the time of KSHV infection corresponds with episodes of active KSHV lytic replication in saliva in common marmosets

To further characterize the effect of immune depletion mediated by anti-CD4 [TAWA] and anti-CD8 [OCHO] on establishment of KSHV infection in marmosets, we conducted a follow-up study at WNPRC using 22 female and male marmosets assigned to four treatment groups (see S10 Fig for specific sex distribution): “anti-CD4” (anti-CD4 [TAWA] only), “anti-CD8” (anti-CD8 [OCHO] only), “anti-CD4 f/b anti-CD8” (anti-CD4 [TAWA] followed by anti-CD8 [OCHO]), and “PBS” (non-depleted control group). The immunosuppression regimens were administered before KSHV challenge, and consisted of three intravenous doses of 10 mg/kg body weight of the corresponding mAb (anti-CD4 [TAWA] or anti-CD8 [OCHO]) on D-21, D-19, and D-17, whereas the control group received an equivalent volume of PBS on the same days (S6A Fig). In the anti-CD4 f/b anti-CD8 group, after the above administration of anti-CD4 [TAWA], marmosets received three doses of anti-CD8 [OCHO] on D-12, D-10, and D-7. All marmosets were challenged intravenously with rKSHV BAC16 (5 × 10^5^ HEK-293 IU) on D0.

We first used flow cytometry to assess the impact of the three immunosuppressive regimens on immune cell populations at baseline (D-21), at the time of KSHV challenge (D0), and at the study endpoint (D147) (S6 Fig). For the sequential treatment group (anti-CD4 f/b anti-CD8), an additional checkpoint was included on D-12 to capture the effects of anti-CD4 [TAWA] prior to initiation of anti-CD8 [OCHO] administration. A significant decrease in total T cells (CD3⁺) by D0 was only observed in the anti-CD4 f/b anti-CD8 group (S6B Fig, top panels). For CD4⁺ T cell frequencies, in contrast to our previous experiment (Fig 3), we observed no significant reduction of this population in the anti-CD4 [TAWA]-treated groups after this mAb administration (S6B Fig, middle panels). However, CD8⁺ T cells were significantly depleted by D0 in all marmosets treated with anti-CD8 [OCHO], regardless of group assignment, with frequencies returning to baseline by the endpoint (S6B Fig, bottom panels).

Considering the discrepancy in anti-CD4 [TAWA] treatment efficacy between this and our previous experiments, we measured the levels of marmoset-chimerized mAbs in the plasma of treated marmosets (S7A Fig). At D0, we detected anti-CD8 [OCHO] in all anti-CD8 [OCHO]-treated marmosets, whereas anti-CD4 [TAWA] was undetectable in all anti-CD4 [TAWA]-treated marmoset at all tested timepoints, indicating a possible degradation or loss of functionality of the administered mAb. Given that we observed effective CD4^+^ T cell depletion upon anti-CD4 [TAWA] administration in our previous experiment (Fig 3) and that we used the same mAb batch (Lot JP2222101) to conduct the present experiment, we investigated whether the loss of efficacy was due to impaired antigen binding. To assess this, we compared the binding capacity of the batch used in both marmoset KSHV challenge studies (Lot JP2222101) to a newly produced batch (Lot 00128) obtained from NHPRR. We used ELISA to evaluate both batches of the marmoset-chimerized anti-CD4 [TAWA], using recombinant rhesus CD4 as the target antigen and the parental mouse anti-CD4 [19Thy5D7] clone as a positive control (S7B Fig). The newly produced Lot 00128 presented strong binding activity, yielding optical density (OD) values comparable to those of the parental clone. In contrast, Lot JP2222101 failed to bind the CD4 antigen, indicating a loss of functionality. These findings may explain the lack of CD4⁺ T cell depletion we observed in the present challenge study (S6A Fig) and underscore the importance of performing independent functional validation of antibody lots prior to every use in *in vivo* depletion protocols, regardless of their previous confirmed functionality.

In light of these findings, we could not fully meet our original experimental objectives due to the loss of anti‑CD4 [TAWA] functionality. Nonetheless, the successful depletion of CD8⁺ T cells (anti-CD8 and anti-CD4 f/b anti-CD8 groups) at two distinct timepoints relative to KSHV infection provided a valuable opportunity to address alternative questions, and we therefore restructured the experimental groups to maximize scientific yield and ensure responsible and ethical use of animal resources.

Under this context, we subsequently focused on KSHV infection outcomes in the two CD8⁺ T cell-depleted groups that received three intravenous doses of functional anti‑CD8 [OCHO] (10 mg/kg) (Fig 6A), which we renamed as follows: the original anti-CD8 group, dosed on D-21, D-19, and D-17, was renamed “anti‑CD8 (initiated D‑21)” and the original anti-CD4 f/b anti-CD8 group, dosed on D-12, D-10, and D-7, was renamed “anti‑CD8 (initiated D‑12).” We compared these groups to the two non‑depleted controls: “mock anti‑CD4,” which received parallel doses of non‑functional anti‑CD4 [TAWA] (originally the anti-CD4 group); and “PBS,” which received PBS alone. We challenged all marmosets intravenously with rKSHV BAC16 (5 × 10^5^ HEK‑293 IU) on D0.

**Fig 6:**
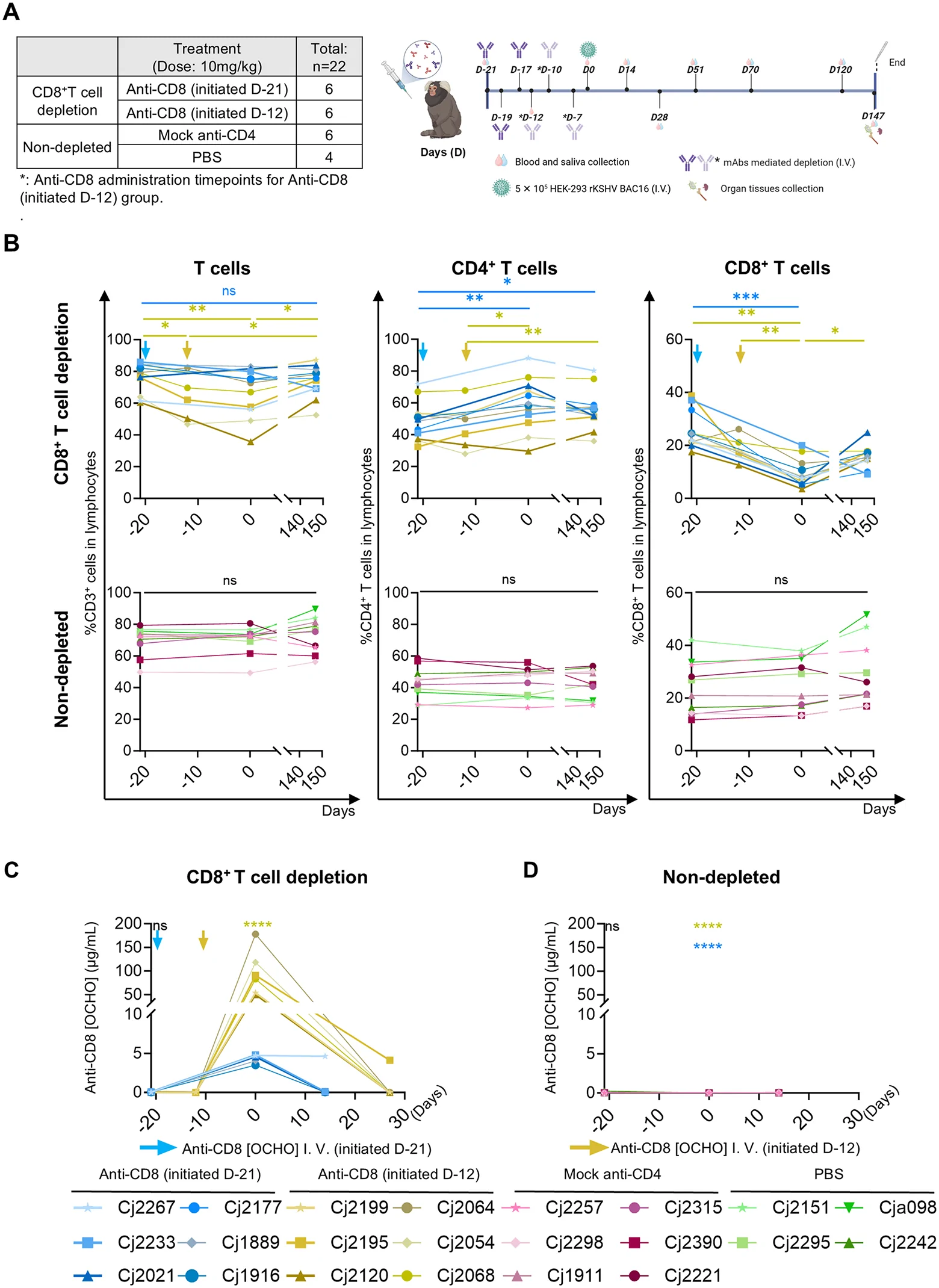
Chimeric mAb anti-CD8 [OCHO]-mediated immunosuppression and intravenous (I.V.) rKSHV infection of common marmosets. **(A)** Experimental marmoset treatment and sample collection schedule. n = 22 marmosets were distributed into 4 treatment groups, as indicated on the treatment group summary table on the left of the panel; *note that these are the same marmosets described in* *S6 Fig* *under a different group structure.* Marmosets with CD8^+^ T cell depletion were treated I.V. with anti-CD8 [OCHO] initiated at D-21 or D-12, while non-depleted groups were treated I.V. with non-functional anti-CD4 [TAWA] (mock) or PBS. The CD8^+^ T cell depletion groups received 10 mg/kg mAb on the indicated timepoints. Marmosets were subsequently challenged I.V. with rKSHV BAC16 (5 × 10^5^ HEK-293 IU) on D0. Blood samples were collected throughout the study on the indicated timepoints; multiple tissues were collected for analysis at the indicated study endpoint. *Created in BioRender. Han, L. (2026) https://BioRender.com/kyk51dr*. **(B)** Quantification of total T cells (CD3^+^), CD4^+^ and CD8^+^ T cells in PBMCs of mAb-treated marmosets over time using flow cytometry. Each datapoint represents the mean of three technical replicates. Statistical significance between two timepoints within the same group was determined using paired t-test; ns = not significant, * = p < 0.05, ** = p < 0.01. Matching colors are used to denotate differences within a single group. **(C)** Quantification of anti-CD8 [OCHO] plasma levels (μg/mL) in all marmoset treatment groups over time using ELISA. Each datapoint represents the mean of two technical replicates. Statistical significance between two groups at each timepoint was determined using two-way ANOVA followed by multiple comparison test; ns = not significant, **** = p < 0.0001. In (B-C), anti-CD8[OCHO] mAb treatment initiations at D-21 or D-12 are indicated by blue and yellow arrows, respectively. Asterisk colors identified the significantly higher value to its counterpart.

Using flow cytometry analysis, we found comparable reductions in CD8⁺ T cell frequencies at the time of KSHV challenge (D0) in both anti-CD8 [OCHO]-treated groups, with partial to complete recovery observed in most marmosets by study endpoint (D147) (Fig 6B). Concurrently, these groups exhibited an opposite trend (i.e., increase) in CD4⁺ T cells (Fig 6B), B cells, and NK cells (S8A Fig). Although these data confirm the selective targeting of CD8⁺ T cells by anti-CD8 [OCHO], the observed increases in the frequencies of non-target populations should be interpreted with caution in the absence of total PBMC counts, as noted in the previous section. Despite exhibiting similar cellular profiles at D0, the anti-CD8 (initiated D-12) group displayed significantly greater systemic anti-CD8 [OCHO] concentrations than did the anti-CD8 (initiated D-21) group (Fig 6C), attributable to the shorter interval between final antibody dosing and challenge (7 vs. 17 days). Due to a lack of PBMCs between D0 and the study endpoint (D147), we could not measure cellular populations at intermediate timepoints; however, the elevated systemic antibody levels, combined with our previous observations on CD8⁺ T cell recovery kinetics, suggest that a more prolonged post-challenge depletion could have occurred in the anti-CD8 (initiated D-12) group vs. the anti-CD8 (initiated D-21) group. Marmosets in both non-depleted control groups exhibited stable frequencies of lymphocyte subsets over time (Fig 6B, S8A Fig), except for an increase in NK cells and a decrease in B cells after KSHV infection (p < 0.01 and p < 0.05, respectively), with no detectable circulating anti-CD8 [OCHO] (Fig 6D) at any tested timepoints.

To assess differences in infection outcomes between immunocompetent and CD8⁺ T cell-depleted marmosets over the study period, we monitored PBMC viremia, KSHV viral load in saliva, and seroconversion. Although we did not detect PBMC viremia in any groups at any of the blood collection timepoints (S8B Fig), we observed a peak in KSHV viral load in saliva in almost all CD8⁺ T cell-depleted marmosets (Fig 7A), but not in any non-depleted marmoset, at all the tested timepoints. Specifically, we detected KSHV viral load in saliva (i.e., salivary shedding) from both anti-CD8 [OCHO]-treated groups after ≥14 days of undetectability post-challenge, suggesting the observable peaks could correspond to episodes of viral lytic replication. Interestingly, KSHV salivary shedding kinetics were consistent between marmosets in the same group but varied between the two distinct anti-CD8 [OCHO]-treated groups. In the anti-CD8 (initiated D-12) group, viral load peaked at D51 and reached >20,000 LANA-1 copies/µg DNA in all marmosets, whereas in the anti-CD8 (initiated D-21) group, the peak was delayed to D70 and reached a maximum of only 10,000 LANA-1 copies/µg DNA, with one of the six marmosets (Cj1916) showing no shedding. Remarkably, salivary viral load corresponded with anti-KSHV VCA seroconversion (Fig 7B). All marmosets in the non-depleted groups exhibited early peaks of anti-KSHV VCA IgG near the challenge timepoint, followed by plateauing at <1 OD (Fig 7B right panel). Considering these marmosets did not show signs of viral lytic replication in blood or saliva, the measured specific IgG levels could be consistent with basal seroconversion from primary exposure during KSHV challenge without episodes of lytic replication. In contrast, most anti-CD8 (initiated D-12) marmosets maintained elevated anti-KSHV VCA IgG levels, suggesting prolonged KSHV antigen exposure, possibly due to active lytic replication upon challenge (Fig 7B left panel). However, only one of five anti-CD8 (initiated D-21) marmosets followed this pattern. These data indicate that sustained CD8⁺ T cell depletion post-challenge may be critical for enabling KSHV lytic replication in marmosets. Regarding the presence of antibodies against the latent antigen LANA-1 (Fig 7C), only Cj2195 in the anti-CD8 (initiated D-12) group displayed a rise in anti-KSHV LANA-1 IgG (Fig 7C left panel), which coincided with a decrease in anti-KSHV VCA IgG levels (Fig 7B left panel).

**Fig 7:**
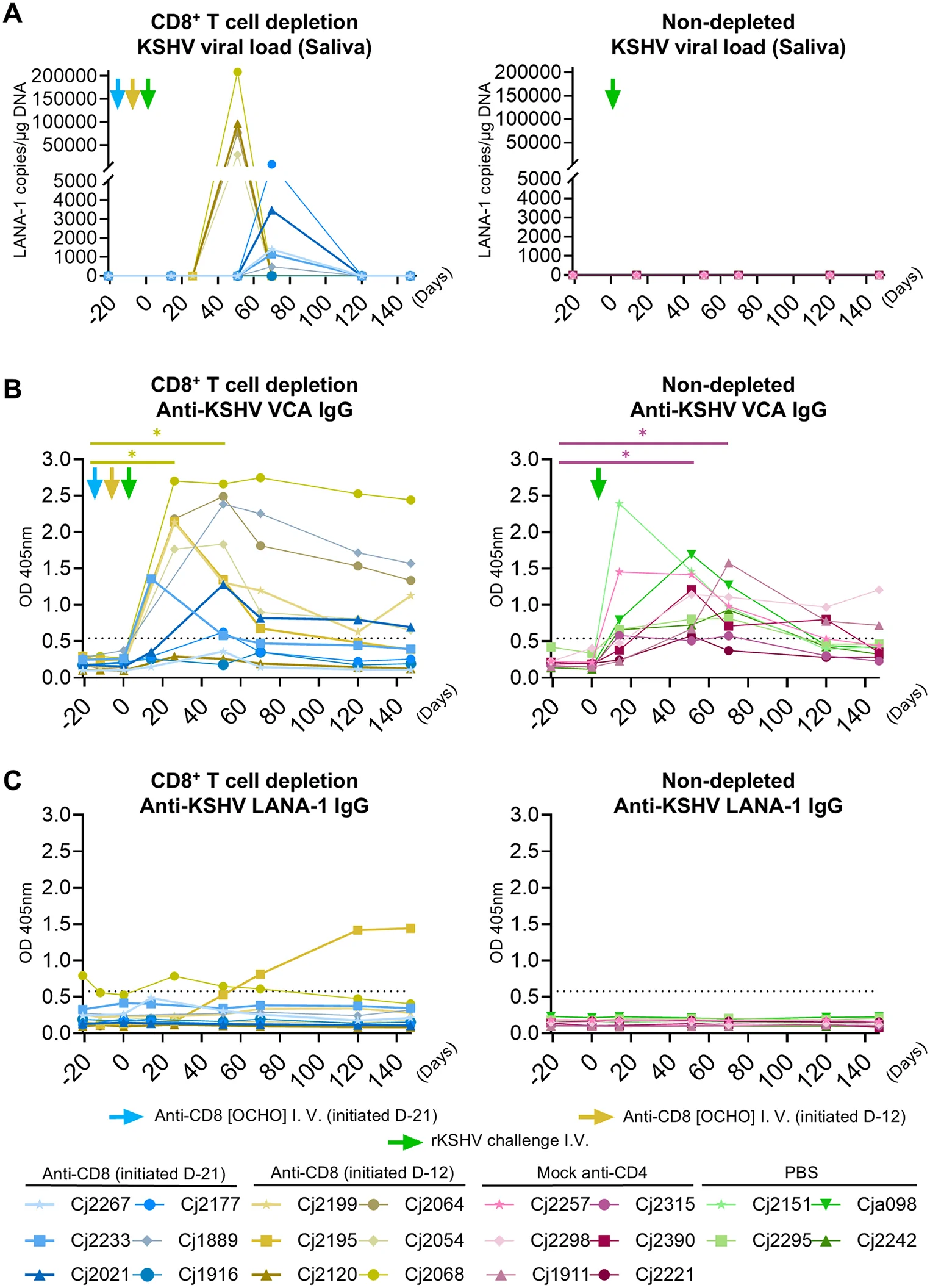
KSHV viral load in saliva and anti-KSHV VCA and anti-KSHV LANA-1 IgG production in anti-CD8 [OCHO]-treated marmosets and non-depleted groups upon intravenous (I.V.) rKSHV challenge. **(A)** Quantification of LANA-1 gene DNA copy numbers per µg of DNA (i.e., KSHV viral load) isolated from saliva in rKSHV-challenged marmosets (described in Fig 6, n = 22) over time using qPCR. Each datapoint represents the mean of four technical replicates. **(B)** Measurement of anti-KSHV VCA IgG levels in plasma diluted 1/25 from challenged marmosets (described in Fig 6) over time using ELISA. Each datapoint represents the mean of two technical replicates. Cut-off value was calculated as described in Materials and methods and is represented using dotted lines. Statistical significance was assessed using Mixed-effect analysis with Geisser-Greenhouse correction to compare treatment effect across timepoints, however no significant differences were detected (p = 0.0638). Next, repeated measures one-way ANOVA with Geisser-Greenhouse correction followed by Dunnett’s multiple comparison test was used to identify differences between timepoints within a group. * = p < 0.05 between indicated timepoints. Matching colors are used to denotate differences within a single group. **(C)** Measurement of anti-KSHV LANA-1 IgG levels in plasma diluted 1/25 from challenged marmosets (described in Fig 6) over time using ELISA. Each datapoint represents the mean of two technical replicates. Cut-off value was calculated as described in Materials and methods and is represented using dotted lines.

We quantified KSHV viral load in tissue samples collected at study endpoint (D147), indicative of successful establishment of persistent infection across experimental groups. Viral loads were quantifiable in spleens from all marmosets except Cj2064 and Cj2195, both belonging to the anti-CD8 (initiated D-12) treatment group (Fig 8A); however, this observation should be interpreted with caution as all tissue assessments could present a risk of false negatives, due to possible localized infection in tissue areas that might not have been processed. Indeed, Cj2195 displayed LANA-1 seroconversion by the study endpoint, suggesting infection was established despite not showing DNA viral loads in the spleen. We also detected viral load in various lymphatic tissues of all infected marmosets except Cj2064 (Fig 8A and S10). Moreover, 19/22 infected marmosets across all experimental groups showed quantifiable viral loads in the liver, and 14/22 marmosets showed at least traces of KSHV DNA in lung tissues (Fig 8B and S10). The presence of KSHV viral load in non-lymphoid tissues (kidney, colon, tongue, and heart) was inconsistent (Fig 8B and S10). Importantly, we did not detect KSHV DNA in any tissue collected at SNPRC from four unrelated, uninfected control marmosets, confirming the specificity of the assay and verifying the infection status of the cohort (S10 Fig). In addition, LANA-1 expression in spleen was assessed by KSHV LANA-1 IHC. In CD8⁺ T cell-depleted groups, one marmoset from anti-CD8 (initiated D-12) was classified as LANA-1 positive frequent (IHC 3), two as LANA-1 positive (IHC 2), and one as LANA-1 inconclusive (IHC 1); while in the anti-CD8 (initiated D-21) group one marmoset was classified as IHC 3, one as IHC 2 and two as IHC 1 (S11A Fig and S12 Fig). In the non-depleted groups, one marmoset from the mock anti-CD4 group was classified as IHC 3 and three as IHC 2, while in the PBS group two marmosets were classified as IHC 2 and one as IHC 1 (S11A Fig and S12 Fig). As shown in S11A Fig, all samples that were found negative by qPCR were classified as negative by IHC (IHC 0), however among the positives we found no correlation between the quantified KSHV DNA copies in the sample and the IHC score (Spearman test: r = 0.2097, p = 0.3039) suggesting that qPCR is more sensitive for the detection of low levels of KSHV infection (S11B Fig). This observation is aligned with previous reports from our group where we reported EBER-ish staining can fail to detect EBV positive samples with a viral load under 6 × 10^2^ EBV copies/ug DNA as detected by qPCR [55].

**Fig 8:**
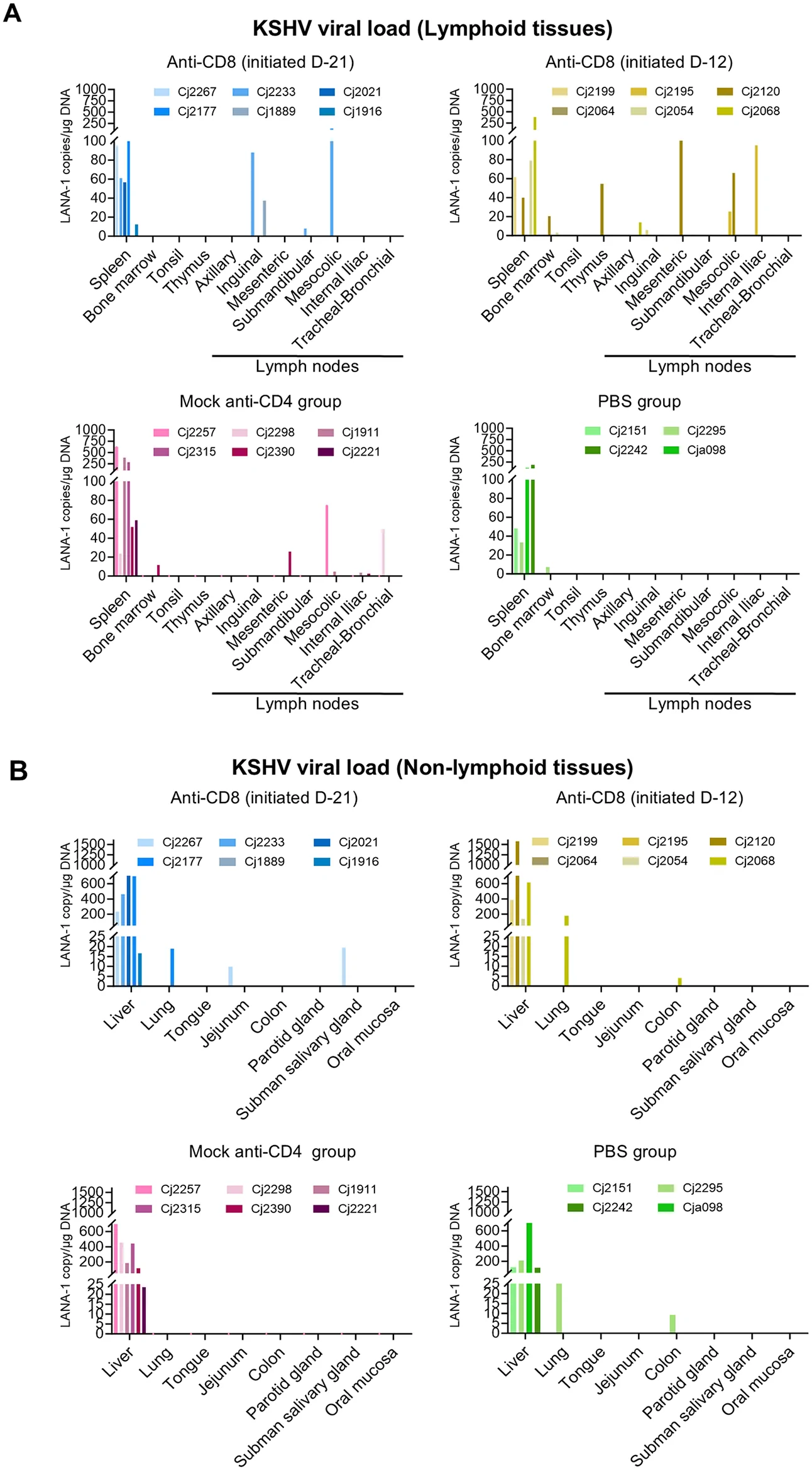
KSHV viral load in lymphoid and non-lymphoid tissues in anti-CD8 [OCHO]-treated marmosets and non-depleted groups upon rKSHV challenge. **(A-B)** Quantification of LANA-1 gene DNA copy numbers per µg of DNA (i.e., KSHV viral load) isolated from various lymphoid **(A)** and non-lymphoid **(B)** tissues of marmosets at euthanasia (described in Fig 6) using qPCR. Each bar represents the mean of two technical replicates.

We performed health assessments throughout both chimeric mAb studies, which revealed no adverse effects associated with either antibody, demonstrating a superior safety profile as compared to prior tacrolimus regimens. We identified suspicious skin lesions in three marmosets (Cj2267, anti-CD8 (initiated D-21), D86; Cj2054, anti-CD8 (initiated D-12), D49; and Cj2315, mock anti-CD4, D91). We analyzed skin biopsies from the lesion sites and distal unaffected skin using H&E histology and KSHV LANA-1 IHC (S9A–C Fig), but we could not confirm any lesion as a KS-like skin lesion.

Overall, our results demonstrate that persistent KSHV infection can be established in common marmosets under both immunocompetent and immunosuppressed conditions, as evidenced by the non-depleted vs. depleted marmoset groups, respectively. Importantly, CD8⁺ T cell depletion via anti-CD8 [OCHO] enabled the first demonstration of KSHV salivary shedding in marmosets, a critical hallmark of KSHV infection in humans [56]. These findings position anti-CD4 [TAWA] and anti-CD8 [OCHO] as valuable tools for immune modulation in future marmoset studies of KSHV viral pathogenesis.

## Discussion

Building on the foundational studies [31,34] that demonstrated the susceptibility of the common marmoset to KSHV, we sought to further validate this model in both immunocompetent and immunosuppressed settings across several independent experiments. The original pioneering study showed that tacrolimus administration before KSHV challenge could impair the establishment of infection, as tacrolimus inhibition of calcineurin can interfere with the KSHV life cycle [31,33]; we extended this work by testing post‑challenge administration of intravenous tacrolimus, which led to detection of KSHV in PBMCs and several tissues. However, the daily intravenous tacrolimus regimen proved to be poorly tolerated. We next administered oral tacrolimus as an alternative but could not achieve therapeutic levels of tacrolimus in the treated marmosets. In addition, because tacrolimus exerts its immunosuppressive effects by inhibiting T cell activation rather than inducing direct cellular depletion [57,58], quantifying the immunosuppressive efficacy represented a challenge.

To overcome these constraints, we aimed to establish a targeted, mAb-mediated depletion, which is a well-established immunosuppressive strategy previously used in NHP models [37,59,60]. Using two existing murine mAbs that recognize marmoset CD4 and CD8, mouse anti-rhesus CD4 [19Thy5D7] [60] and mouse anti-marmoset CD8 [6F10] [37], we generated the marmoset-chimerized mAbs, anti-CD4 [TAWA] and anti-CD8 [OCHO], respectively. A chimeric mAb approach enables repeated *in vivo* administration with minimal generation of anti-mouse antibodies, which in humans and other NHPs can lead to acceleration of mAb clearance, allergic and hypersensitivity reactions, or reduction of mAb efficacy [43,61]. Following chimerization, both antibodies retained their *ex vivo* binding to marmoset CD4^+^ and CD8^+^ T cells. Furthermore, we validated a previously reported flow cytometry mAb panel as compatible with our chimeric mAbs and capable of precise tracking of T cell, B cell, monocyte, and NK cell populations in marmosets [46], providing an essential resource with which to monitor the depth and kinetics of immunosuppression in our chimeric mAb-treated marmoset model.

In our initial chimeric mAb-mediated depletion experiment, a cocktail of the chimeric mAbs rapidly reduced the CD4^+^ T cell population within hours of administration, and maintained this reduction through D19. Although CD8^+^ T cells appeared to be rapidly and persistently depleted, this analysis was performed prior to validating the compatible flow cytometry mAb panel, likely resulting in an overestimation due to epitope competition between the injected OCHO mAb, still systemically available as shown by ELISA, and its parental mAb (6F10) used for flow cytometry staining. Due to the small body size of marmosets (350–450 g), blood collection is limited to approximately 1% of body weight per month [30,62], restricting the number of PBMCs available at each bleeding point and preventing us from repeating this assessment using the validated mAb panel. To rigorously confirm the immunosuppressive activity of anti-CD4 [TAWA] and anti-CD8 [OCHO], we therefore replicated the depletion regimen in an independent marmoset cohort. In this additional cohort, both CD4⁺ and CD8⁺ T cells were efficiently depleted soon after cocktail administration and remained markedly reduced until the timepoint at which KSHV would be administered in a KSHV challenge experiment. CD4⁺ T cell kinetics closely mirrored those in the first chimeric mAb-mediated depletion experiment, while CD8⁺ T cell kinetics were consistent with prior reports using the parental anti-CD8 [6F10] mAb in marmosets [37], supporting the depleting capacity of anti-CD8 [OCHO] was maintained after chimerization and strongly suggesting effective CD8⁺ depletion in the rKSHV‑challenged cohort. Furthermore, we observed no significant effects of the chimeric mAbs on B cells, NK cells, or monocytes, underscoring their specificity for T cell subsets. Of note, our analyses demonstrated that it is crucial to assess both cell population frequencies and derived absolute blood cell counts to accurately interpret the depth of immunosuppression, in line with best practices in longitudinal immunoprofiling clinical studies [63,64] and should be considered in follow-up marmoset studies.

In our second chimeric mAb-mediated depletion experiment, which was followed by KSHV challenge, we sought to more precisely define the kinetics and contribution of each mAb to KSHV infection outcomes by administering anti-CD4 [TAWA] and anti-CD8 [OCHO] either individually or in sequence rather than exclusively as a cocktail. However, loss of functional activity of stored anti-CD4 [TAWA] during this phase prevented a complete evaluation of CD4⁺ T cell depletion, highlighting the need for additional work to rigorously characterize the long‑term stability and storage requirements of this reagent, and periodically confirming reagent functionality throughout the length of the study. In contrast, stored anti-CD8 [OCHO] retained full activity and consistently mediated robust CD8⁺ T cell depletion within 7–17 days after the final dose. Taking the findings from all the depletion experiments together, we demonstrated that both of our chimeric mAbs can effectively and selectively deplete the targeted T cell populations in marmosets, but further investigation is required to dissect the depletion kinetics of each antibody when given individually or in combination, and to determine which regimen (i.e., cocktail vs. sequential administration) provides the most suitable regimen for future KSHV pathogenesis and intervention studies.

In KSHV challenge experiments, we observed significant KSHV viral loads in both lymphoid and non-lymphoid tissues in challenged marmosets regardless of immunosuppression status. It is unclear why one challenged marmoset, belonging to the anti-CD8 (initiated D-12) group, did not display infection in any tissue. Across all experiments, tissue tropism closely paralleled the original reports of KSHV infection in marmosets [31,34], with the exception of the parotid gland and the oral mucosa, in which we report the detection of KSHV traces for the first time, in contrast to the previous study that reported them as KSHV-negative [31]. This discrepancy likely reflects higher assay sensitivity of our qPCR assay compared with conventional PCR used in the previous study, combined with the low overall incidence of infection in these sites (4/30, or ≈13%) in our study. We additionally identified KSHV infection in the internal iliac, mesocolic, and tracheobronchial lymph nodes, which had not been previously assessed. Overall, we found the spleen and liver to be the most consistently infected tissues, with frequent detection of traces of KSHV DNA in the lung, in line with earlier reports [31,34]. We only observed PBMC viremia in marmosets treated with the chimeric mAb cocktail, and it remains unclear whether this is a result of the combined CD4/CD8 immunosuppression, or the result of using a different KSHV strain for challenge in the cocktail (rKSHV.219) vs. the individual administration (rKSHV BAC16) experiments. Of note, in prior studies, PBMC viremia was frequently detected in rKSHV.219-challenged marmosets [31], whereas only inconsistent traces of KSHV DNA were detected in rKSHV BAC16-challenged marmosets [34]. Although PBMC viremia was generally low in our model, this feature closely mirrors the virologic profile of KSHV‑infected humans, in whom viremia can remain low or only intermittently detectable even in patients with overt disease [65,66].

A key advancement of our work is the first demonstration of KSHV salivary shedding in marmosets, particularly in those under mAb-mediated immunosuppression. By integrating our assessment of KSHV viral load in saliva (i.e., salivary shedding) with our longitudinal measurement of anti‑KSHV VCA blood IgG (i.e., seroconversion), our results suggest that immunocompetent marmosets may rapidly mount an effective anti-viral response that restricts KSHV to either latent reservoirs or low-level replication, and prevents sustained salivary shedding; however, our observations do not exclude the possibility of viral shedding occurring in these animals between sampling timepoints, or that could have occurred beyond our experimental timeline. In contrast, marmosets immunosuppressed via the chimeric mAb cocktail or anti-CD8 [OCHO] alone showed clear evidence of heightened lytic replication in the form of salivary shedding. Cocktail‑treated marmosets exhibited an early peak of salivary shedding after infection in the absence of detectable seroconversion, consistent with a loss of CD4⁺ T cells, which are required to coordinate a robust, antigen-specific antibody response [67]. Although this pattern could reflect a primary infection alone, one of the four marmosets in this group developed a second and higher salivary shedding peak approximately two months post‑challenge, which came after a period of undetectable viral load in the saliva, but this time paired with production of anti-KSHV VCA IgG as the CD4⁺ T cell compartment recovered. These findings strongly suggest the marmoset model can sustain KSHV lytic replication, and our observations were confirmed by our final experiment in which anti-CD8 [OCHO] only-treated marmosets experienced distinct salivary shedding episodes 2–3 months after KSHV challenge, typically accompanied by a boost of anti-KSHV VCA IgG levels. Interestingly, the magnitude and timing of the salivary shedding appeared closely linked to CD8⁺ T cell depletion kinetics, as marmosets in which CD8⁺ T cell depletion was initiated earlier (D‑21) displayed later onset and lower levels of shedding than those in in which CD8⁺ T cell depletion was initiated closer to the challenge timepoint (D‑12). Considering that KSHV viral reactivation and disease have been linked to the absence of a robust specific CD8^+^ T cell immune response in humans [68], our data suggests that prolonged CD8⁺ T cell depletion after infection, as in the anti‑CD8 (initiated D‑12) group, predisposes marmosets to more pronounced salivary shedding, likely due to an impaired capacity to mount an effective cytotoxic immune response during primary infection. Conversely, the relatively rapid resolution of these acute shedding episodes is consistent with restoration of the T cell compartments by the time of KSHV reactivation, in agreement with observations from *in vitro*, *ex vivo*, and clinical studies [69–71].

Although our study did not focus on elucidating the kinetics of the viral life cycle in this model, our data suggests that KSHV can undergo latency in the marmoset, as evidenced by our detection of LANA-1 expression in infected spleens by KSHV LANA-1 IHC and the presence of anti-KSHV LANA-1 IgG by ELISA. Of note, we observed that LANA-1 seroconversion was not consistent with LANA-1 expression detected by IHC. The most common scenario involved LANA-1 expression in spleen in the absence of seroconversion, which could reflect natural infection in humans in which seroconversion may be delayed for even more than a year after detection of KSHV infection [72,73]. Thus, it is possible that due to the relatively short length of our study we may have missed the detection of delayed LANA-1 seroconversion. In contrast, the marmoset that displayed LANA-1 seroconversion without detection of KSHV LANA-1 DNA or protein in the spleen, could be related to establishment of latency on a different tissue or untested spleen sections. Future studies should aim to fully dissect the KSHV life cycle in this model.

Regarding KS pathogenesis in marmosets, although we observed suspicious skin lesions in four marmosets across our experiments, none met traditional diagnostic criteria for KS. Given the prior report of KS susceptibility in marmosets [31], our findings suggest that prolonged immunosuppression regimens may be required to recapitulate KS‑permissive conditions observed in humans, such as HIV/AIDS or post‑transplant immunosuppression regimens [7–9]. Therefore, future work using continuous or repeated dosing with anti-CD4 [TAWA] and/or anti-CD8 [OCHO] to sustain long‑term T cell depletion will be important to determine whether the marmoset model can reliably generate KS‑like lesions and to define the immune thresholds that govern progression from latent KSHV infection to tumorigenesis.

Beyond elucidating the specific immune pathways that control KSHV infection and pathogenesis, the marmoset model and our marmoset-chimerized mAbs offer a biologically relevant platform for preclinical testing of KSHV‑directed antivirals, immunotherapies, and vaccines. As common marmosets also support research on the KSHV-related herpesvirus Epstein-Barr virus [74] and diverse infectious and non‑infectious conditions [30,75–77] including tularemia, melioidosis, hepatitis C, hemorrhagic fevers, encephalitis, severe acute respiratory syndrome, and aging, these tools are poised to have broad translational utility across multiple areas of biomedical research.

Future studies using this model will benefit from additional lessons learned from our work. In this study, individual challenge experiments were performed sequentially over a long period of time (~6 years). Due to intrinsic challenges associated with virus production, characterization, and titration, different viral preparations and titration methods were used across experiments. While this variability allowed us to confirm that the marmoset is susceptible to KSHV infection under a variety of challenge conditions, future studies should ideally utilize a single well-characterized viral stock for all animals within a given study cohort to minimize experimental variability and enable more direct comparisons across groups. For detection of viral DNA in tissue, the potential for false-negative results due to sampling bias can be reduced by analyzing multiple regions within a tissue or by examining larger tissue sections. Similarly, for detection of viral DNA in blood, increasing the volume of blood processed may improve sensitivity and reduce the likelihood of false-negative results. Given that multiple assays in this type of work require blood (qPCR, flow cytometry, ELISA), longitudinal sampling frequency should be carefully designed to prioritize an accurate measurement of the target variables at key timepoints while meeting allowed marmoset blood collection limits. Although beyond the scope of the current study, future investigations should also identify the specific cell types targeted by KSHV in marmosets, and the associated cellular immune responses, to fully characterize this model. Finally, we acknowledge that the IV route of infection does not fully recapitulate the natural routes of KSHV transmission in humans. Hence, future studies should focus on characterizing physiological relevant routes of infection, including the potential for oral transmission, particularly given that infected animals in this study demonstrated evidence of viral shedding in the oral cavity.

In conclusion, our study further establishes a transformative marmoset model for KSHV infection with broad applications beyond KSHV. We have: (1) confirmed the susceptibility of common marmosets to KSHV infection in both immunocompetent and immunosuppressive settings across multiple independent experiments; (2) demonstrated a strong similarity between KSHV pathogenic features in marmosets and humans; and (3) generated and functionally validated two marmoset-chimerized mAbs, anti-CD4 [TAWA] and anti-CD8 [OCHO], as novel immunosuppressive tools that can effectively and safely deplete CD4^+^ and CD8^+^ T cells in marmosets. Together, these advancements are poised to accelerate research on KSHV and other human pathogens in this versatile NHP model, and to guide development of improved therapeutic and prophylactic strategies for human disease.

## Materials and methods

### Ethics statement

All animal studies were conducted in strict accordance with the standards set forth by the American Association for Accreditation of Laboratory Animal Care (AAALAC), and with approved Institutional Animal Care and Use Committee (IACUC) protocols at Wisconsin National Primate Research Center/WNPRC (IACUC #G00741, #G005876, #G006616), Southwest National Primate Research Center/SNPRC (#1880 CJ 3) and the Beckman Research Institute of City of Hope (IACUC #16002, #24027). As negative controls for KSHV infection, additional PBMCs, and lymphoid and non-lymphoid tissues were obtained from four rKSHV-non-infected marmosets that were unrelated to our study and independently provided by the SNPRC. As requested by SNPRC, original SNPRC animal IDs were replaced with arbitrary IDs for confidentiality purposes. As positive control during flow cytometry antibody panel validation, human PBMCs isolated from residual blood cones of de-identified platelet donors at the City of Hope Michael Amini Transfusion Medicine Center were used, as approved by the Institutional Review Board (IRB) at City of Hope (IRB #16011).

### Cells

Human embryonic kidney cells (HEK-293; CRL-1573) were obtained from American Type Culture Collection (ATCC) and maintained in complete DMEM: Dulbecco’s modified eagle medium (DMEM; 10–013-CM Corning) supplemented with 10% fetal bovine serum (FBS, 25–550 GenClone), 1% L-glutamine (25030081, Gibco) and 2% Penicillin-streptomycin (15140122, Gibco).

Hybridoma cells producing mouse 6F10 anti-marmoset CD8 [78] were provided by Dr. Mamoru Ito (Central Institute for Experimental Animals, Japan), and maintained in Roswell Park Memorial Institute medium (RPMI 1640, Cytiva) supplemented with 10% FBS, 1% L-glutamine and 2% Penicillin-streptomycin.

The SLK cell line containing stable Tet-On doxycycline-inducible RTA (iSLK) under puromycin and neomycin (G418 sulfate) resistance genes [79,80] was provided by Dr. Jung and was maintained in iSLK growth media: complete DMEM supplemented with 1 µg/mL puromycin (P600-100, Gold Biotechnology) and 250 µg/mL neomycin (G418 sulfate, N6386-100G, Sigma-Aldrich).

Two different iSLK cell lines containing the recombinant KSHV viruses rKSHV.219 [80] and rKSHV BAC16 [79], both expressing enhanced green fluorescent protein (eGFP), were kindly provided by Dr. Dirk Dittmer (University of North Carolina at Chapel Hill, North Carolina) and Dr. Jung, respectively. The cell lines were maintained in either rKSHV.219 or rKSHV BAC16 selection media: for rKSHV.219, complete DMEM was supplemented with 10 µg/mL puromycin, 250 µg/mL neomycin, and 450 µg/mL hygromycin B (H-270–1, GoldBio); for rKSHV BAC16, complete DMEM was supplemented with 1 µg/mL puromycin, 250 µg/mL neomycin, and 1,000 µg/mL hygromycin B. All cells were cultured at 37 °C in a humidified incubator with 5% CO_2_.

### Virus production and titration

KSHV virus was produced using iSLK cells containing either rKSHV.219 or rKSHV BAC16, following a protocol previously described [79,80]. Briefly, infected iSLK cells were cultured in their respective selection media and expanded to 100 15-cm dishes. Next, KSHV lytic replication was induced for 24 h by exchanging the media to complete DMEM supplemented with 2 µg/mL doxycycline (D5207, Millipore Sigma) and 1.5 mM sodium butyrate (NaB; FT-FSIJK479725, Frontier Scientific). Cells were then maintained in complete DMEM for an additional four days. The cell-free supernatant was harvested and purified by a two-step centrifugation. Cell debris were first removed by centrifugation at 3,283 g for 20 min (Allegra X-14R Refrigerated Centrifuge, Beckman Coulter) followed by ultracentrifugation at 38,200 g for 90 min (Optima XE-90, Beckman Coulter) to pellet the virions. The resulting virus was resuspended in Opti-MEM (31985062, Gibco) and stored at -80 °C until use.

Obtained KSHV batches were titrated in iSLK cells to determine infectious titers. iSLK cells were seeded in quadruplicate at a density of 2 × 10^5^ cells per well in a 48-well plate. After 24 h, the cells were washed once with Dulbecco’s phosphate-buffered saline, 1X without calcium and magnesium (DPBS; 21–031-CV, Corning) and inoculated with varying amounts of purified virus in 100 µL of Opti-MEM for 1 h at 37 °C. The cells were then washed three times with PBS and cultured in iSLK growth media for an additional 24 h. Harvesting of the cells was subsequently carried out by trypsinization, followed by the addition of an equal volume of complete DMEM to inhibit trypsin activity. Subsequently, the cells were washed with DPBS and fixed in 2% paraformaldehyde (PFA; 157-4-100, Electron Microscopy Sciences) in PBS. The fixed cells were subjected to flow cytometry analysis. Alternatively, virus titration was performed in HEK-293 cells. Briefly, cells were seeded in triplicate at a density of 5 × 10^4^ per well in a 48-well plate. After 24 h, media was removed and varying amounts of purified virus in 100 µL of Opti-MEM were added and incubated for 1 h at 37 °C. Following, 400 µL of complete DMEM was added to the cells, then cells were incubated for an additional 24 h after which they were harvested and fixed in 2% PFA for flow cytometry analysis. In both methods, the number of infected cells (eGFP^+^) was quantified using the BD Accuri C6 flow cytometer and the obtained data was analyzed using FlowJo software (BD).

### Common marmoset housing and protocols

Thirty female and male common marmosets (*Callithrix jacchus*, Cj; see S10 Fig) were housed in specific-pathogen-free facilities at WNPRC. Additional nine marmosets were housed at SNPRC under an unrelated project. To prevent potential background cross-reactivity with KSHV, all marmosets included in this study were confirmed to be marmoset lymphocryptovirus (Callitrichine gamma-herpesvirus 3, CalHV-3)-free by qPCR performed by SNPRC. The qPCR was performed as previously described [81], using CalHV-3-specific primers (S2 Table). Marmosets were housed either paired at WNPRC or singly at SNPRC and assigned to four different experiments as described below.

*Experiment 1*: Two common marmosets (Fig 1A) were intravenously challenged with two doses of rKSHV.219 virus (1 × 10^7^ iSLK IU) on days (D)0 and D88. To induce immunosuppression, tacrolimus was administered by intravenous at 100 μg/kg/day from D218 daily until the end of the study (D304). Blood samples were collected periodically for isolation of PBMCs and plasma. Sera was also obtained at the endpoint.

*Experiment 2*: Two common marmosets (Fig 1G) were intravenously challenged with a single dose of the rKSHV.219 (2 × 10⁷ HEK-293 IU) on D0. Tacrolimus was then orally administered with food at a dose of 0.3 mg/kg/day starting on D87, with the dose subsequently increased to 0.4 mg/kg/day from D192, and to 0.8 mg/kg/day from D213, until protocol completion (D227). Blood samples were collected periodically for isolation of PBMCs and plasma. Sera was also obtained at the endpoint.

*Experiment 3*: To assess the depletion efficacy of our two newly described marmoset-chimerized anti-CD4 [TAWA] and anti-CD8 [OCHO] mAbs, four common marmosets (Fig 3A) received three intravenous doses of a cocktail of these mAbs (10 mg each/kg body weight) 14 (D-14), 9 (D-9) and 7 (D-7) days before the viral challenge. Following, the marmosets were intravenously challenged (D0) with rKSHV.219 virus (5 × 10^5^ HEK-293 IU) via injection. PBMCs, serum, plasma and saliva were collected periodically until the end of the study (D147). To confirm the efficacy of the immunosuppression regimen used in this study, the additional nine marmosets maintained in SNPRC facilities and allocated to a non-related project from our research group were used. Four marmosets were treated using the anti-CD4 [TAWA] and anti-CD8 [OCHO] mAbs cocktail in the same dose and following a similar immunization scheme as described above (Fig 4A). In parallel, an immunocompetent group (n = 5) received PBS as placebo. Marmosets were bled for PBMC isolation before starting the immunosuppression regimen (D0), and one week (D14), three weeks (D28), and almost two months (D56) after post-final cocktail dose. These marmosets were not challenged, and no other samples were collected for the purpose of this study.

*Experiment 4*: To further assess the depletion kinetics of the marmoset-chimerized anti-CD4 [TAWA] and anti-CD8 [OCHO] mAbs, 22 common marmosets (S6A Fig) were assigned to four treatment groups: anti-CD4 (anti-CD4 [TAWA] administration only, later renamed ‘mock anti-CD4’), anti-CD8 (anti-CD8 [OCHO] administration only, later renamed ‘anti‑CD8 (initiated D‑21)’), anti-CD4 f/b anti-CD8 (anti-CD4 [TAWA] administration followed by anti-CD8 [OCHO], later renamed ‘anti‑CD8 (initiated D‑12)’), and PBS (non-depleted control group). All treated groups received three intravenous doses of 10 mg/kg body weight of the corresponding mAb (anti-CD4 [TAWA] or anti-CD8 [OCHO]) on D-21, D-19, and D-17, while the control group received an equivalent volume of PBS. The anti-CD4 f/b anti-CD8 group, received three doses of anti-CD8 [OCHO] after the completion of anti-CD4 [TAWA] administration on D-12, D-10, and D-7. All marmosets were intravenously challenged with rKSHV BAC16 (5 × 10^5^ HEK-293 IU) on D0. PBMCs, serum, plasma and saliva were collected periodically until the end of the study (D147).

In all studies, marmosets were followed for behavioral, physical and physiological changes, such as loss of appetite, weight loss, anemia, and other clinical signs. If marmosets developed skin lesions suspicious for KS over the course of the study, biopsies from the lesion and distal (uninvolved) skin were collected and formalin-fixed and paraffin-embedded (FFPE) for hematoxylin and eosin (H&E) staining and immunohistochemistry (IHC) analysis. At the study endpoint, marmosets were euthanized, and various lymphoid and non-lymphoid tissues were harvested. WNPRC provided these tissues in two formats: either preserved and frozen in RNAlater (AM7024, Invitrogen) for downstream DNA isolation and qPCR analysis, or FFPE for histological and immunohistochemical evaluation. When available, isolated cells from lymphoid tissues were also provided frozen in complete RPMI medium containing DMSO. Samples preserved in RNAlater were stored at –80 °C until analysis, isolated cells were maintained in liquid nitrogen (LN_2_), and FFPE samples were stored at temperature (RT).

Tacrolimus suspensions used in experiments 1 and 2 were prepared by compounding the granule content in 1 mg tacrolimus capsules (PROGRAF, Astellas Pharma US). The generation and characterization of the mAbs used in experiments 3 and 4 are described below in the section titled *“Generation of marmoset-chimerized anti-marmoset CD4 and anti-marmoset CD8”*.

### Generation of marmoset-chimerized anti-marmoset CD4 and anti-marmoset CD8 mAbs

To generate mAbs to be used in the targeted depletion of marmoset CD4^+^ and CD8^+^ cell populations, the capacity of different mAbs to recognize these populations was studied *ex vivo* using flow cytometry. Marmoset PBMCs obtained from Cj074 (Experiment 1) on D127 and D142 were pooled and single-stained with 10 µg/mL of individual antibody in PBS of the following primary antibodies: mouse anti-human CD4 [OKT4] (PR-4000, RRID: AB_2819255, NHPRR), rhesusized anti-human CD4 [CD4R1]-FITC (PR-0040, RRID: AB_2895641, NHPRR), mouse anti-rhesus CD4 [19Thy5D7] (PR-4190, RRID: AB_2819279, NHPRR), mouse anti-rhesus CD8alpha [7Pt-3F9] (PR-8130, RRID: AB_2819280, NHPRR), rhesusized anti-rhesus CD8beta [CD8b255R1]-FITC (PR-0095, RRID: AB_2941337, NHPRR), rhesusized anti-rhesus CD8alpha [M-T807R1]-FITC (PR-0099, RRID: AB_2941341, NHPRR), and mouse anti-marmoset CD8 [6F10]-PE (250304, Biolegend). When unconjugated primary antibodies were used, samples were subsequently incubated in a 1:500 dilution of AF488-conjugated goat anti-mouse IgG (Poly4053, Biolegend) in PBS. Cells were washed between each step with 5 mL PBS and pelleted at 205 g for 5 min (Allegra X-14R Refrigerated Centrifuge, Beckman Coulter). Finally, cells were fixed in 1% PFA and analyzed using a BD Accuri C6 flow cytometer.

To avoid the induction of marmoset anti-mouse antibodies, the selected antibodies, mouse anti-rhesus CD4 [19Thy5D7] and mouse anti-marmoset CD8 [6F10], were first sequenced by NHPRR and City of Hope, respectively. The identified variable regions were then cloned into the marmoset IgG backbone to recombinantly express marmoset-chimerized versions of these two antibodies, which were subsequently renamed anti-CD4 [TAWA] and anti-CD8 [OCHO]. The generation and production of TAWA and OCHO were performed by the NHPRR. To confirm the activity retention of the marmoset-chimerized mAbs, the mAbs were tested by flow cytometry using PBMCs obtained from Cj074 at D112 following the protocol described above. Staining using the parent clones was performed as positive control.

### Anti-CD4 [TAWA]- and anti-CD8 [OCHO]-specific ELISA

To optimize the detection of anti-CD4 [TAWA] and anti-CD8 [OCHO] antibodies by ELISA, 96 well MaxiSorp plates (439454, ThermoFisher Scientific) were coated overnight at 4°C with 100 µL per well of recombinant His-tagged rhesus CD4 (90274-C08H, SinoBiological) or recombinant His-tagged marmoset CD8 (custom-made by Genscript) diluted in carbonate buffer at 2 µg/mL. Plates were subsequently washed three times with 300 µL of washing buffer (0.1% Tween-20 in PBS) and blocked with 200 µL of 5% skim milk (Good & Gather) in washing buffer. After a 1 h incubation, plates were washed as described and 100 µL of serially diluted anti-CD4 [TAWA] or anti-CD8 [OCHO] were respectively added and incubated for an additional 2 h at RT. Next, plates were washed and incubated for 1 h with 100 µL of goat anti-human IgG (H + L)-HRP (C31410, Thermofisher Scientific) diluted 1:2000 in 1% skim milk in PBS. After a final wash, plates were developed using 100 µL ABST peroxidase substrate solution (5120–0035, LGC SeraCare), followed by the addition of 100 µL stop solution (5150-0017, LGC SeraCare) after 20 min. Optical density (OD) values were measured at 405 nm using a FilterMax F3 (Molecular Device).

For the quantification of systemic levels of anti-CD4 [TAWA] and anti-CD8 [OCHO] in treated marmosets, marmosets’ plasma obtained at different timepoints were evaluated by ELISA at different dilutions as described above. The resulting OD values were extrapolated to a standard curve of the targeted mAb included in the same ELISA plate. In cases where the parental clones were included in the same ELISA, the secondary antibody was replaced by goat anti-Mouse IgG (H + L)-HRP (1706516, Bio-Rad).

### Flow cytometry analysis

A previously reported antibody panel (S1 Table) for the characterization of different marmoset PBMCs populations by flow cytometry was assessed [45–47]. Considering that all the selected antibodies are not commercially validated for their use in marmoset, with the exception of anti-marmoset CD45 [6C9]-PE (250204, BioLegend), the panel was tested using archived marmoset PBMCs and splenocytes obtained from SNPRC. As positive control, de-identified human PBMCs were used.

First, the capacity of the selected anti-CD4 [L200]-PerCP-Cy5.5 and anti-CD8 [HIT8a]-BUV805 mAbs (S1 Table) to detect CD4 and CD8 T cells from marmosets pre-treated with anti-CD4 [TAWA] and anti-CD8 [OCHO] was assessed by a competition assay. Briefly, marmoset splenocytes were thawed and washed in RPMI 1640 medium supplemented with 10% FBS and 2mM L-Glutamine. Cell count and viability were assessed by trypan blue staining (15250061, Gibco) using a TC20 Automated Cell Counter (Bio-Rad). A total of 3 × 10^5^ PBMCs per tube were incubated with the live/dead staining reagent Zombie NIR dye (423106, BioLegend) diluted 1:100 in PBS containing 0.5% bovine serum albumin (BSA, A9647-500g, Sigma-Aldrich) and 2 mM EDTA (staining buffer) for 15 min at RT in the dark. Cells were then washed once with staining buffer at 300 g for 5 min at 4 °C and incubated for 30 min at 4 °C with 100 µL of antibody mixture using the following conditions: single staining (anti-CD4 or anti-CD8 only), simultaneous co-staining(both anti-CD4, ‘TAWA + L200’; or both anti-CD8,‘OCHO + HIT8a’), sequential co-staining with chimeric antibodies followed by the flow cytometry mAbs (‘TAWA → L200’ or ‘OCHO → HIT8a’), or vice versa (‘L200 → TAWA’ or ‘HIT8a → OCHO’). In this experiment, conjugated version of the marmoset-chimerized mAbs, anti-CD4 [TAWA]-AF647 and anti-CD8 [OCHO]-AF488, were used. Additionally, all staining conditions included anti-CD3 [SP34-2] for lymphocytic population detection. After staining, cells were washed twice with staining buffer, followed by fixing with 1% PFA. Samples were analyzed via flow cytometry using NovoCyte Quanteon Flow Cytometer Systems 4 Lasers (Agilent). The data obtained was analyzed using FlowJo Software and results were plotted using GraphPad Prism software (Dotmatics).

To complete the panel validation, marmoset PBMCs, splenocytes, and human PBMCs were thawed, counted, and stained with the live/dead reagent as described before. Following, cells were washed once with staining buffer and incubated with a 100 µL of antibody mixture (5 µL anti-CD45-PE, 2.5 µL anti-CD3-Pacific Blue, 20 µL anti-CD4-PerCP-Cy5.5, 3 µL anti-CD8-BUV805, 5 µL anti-CD20-FITC, 2.5 µL anti-CD14-PE-Cy7, 5 µL anti-CD16-BV711, and 10 µL anti-CD56-APC) in staining buffer. Single positive control cells for spectral unmixing were prepared using human PBMCs. Details of each antibody are listed in S1 Table. The cells were incubated for 30 min at 4°C, then washed twice with staining buffer, followed by fixing with 1% PFA. Samples were analyzed via flow cytometry using the five-laser Cytek Aurora spectral cytometer (Cytek Bioscience). The data obtained was analyzed using FlowJo Software and results were plotted using GraphPad Prism software.

After confirming the capacity of the panel to identify all the targeted marmoset immune cell populations in PBMCs, the same method was applied for the evaluation of T cell depletion in all the experimental marmosets at different timepoints. Due to the low availability of marmoset PBMCs, human PBMCs were used for single stain controls and spectral unmixing in all cases.

### DNA extraction and qPCR

qPCR was performed on DNA extracted from PBMCs and tissue samples to detect and quantify KSHV viral load. First, 10–20 mg of tissue conserved in RNAlater were digested in 560 µL of extraction buffer (A45470, Thermo Fisher Scientific) containing proteinase K and enhancer solution at 65 °C, shaking at 1,200 rpm (Digital Heating Shaking Drybath; 88880028, ThermoFisher Scientific), for 1.5 h. Then, the lysates were treated with 10 µL of RNase (12091021, Thermo Fisher Scientific) for 10 min at RT and centrifuged for 5 min at 15,000 g. Alternatively, 2 × 10^6^ PBMCs were digested in 480 µL of extraction buffer at 65 °C, mixing (KingFisher DUO, Thermo Fisher Scientific), for 20 min. Similarly, 400 µL of saliva with 40 µL of proteinase K and 40 µL of enhancer solution were incubated in the same conditions. Next, 480 µL of tissue lysate supernatant or the complete volume of PBMC or saliva extract were processed using the MagMAX DNA Multi-Sample Ultra 2.0 Kit (A36570, Thermo Fisher Scientific) in a KingFisher DUO Prime system (5400110, Thermo Fisher Scientific). Purified DNA quantity and quality were assessed using a NanoDrop Lite spectrophotometer (Thermo Fisher Scientific).

TaqMan qPCR reactions for KSHV detection were prepared using KSHV latency-associated nuclear antigen (LANA-1)-specific primers and probe as previously described [82] with the addition of marmoset β-actin as house-keeping (HK) target (see S2 Table for primers and probes sequences). Briefly, each 20 µL reaction contained 1 µL of LANA-1 primer-probe mix (8 µM of each LANA1 primer, 4 µM of LANA1 probe) 1µl of β-actin primer-probe mix (8 µM of each HK primer, and 4 µM of HK probe), 10 µL of 2X QuantiTect Probe PCR Mastermix (204545, Qiagen) and 8 µL of test sample. To quantify the number of LANA-1 copies/sample, a standard curve with known copy numbers was generated using double-stranded DNA gBlocks containing a partial sequence of the LANA-1 gene (S2 Table), ranging from 1 to 10^8^ LANA-1 copies/µL. All samples were tested in duplicates and LANA-1 copy numbers in each test sample were determined by interpolating from the standard curve. Reactions were first heated to 95 °C for 15 min, followed by 50 cycles of incubation at 95 °C for 15 s and 60 °C for 1 min in a QuantStudio 3 or QuantStudio 7 Pro Real-Time PCR System. Results are presented as LANA-1 copy numbers/µg of DNA and were plotted using GraphPad Prism. For graphing purposes, all negative values based on the qPCR detection limit (<1 LANA-1 copies/µg of DNA) were assigned a value of 0.1. All qPCR data is also summarized in a heatmap, displaying ΔCt values (ΔCt = Ct_LANA1_ - Ct_HK_). The color gradient indicates the ΔCT value. In cases in which LANA-1 amplification was detected in only 1 of 2 duplicates, or the Ct value was higher than the last point of the standard curve, samples were re-tested. If similar results were obtained, these samples were reported in the heatmap as positive, but the copy numbers were not reported.

### Anti-KSHV specific ELISA

To detect KSHV seroconversion in all rKSHV-infected marmosets, plasma samples obtained at various timepoints were evaluated for the detection of anti-KSHV VCA IgG antibodies by ELISA. The 96-well MaxiSorp plates (439454, ThermoFisher Scientific) were coated overnight at 4°C with 100 µL per well of either recombinant KSHV VCA antigen produced in *Escherichia* *coli* (custom-made by Genscript) or recombinant LANA-1 antigen produced in insect cells (custom-made by Genscript) diluted in carbonate buffer at 0.5 µg/mL. Plates were subsequently washed three times with 300 µL of washing buffer (0.1% Tween-20 in PBS) and blocked with 200 µL of 3% bovine serum albumin (BSA) in PBS. After a 1 h incubation, plates were washed as described, and 100 µL of 1:25 diluted marmosets’ plasma in dilution buffer (1% BSA in PBS) was respectively added and incubated for an additional 2 h at RT. Next, plates were washed and incubated for 1 h with 100 µL of goat anti-human IgG (H + L)-HRP (C31410, Thermofisher Scientific) diluted 1:2000 in dilution buffer. After a final wash, plates were developed using 100 µL ABST peroxidase substrate solution (5120-0035, LGC SeraCare), followed by the addition of 100 µL stop solution (5150-0017, LGC SeraCare) after 20 min. Optical density (OD) values were measured at 405 nm using a FilterMax F3 (Molecular Device). Cut-off values were calculated as the mean+3SD of the OD values at pre-KSHV infection timepoints for each animal included in that ELISA round.

### Histopathology and immunohistochemistry

Skin lesion FFPE sections (5 µm thick) were deparaffinized with xylene three times for 5 min each (214736-4L, Sigma Aldrich) followed by rehydration with 100% ethanol (2 × 10 min, 4455-4L, Sigma Aldrich), 95% ethanol (2 × 10 min) and dH2O (2 × 5 min). Antigen retrievals were performed by immersing slides in 0.1 mM EDTA, pH 8.0 (15575020, Invitrogen), and heating in a microwave until boiling commenced, followed by incubation at 95 °C to 98 °C for 15 min. Sections were rinsed in dH2O three times for 5 min each, then incubated in 3% hydrogen peroxide for 10 min to block endogenous peroxidase, and washed in dH2O twice for 5 min each. Sections were then washed in TBST for 5 min (9997, Cell Signaling Technology) and blocked with 5% Normal Goat Serum in TBST (Cat. #5425, Cell Signaling Technology) for 2 h at RT. Streptavidin/biotin blocking was performed sequentially, with streptavidin solution (SP-2002, Vector Laboratories) for 15 minutes, followed by a buffer rinse.Primary anti-HHV-8 LANA-1 [LN53] antibody (MABE1109, Sigma Aldrich) diluted in SignalStain Antibody Diluent (8112, Cell Signaling Technology) and then applied to sections (100–400 μL per section), followed by incubation overnight at 4 °C. The next day, the slides were washed in TBST (three times for 5 min each, incubated with 1–3 drops SignalStain Boost Detection Reagent [HRP, Rabbit] (8114, Cell Signaling Technology) for 30 min at RT in a humidified chamber, followed by washing in TBST three times for 5 min each. Detection was done by using Vector NovaRED substrate (SK-4800, Vector Laboratories) for 10 min (100–400 μL per section). Sections were counterstained with hematoxylin (14166S, Cell Signaling Technology), followed by washing twice for 5 min in dH2O, and dehydrated through 95% and 100% ethanol twice for 10 sec each, followed by xylene (2 × 10 sec). Lastly, slides were coverslipped using SignalStain Mounting Medium (14177, Cell Signaling). The slides were scanned by a Leica Aperio AT2 (Leica Biosystems) and later analyzed using QuPath (v 0.5.1).

To detect KSHV LANA-1 antigen in spleen specimens, immunohistochemistry IHC was performed using a Ventana Discovery Ultra automated system (Roche Diagnostics). FFPE sections (5 μm) were mounted on positively charged slides and deparaffinized using EZ Prep (950–102, Roche) combined with heat at 75°C for 8 min. Next, samples were subjected to heat-induced epitope retrieval (HIER) by incubating slides with Discovery CC1 (950–123, Roche) for 64 min at 95°C. Endogenous peroxidase activity was blocked using inhibitor (760–4840, Roche), followed by a 60 min incubation at 37°C with rat anti-KSHV LANA1 monoclonal antibody (LN53) (1:1000) [83]. Slides were then incubated with a secondary rabbit anti-rat IgG (ab133469, Abcam, 1:500) followed by anti-rabbit-HQ (760–4815, Roche) and anti-HQ-HRP (760–4820, Roche). Signal was amplified using Amplification HQ kit (760–052, Roche) and Amplification anti-HQ-HRP Multimer (760–4602, Roche). Slides were washed after each staining step. Immunoreactivity was visualized with a DAB detection system (760–159, Roche), after which sections were counterstained with hematoxylin (07024282001, Roche), cover-slipped, and visualized. All staining washes and reagent dilutions were performed using manufacturer-recommended buffers and conditions. Assay validity was confirmed using positive FFPE human KS lesion-tissues included in each run. A nuclear red-brown staining was considered positive; staining scores are described in the main text. Staining adequacy was independently assessed and verified by the City of Hope Research Pathology Core.

### Statistical analysis

All statistical analyses were performed using GraphPad Prism software. Unpaired t-test was used to compare the means of two independent groups; one-sample t-test was used to assess changes in the means within a single group over time, followed by a paired t-test to evaluate the significance of differences between two specific timepoints; Mann-Whitney U test was used to compare the significance of differences between the distributions of two groups; two-way ANOVA was used to compare different groups at more than two timepoints; one-way ANOVA with Geisser-Greenhouse correction followed by Dunnett’s multiple comparison test was used to assess changes over time within each treatment group; Spearman’s correlation test was used to identify correlations between variables. Statistical significance references and applied tests in each experiment are shown in the figure legends.

### Figures and diagrams

All data graphs and figures were generated using GraphPad Prism software. Marmoset experimental schedule diagrams were generated using Biorender.com.

## Supporting information

S1 Table Validated mAb panel to detect common marmoset immune cell populations by flow cytometry analysis. (TIF)

S2 Table List of primers used for qPCR determinations. (TIF)

S1 Fig PBMC viremia and anti-KSHV VCA and LANA-1 IgG levels in rKSHV-infected common marmosets treated with oral tacrolimus, and immune profiling of rKSHV-infected common marmosets treated with intravenous (I.V.) or oral tacrolimus. **(A)** Quantification of LANA-1 gene copy numbers per µg of DNA isolated from PBMCs (i.e., PBMC viremia) of challenged marmosets treated with oral tacrolimus over time using qPCR. Each datapoint represents the mean of two replicates. **(B-C)** Measurement of anti-KSHV VCA IgG (B) and anti-KSHV LANA-1 IgG (C) levels in plasma diluted 1/25 from challenged marmosets treated with oral tacrolimus over time using ELISA. Each datapoint represents the mean of two replicates. Cut-off values were calculated as described in Materials and methods and are represented using a dotted line. Viral challenge and tacrolimus treatment initiation are indicated by green and red arrows, respectively. **(D-E)** Quantification of total T cell (CD3^+^), CD4^+^ T cell, CD8^+^ T cell, B cell (CD20^+^), NK cell (CD56^+^), and monocyte (CD14^+^) populations in PBMCs of challenged marmosets treated with I.V. (D) or oral tacrolimus (E) over time using flow cytometry. Each datapoint represents the mean of three technical replicates. Viral challenge and tacrolimus treatment initiation are indicated by green and red arrows, respectively.
(TIF)

S2 Fig Skin lesion development in rKSHV-challenged marmoset after daily intravenous (I.V.) tacrolimus treatment. **(A)** Gross images of skin lesions in rKSHV-challenged marmoset after daily tacrolimus treatment. Marmoset Cj062 from experiment 1, described in Fig 1, developed a non-resolving skin lesion in the middle of the back detected on day 280 after the first KSHV challenge (62 days after initiation of tacrolimus treatment), which grew to 6 mm in diameter over a period of 10 days. Images were recorded on the indicated days. **(B)** Histopathological examination of the skin lesion biopsy; hematoxylin and eosin (H&E) staining (top row) and LANA-1-specific immunohistochemistry (IHC) staining (bottom row), observed in two different fields of the same section (left and right). Newly formed blood vessels and spindle-shaped cells, characteristic features of KS pathology, are indicated in the H&E staining by dashed and solid black arrows, respectively.
(TIF)

S3 Fig Validation of anti-CD4 [L200] and anti-CD8 [HIT8a] for detection of T cells populations in common marmosets via flow cytometry. **(A-B)** Quantification of CD3^+^ CD4^+^ **(A)** and CD8^+^ **(B)** T cells in marmoset splenocytes by flow cytometry using marmoset-chimerized anti-CD4 [TAWA] and anti-CD8 [OCHO] or commercial flow cytometry validated anti-CD4 [L200] and anti-CD8 [HIT8a]. Marmoset splenocytes or human PBMCs (control) were co-stained with anti-CD3 [Sp34–2] and either anti-CD4 [TAWA] or anti-CD8 [OCHO] to validate their ability of to bind marmoset T cells (CD3^+^). Dot plots to the left display marmoset splenocyte cell populations stained with anti-CD3 [Sp34–2] (x-axes) and anti-CD4 [TAWA or L200] or anti-CD8 [OCHO or HIT8a] (y-axes), while the dot plots to the right display the % of the CD4^+^ or CD8^+^ T cell population within the T (CD3^+^) cell population. The bar graphs summarize the marmoset dot plot data in comparison with human PBMCs. Each bar represents the mean ± standard deviation of three technical replicates. Statistical significance was determined by unpaired t-test; ns = not significant. **(C-D)** Flow cytometry competition assay in marmoset splenocytes between anti-CD4 [TAWA]-AF647 and [L200]-PercpCy5.5 (C), and anti-CD8 [OCHO]-AF488 and [HIT8a]-BUV805 (D). Marmoset splenocytes were either sequentially stained by the mAb pair of interest (mAb1 → mAb2) or stained simultaneously with a cocktail of both mAbs (mAb1 + mAb2), then analyzed by flow cytometry to assess competitive binding. Bar graphs denote detection of each mAb through the corresponding conjugated fluorophore, for each staining condition tested. Each bar represents the mean of three technical replicates. Statistical significance was determined by unpaired t-test; ns = not significant, **** = p < 0.0001.
(TIF)

S4 Fig Validation of a compatible mAb panel for flow cytometry analysis of monocytes and lymphocytes cellular subpopulations to be used in common marmoset treated with anti-CD4 [TAWA] and anti-CD8 [OCHO]. **(A-B)** Flow cytometry gating strategy for detection and quantification of different immune cell populations in marmoset (A) and human (B) PBMCs, using the antibodies listed in S1 Table. Leukocytes (CD45^+^) were first gated from the live PBMC population, which were then divided into monocytic cells (CD45^+^ SSC^int^) or lymphocytes (CD45^+^ SSC^low^). Using the corresponding markers, classical monocytes (CD14^+^CD16^-^) and non-classical monocytes (CD14^+^CD16^+^) were gated from the monocytic cell population, and T cells (CD3^+^), B cells (CD20^+^), and NK cells (CD3^-^CD20^-^, CD8^+^/^-^CD56^+^) were gated from the lymphocyte population. CD4^+^ and CD8^+^ T cells were then gated from the main T cell population. (**C**) Flow cytometry analysis of marmoset PBMCs (n = 5, top row), marmoset splenocytes (n = 5, middle row), and human PBMCs (n = 5, bottom row) for quantification of the indicated immune cell populations using the gating strategy described in panel (A-B). Each datapoint represents the mean percentage of three technical replicates for the indicated cell population measured in an individual marmoset or healthy human donor.
(ZIP)

S5 Fig Immune cell population frequencies and derived cellular counts over time in mAb-mediated immunosuppressed and immunocompetent marmosets. **(A)** PBMCs cell count per mL of processed blood in immunosuppressed and immunocompetent marmosets over time. Statistical significance was determined by two-way ANOVA; * = p < 0.05. **(B)** Quantification of B cells (CD20^+^), NK cells (CD56^+^), and Monocytes (CD14^+^) in PBMCs of mAb-treated marmosets over time using flow cytometry. Each datapoint represents the mean of three technical replicates. Statistical significance of immunosuppressed vs. immunocompetent groups at each timepoint was determined by two-way ANOVA; ns = no significant. **(C)** Derived absolute cell counts of B cells (CD20^+^), NK cells (CD56^+^), and Monocytes (CD14^+^) per mL of blood. These values were calculated by applying the frequency values obtained by flow cytometry to the number of PBMCs obtained per mL of blood as reported by SNPRC. Each datapoint represents the mean of three technical replicates. Statistical significance of immunosuppressed vs. immunocompetent groups at each timepoint was determined by two-way ANOVA; ns = no significant.
(TIF)

S6 Fig Chimeric mAb-mediated immunosuppression after individual or sequential administration of mAbs, and intravenous (I.V.) rKSHV infection of common marmosets. **(A)** Experimental marmoset treatment and sample collection schedule. n = 22 marmosets were distributed into 4 treatment groups, as indicated on the treatment group summary table on the left of the panel. Marmosets were treated I.V. with either single anti-CD4 [TAWA] or anti-CD8 [OCHO], anti-CD4 [TAWA] followed by (f/b) anti-CD8 [OCHO], or PBS, according to treatment group (10mg/kg each mAb), on the indicated timepoints. *: Anti-CD8 administration timepoints for anti-CD4 f/b anti-CD8 group. Marmosets were subsequently challenged I.V. with rKSHV BAC16 (5 × 10^5^ HEK-293 IU) on D0. Blood samples were collected throughout the study on the indicated timepoints; multiple tissues were collected for analysis at the indicated study endpoint*. Created in BioRender. Han, L. (2026)* **https://BioRender.com/kyk51dr*.* **(B)** Quantification of total T cells (CD3^+^), CD4^+^ and CD8^+^ T cells in PBMCs of mAb-treated marmosets over time using flow cytometry. Each datapoint represents the mean of three technical replicates. Statistical significance between two timepoints within the same group was determined by paired t-test; ns = no significant, * = p < 0.05, ** = p < 0.01, *** = p < 0.001.
(TIF)

S7 Fig Functionality assessment of anti-CD4 [TAWA] mAb used in individual and sequential mAb administration study in common marmosets. **(A)** Quantification of anti-CD4 [TAWA] and anti-CD8 [OCHO] plasma levels in marmosets described in S6 Fig over time using ELISA. Each datapoint represents the mean of two technical replicates. Statistical significance between two timepoints within the same group was determined by paired t-test; ns = no significant, * = p < 0.05, ** = p < 0.01, **** = p < 0.0001. Anti-CD4 [TAWA] and anti-CD8 [OCHO] treatment initiations are indicated by blue and black arrows, respectively. **(B)** Anti-CD4 [TAWA] binding assessment to rhesus CD4 using ELISA. The ability of the TAWA mAb used for the study described in S6 Fig (lot JP222201) to bind marmoset CD4 was measured by ELISA, as compared to a freshly produced TAWA mAb (lot 00128) and the parental murine anti-CD4 [19Thy5D7]. ELISA was performed using different concentrations of each mAb, as indicated on the panel, at a fixed rhesus CD4 coating antigen concentration. Each datapoint represents the mean of two technical replicates.
(TIF)

S8 Fig Quantification of immune cell population frequencies and PBMC viremia over time after anti-CD8 [OCHO]-mediated immunosuppression and intravenous (I.V.) rKSHV infection of common marmosets. **(A)** Quantification of B cells (CD20^+^), NK cells (CD56^+^) and monocytes (CD14^+^) in PBMCs of marmosets described in Fig 6 over time using flow cytometry. Each datapoint represents the mean of three technical replicates. Statistical significance between two timepoints within the same group was determined by one-sample t-test, followed by the paired t-test; ns = no significant, * = p < 0.05, ** = p < 0.01. Anti-CD8[OCHO] mAb treatment initiations at D-21 or D-12 are indicated by blue and yellow arrows, respectively. Matching colors are used to denotate differences within a single group. **(B)** Quantification of LANA-1 gene copy numbers per µg of DNA isolated from PBMCs (i.e., PBMC viremia) of challenged marmosets over time using qPCR. Each datapoint represents the mean of three replicates.
(TIF)

S9 Fig Histopathological examination of skin lesions developed in anti-CD8 [OCHO]-treated marmosets and non-depleted groups upon rKSHV challenge. **(A)** Gross images of skin lesions in rKSHV-challenged marmosets previously treated with anti-CD8 [OCHO] or mock anti-CD4 [TAWA], described in Fig 6. The listed marmosets developed suspicious skin lesions at different timepoints, and the images were recorded on the indicated days, after which biopsies from the lesions were collected. **(B-C)** Histopathological examination of the skin lesions after biopsy, including (B) hematoxylin and eosin (H&E) staining and (C) LANA-1-specific immunohistochemistry (IHC) staining. As controls, distal skin from the same marmosets, as well as back skin samples from independent healthy marmosets, were also examined. Spindle-shaped cells and newly formed blood vessels, characteristic features of KS pathology, are indicated by solid and dashed black arrows, respectively. For the independent healthy marmosets, the original animal IDs have been replaced with arbitrary IDs for ID confidentiality as requested by SNPRC.
(TIF)

S10 Fig Summary heatmap of PBMC viremia and KSHV viral load in tissues. Summary heatmap displaying results of KSHV LANA-1 qPCR in DNA isolated from indicated cells or tissues (x-axis) in each individual marmoset enrolled in this study (y-axis; Animal ID, sex, and group distribution are indicated). Heatmap colors/numbers denote ΔCt values, where ΔCt is defined as the difference between the LANA-1 gene Ct and the β-actin housekeeping (HK) gene Ct (ΔCt = Ct_LANA1_ - Ct_HK_); a lower number denotes a higher abundance of LANA-1 DNA. White indicates that LANA-1 DNA was not detected in the sample. A cross (X) indicates that analysis was not performed due to sample unavailability. A slash (/) indicates that the LANA-1 DNA could not be quantified due to either a single replicate amplification or the Ct value falling outside of the standard curve linear range. Original animal IDs of the uninfected animals have been replaced with arbitrary IDs for ID confidentiality as requested by SNPRC.
(TIF)

S11 Fig KSHV LANA-1 protein expression and correlation with viral load in spleens from marmosets included in all studies. KSHV LANA-1 IHC to evaluate LANA-1 expression was performed in the spleen of all marmosets enrolled in this study. Samples were classified using an arbitrary IHC scoring system based on three distinct staining criteria: a) color – cell must display the characteristic red-brown diaminobenzidine (DAB) staining signal; b) localization – the DAB signal must be clearly defined and restricted to the cell nucleus, as confirmed by counterstaining; and c) distribution – positive cells must be detectable in at least three independent fields of view at 10× magnification. Based on these criteria, samples were assigned one of four IHC scores (S11A Fig): IHC 0 (LANA-1 negative) – sample failed to meet color and localization criteria; IHC 1 (LANA-1 inconclusive) – sample met the color and localization criteria but fails to meet the distribution criterion; IHC 2 (LANA-1 positive) – sample met all the three criteria, with two or fewer LANA-1-positive cells observed per field; and IHC 3 (LANA-1 positive frequent) – sample met all three criteria, with more than two LANA-1-positive cells observed per field. Results were compared with those obtained for viral load in the same tissue by LANA-1 qPCR. **(A)** Table summarizing the IHC score and viral load obtained for the spleen of each marmoset enrolled in this study. **(B)** Comparison of KSHV LANA-1 IHC scores against LANA-1 copies/μg of DNA in spleens from marmosets included in each experimental group as represented by different colors. Only tissues with detectable viral load were included as all undetectable corresponded with an IHC 0 as shown in (A). No correlation between viral load and IHC score was observed (Spearman test: r = 0.2097, p = 0.3039).
(TIF)

S12 Fig KSHV LANA-1 IHC staining of spleen from marmosets classified as IHC 2 and IHC 3. KSHV LANA-1 IHC spleen staining slides for marmosets described in S11 Fig. and classified as LANA-1 positive (IHC 2) and LANA-1 positive frequent (IHC 3). Scale: black bar denotates 250 μm in each main panel captured at 10× magnification; black bar denotates 50 μm in each zoomed-in panel captured at 40× magnification. LANA-1 staining appears in red-brown. Treatment, marmoset ID and IHC score are displayed on the left side of each panel. One uninfected marmoset (Cj70005) was included as LANA negative control (IHC 0).
(TIF)
