# Supplementary material for: Newly developed monoclonal antibodies for marmoset immunosuppression enable KSHV lytic replication and salivary shedding: A promising model for KSHV pathogenesis studies
Source: PLoS Pathog. 2026 Oct 26;22(10):e1014698. doi: 10.1371/journal.ppat.1014698 (PMC13722297; doi:10.1371/journal.ppat.1014698)
Supplement: Supplementary file 1 [file ppat.1014698.s016.pdf]

Dear Editor,

We thank the PLOS editorial team and the Reviewers for their thoughtful and constructive feedback on our manuscript resubmission (PPATHOGENS-D-26-00589R1), titled "*Newly developed monoclonal antibodies for marmoset immunosuppression enable KSHV lytic replication and salivary shedding: a promising model for KSHV pathogenesis studies*," and for the opportunity to submit a revised version.

In response to the Reviewers' comments, we have revised the manuscript and conducted additional experiments that we believe further strengthen the overall impact of the study. Below, we first provide a concise summary of the newly generated data and major changes and additions to the manuscript, followed by a detailed, point-by-point response to each Reviewer's comment.

We appreciate the time and effort invested by PLOS Pathogens editorial team and Reviewers in evaluating our work and thank you for the opportunity to improve it. We look forward to your consideration of this revised submission.

<u>Summary of changes and additions to the original submission</u>

1) **Inclusion of new LANA-1 immunohistochemistry data.** To further improve the rigor of our infection outcome assessments, we have now performed LANA-1 immunohistochemistry (IHC) analysis in marmoset spleens. Importantly, our results revealed LANA-1 expression in spleens from a subset of animals in every rKSHV-challenged group, confirming true infection. To discuss these results in context of our qPCR assessments, we also performed an analysis where we compared the IHC scores of each spleen to their corresponding qPCR DNA values, showing that qPCR was more sensitive in detecting the virus.
2) **Inclusion of new anti-KSHV LANA-1 IgG ELISA data.** To further characterize the antibody response against KSHV infection, we have now performed ELISA to detect antibodies against LANA-1. We were able to detect plasma LANA-1 antibodies in two animals and now report these results in the revised text.
3) **Expanded discussion on study limitations and guidance for future studies using this model.** Following Reviewers' suggestion, we have revised the Discussion to include a comprehensive paragraph outlining general study limitations not previously discussed and provided recommendations for future studies looking to further characterize or use the marmoset KSHV challenge model.
4) **Additional textual changes and changes in manuscript language and title.** We have incorporated additional edits and corrections throughout the manuscript in response to the Reviewers' comments, the main being that we have re-worded "lytic reactivation" to "lytic replication" when referring to our results, including in the manuscript title. This change improves clarity and accuracy, better reflecting the conclusions of this work.

Response to Reviewers' feedback

REVIEWER 1

Summary & Major issues: Key Experiments Required for Acceptance

A. *The most significant concern involves inconsistent viral quantification methodologies across experiments [i.e. use of different indicator cell lines, varying viral inocula, and use of both absolute copy numbers and ΔCt values for viral load measurements]. These methodological inconsistencies complicate the interpretation of infection outcomes and limit reproducibility assessments. […] While the viral quantification issues require attention, they do not undermine the fundamental contributions of establishing this novel experimental system. / The inconsistent viral quantification approaches, while important limitations, reflect the practical challenges of developing this novel model system. Requiring standardization experiments would impose an unreasonable burden given that the fundamental scientific conclusions are well-supported by the existing data.*

RESPONSE: We thank the Reviewer for recognizing the inherent challenges of our work and agree that standardization experiments will be key for future studies to ensure consistency and reproducibility. We now further discuss these limitations in a new paragraph in the Discussion. Regarding the use of absolute copy numbers vs ΔCt values, we would like to highlight that in this work, we report LANA-1 copy numbers as copies per microgram of DNA when Ct values fell within the linear range of the standard curve in both technical replicates; if Ct values for one or both technical replicates fell outside of the linear range, we did not quantify copy numbers and instead display results as the change in Ct, defined as the difference between the LANA-1 gene Ct and the β-actin housekeeping (HK) Ct ($\Delta Ct = Ct_{LANA\text{-}1} - Ct_{HK}$), and refer to these DNA levels as traces. We have defined this in the corresponding section of the Results.

B. *The study also encounters practical challenges including loss of anti-CD4 antibody functionality during experiments, necessitating post-hoc experimental restructuring. While the authors demonstrate that replacement batches restore binding activity, the in vivo validation of these reagents remains limited. / The loss of anti-CD4 antibody functionality during the study, while concerning for future applications, does not invalidate the core findings since the authors demonstrate restoration of activity with replacement reagents and provide appropriate documentation of this issue.*

RESPONSE: We thank the Reviewer for recognizing and appreciating our efforts to be transparent, and acknowledge in our manuscript that regularly checking antibody functionality before and during experiments will be essential for future immuno-depletion experiments.

C. *No additional experiments are required for acceptance. The current data, particularly the demonstration of salivary viral shedding during immunosuppression, provides sufficient evidence for the authors' conclusions regarding KSHV reactivation in this model system. The methodological concerns identified can be adequately addressed through textual modifications rather than new experimental work.*

RESPONSE: We thank the Reviewer for their recognition our efforts. As noted in our response to **Comment A**, we have added a paragraph on the Discussion that further addresses the study's limitations and highlights lessons learned to inform future studies.

Minor Issues: Editorial and Data Presentation Modifications

D. *Viral Quantification Methodology Discussion: The authors should add a comprehensive discussion in the Methods section comparing the different viral quantification approaches used across experiments. Specifically, they should: (1) Acknowledge that different indicator cell lines (iSLK vs HEK293) produce non-comparable infectious unit measurements; (2) Explain the rationale for using different viral inocula across experiments; (3) Discuss the limitations of mixing absolute copy numbers with ΔCt relative measurements; and (4) Provide recommendations for standardized quantification methods in future marmoset KSHV studies based on their experience. / Enhanced Limitations Discussion: Expand the Discussion section to more thoroughly address the methodological inconsistencies and their potential impact on data interpretation, while maintaining appropriate context about the pioneering nature of this work. These modifications will*

significantly strengthen the manuscript's methodological transparency and provide valuable guidance for future studies utilizing this novel model system.

<u>RESPONSE:</u> We agree with the Reviewer that a comprehensive discussion of the various limitations and lessons of the study will greatly benefit this work, and we have now added a dedicated paragraph to discuss the relevant listed points in the revised Discussion, as described earlier. Regarding the use of absolute copy numbers vs ΔCt values, please refer to our response to **Comment A**.

E. *Flow Cytometry Validation Concerns: Address the potential interference between therapeutic TAWA antibody and detection antibodies in flow cytometry assays. The observed signal reduction upon co-incubation with L200 suggests possible epitope competition that could affect CD4+ T cell quantification accuracy. The authors should discuss this limitation and its potential impact on depletion measurements.*

<u>RESPONSE:</u> During the flow cytometry validation experiments, we did not observe any reduction in signal under the sequential incubation condition of TAWA → L200. Because this sequential exposure reflects the expected *in vivo* scenario, where marmosets receive TAWA before PBMC isolation and subsequent L200 staining, we do not anticipate that epitope competition could affect the accuracy of our $CD4^+$ T cell quantification or depletion measurements. However, because signal reduction signal reduction is observed when the two mAbs are co-incubated at the same time, we have added a statement to the revised manuscript advising that future studies should carefully consider the order of antibody incubation.

F. *Antibody Stability and Replacement Documentation: Provide more detailed discussion of the anti-CD4 antibody functionality loss and the validation of replacement batches. While the authors appropriately acknowledge this issue, they should expand on the implications for reproducibility and provide stronger justification for their conclusions about TAWA reliability / Sampling Strategy Limitations: The authors mention potential false negatives in tissue assessments due to localized infection. They should briefly discuss strategies to mitigate this in future studies, such as sampling multiple sites within organs or using larger tissue sections.*

<u>RESPONSE:</u> We thank the Reviewer for their recommendation; we have further addressed the study limitations in our revised manuscript.

G. *Editorial Corrections: (1) Line 464: Remove word duplication ("quantify" appears twice); (2) Ensure consistent nomenclature for viral strains and antibody designations throughout.*

<u>RESPONSE:</u> We have now removed the duplicated word and checked all nomenclature for consistency.

REVIEWER 2

Summary & Major issues: Key Experiments Required for Acceptance

A. *This reviewer's prior concerns have been addressed and the study is now suitable for publication.*

RESPONSE: We thank Reviewer 2 for their encouraging evaluation. Reviewer 2 did not request any further revisions, but we have nevertheless re-checked the manuscript for clarity and consistency in light of all reviewer feedback, and incorporated the necessary changes to address Reviewer 1, 3 and 4 comments.

Minor Issues: Editorial and Data Presentation Modifications

No comments.

REVIEWER 3

Summary & Major issues: Key Experiments Required for Acceptance

H. *Efficient infection was only observed in suppressed animals and was hardly detectable in normal animals even upon intravenous injections with a very high dose of virus.*

RESPONSE: We appreciate this Reviewer's concern. However, our data indicate that KSHV infection was established in most animals regardless of immunosuppression status. Indeed, quantifiable viral DNA was detected in the spleen and liver of animals across all treatment groups as shown in **S10 Fig**, including those receiving PBS or Mock anti-CD4 treatment, which serve as immunocompetent controls. Furthermore, our newly performed anti-KSHV LANA-1 IHC (**S12 Fig**) demonstrates the presence of LANA-1-expressing cells in a subset of animals from different treatment groups, providing independent evidence of KSHV infection.

I. *Infection and response to the infection was also highly variable from one animal to animal, as shown in figure 5. Only one animal cj2254 showed a notable anti-VCA antibody response and the others did not, making the marmoset still not a reliable infection model for pre-clinical study. [It is possible that combining the results from different studies and protocols, from different batches of animals and from different primate centers due to the inherent constraints of this animal model could have] contributed to the variability of the studies.*

RESPONSE: In the first mAb depletion experiment (**Fig 5**), two out of four animals displayed anti-VCA Ab levels above baseline. In contrast, in the second mAb depletion experiment, the majority of animals developed detectable anti-VCA Abs, under both immunosuppressed and immunocompetent conditions (**Fig 7**), with variable responses. *To facilitate interpretation, we have now indicated the baseline cut-off on all anti-VCA ELISA graphs and described how this cut-off was established in the revised Materials and methods*. We acknowledge that differences between experiments may have contributed to the observed variability, and have now added a new paragraph in the revised Discussion that further addresses the limitations of the study. We would also like to highlight that common marmosets represent an outbred population, and therefore their immune responses are expected to more closely resemble those observed in the human population, where substantial inter-individual variability is common (PMID: 27062052; PMID: 18515794), rather than the relatively uniform responses typically observed in inbred laboratory animal strains. Therefore, the observation that the marmosets mounted broadly similar immune responses despite both biological and experimental factors supports the robustness of the model.

J. *Another concern is the use of different strains of KSHV, different doses of viruses in different experiments, […] different sources of KSHV, which can potentially create substantial variability in various aspects of the study.*

1

RESPONSE: The variations in our challenge inoculum titration and dosing reflect different regimens adopted in previous studies. Specifically, our use of rKSHV.219 and rKSHV BAC16 are consistent with the two published KSHV challenge studies in marmosets (PMID: 19798430 and Pre-print https://doi.org/10.1101/2024.10.31.621273, respectively). Likewise, our titration methods are in line with previous KSHV challenge studies in humanized mice (HEK-293, PMID: 38844783), tree shrews (HEK-293, PMID: 34603235), and marmosets (iSLK, Pre-print https://doi.org/10.1101/2024.10.31.621273). We acknowledge that further refinement of the model, including the use of standardized challenge doses and inoculum preparation methods, will be important. Accordingly, we have expanded the Discussion in the revised manuscript to address these limitations and outline considerations for future studies.

K. *The manuscript was also very poorly put together, including data from experiments that were not optimized, such as showing cell percentages rather than absolute cell counts, using antibodies with stability not verified, all resulting in concerns with the presented results.*

RESPONSE: We intentionally presented and discussed the data in a transparent manner to highlight the inherent challenges associated with developing and validating this model. This approach was recognized and commended by other Reviewers during both the current and the original rounds of review. We acknowledge the limitations identified in our study, including those noted by the Reviewer, and have expanded the Discussion in the revised manuscript to more thoroughly address these issues and provide guidance for the design and interpretation of future studies.

L. *[…] it is not clear on the rationale that [tacrolimus] was again used for this study in the earlier experiment, which has now demonstrated that its use was inadequate due to its toxicity. This has distracted away from the major focus of this manuscript, which centers on the development and effectiveness of the chimeric antibodies in enhancing KSHV infection*.

RESPONSE: Before our study, tacrolimus had only been administered in this context before KSHV challenge, and this was hypothesized to have interfered with establishment of infection (PMID: 19798430); there were no reports of toxicity in this early work. Therefore, one of the initial objectives of our study was to further confirm the susceptibility of the marmoset to KSHV challenge and assess the effect of tacrolimus administered post-challenge. The toxicity we subsequently observed, together with the difficulty of directly monitoring tacrolimus-mediated immunosuppression, prompted us to develop mAbs as a safer, more targeted, and experimentally tractable immuno-depleting alternative, as outlined throughout our manuscript. We believe it is critical to present this progression of experiments to transparently document the development of the model, provide the rationale for the antibody-based approach, and help prevent unnecessary duplication of unsuccessful strategies in future studies.

M. While higher copy of the viral DNA can be detected in tissues in suppressed animals, and viral DNA can be detected in the saliva DNA. However, it can only be detected upon immune suppression, and no viral DNA in the PBMC was detected. This does not reflect the natural history of infection in humans, where there were periodic viremia and viral shedding in saliva in asymptomatic KSHV infected individuals without immure suppression. Therefore, it is not clear that marmoset will be optimal small animal model to study KSHV disease pathogenesis. / It is not clear why viral DNA can be detected in the saliva of suppressed animals but no viral DNA in the blood was detected at all studied time points, which is normally not the case in humans. / Similarly, the authors have suggested the viral reactivation in the infected tissues led to an increase in anti-KSHV IgG in the study animals. This will be surprising since no viral DNA was detected in the blood PBMC.

RESPONSE: We agree with the Reviewer that further characterization of viral kinetics is needed to fully characterize the common marmoset as a model for KSHV pathogenesis. However, the absence of detectable viral DNA in PBMCs despite the presence of viral DNA in saliva is not necessarily inconsistent with human KSHV infection. In humans, oral viral shedding can occur in the absence of detectable viremia (PMID: 31555829), reflecting an inherent variability in infection kinetics and virus control across individuals and infection compartments. Thus, our findings remain within the spectrum of observations reported in naturally infected individuals. It is also possible that our limited sampling frequency and sample volumes reduced our ability to detect transient or low-level viremia. These limitations are now discussed in the revised Discussion. Regarding antibody responses, the presence of anti-KSHV antibodies in the absence of detectable viremia is consistent with previous reports in KSHV-infected individuals (PMID: 27062052) and may not be unique to KSHV, as recent studies reported that asymptomatic COVID-19-infected people may not display COVID-19 viremia (PMID: 37515246) but can still mount IgG responses against the virus detectable in the serum (PMID: 33031941, PMID: 35136139). Ultimately, larger longitudinal studies with more frequent sampling and greater statistical power will be required to fully define the kinetics of viremia, viral shedding, tissue infection, and immune responses in this model. These important studies, however, were beyond the scope and resources of the present work.

N. Figure 3 showed substantial suppression of both CD4 and CD8 cells as early as 8 hours after treatment due to non-optimized analyses of the cells. This differed from data shown in figure 4 from other animals studied. It is unclear why the authors chose to present data that are not properly analyzed to begin with.

RESPONSE: We appreciate the Reviewer's concern. The marmosets shown in **Fig 4** were part of an independent experiment with differing study objectives and were sampled 14 days after mAb administration. Therefore, these data are not directly comparable to the early post-treatment timepoints presented in **Fig 3**. Indeed, **Fig 4** was intended as an independent validation of *in vivo* activity of the anti-CD4 [TAWA] and anti-CD8 [OCHO] mAbs, rather than as a direct comparison to the experiment in **Fig 3**.
As described in the manuscript, the analyses in **Fig 3** were performed before implementation of the optimized flow cytometry Ab panel, and the CD8 analysis in **Fig 3B** is limited by the use of anti-CD8 [OCHO] as both

depleting and detection antibody. We chose to retain these data for transparency and to document the evolution of the experimental approach. To minimize potential confusion, we have revised the figure legend to explicitly note this limitation and direct readers to the main text for clarity.

O. *Infection in the tissues were all demonstrated by qPCR reflecting copies per microgram DNA, but it is not clear these are truly from infected cells harboring intact latent viruses or they were from remnants of archived viral DNA. IHC detection of viral protein to demonstrate infected cells will be needed.*

RESPONSE: We agree with the Reviewer that immunohistochemical confirmation would strengthen the interpretation of tissue infection. Given that the tissue samples were collected more than 100 days post-challenge, we consider it unlikely that the detected viral DNA represents residual inoculum rather than established infection. To confirm this, following Reviewers' recommendations, we performed KSHV LANA-1 IHC experiments and now show the presence of LANA-1-expressing cells in the spleen of marmosets from all challenged groups (**S12 Fig**), providing independent evidence to support the presence of KSHV-infected cells in this model.

P. *It is not clear why the authors have included the skin lesion in the supplement data, indicating that it is KS-like but with no detectable LANA, and presumably no detectable viral DNA. This could simply be a skin disease upon prolonged immune suppression.*

RESPONSE: We respectfully clarify that we did not describe these lesions to be KS-like in our previous or current resubmission, as we found no evidence of KSHV infection in the lesion tissue. We also cannot attribute the lesions solely to immunosuppression, as a lesion was also observed in an infected immunocompetent marmoset (**S9 Fig**: Cj2315, Mock anti-CD4 group). Although these may be considered "negative" results, we opted to include them for transparency as an interesting finding, particularly in light of the original marmoset study by the Jung group which reported a lesion with confirmed KSHV markers in an infected marmoset (PMID: 19798430).

Q. *Figure 5C showed the same symbols for all 5 animals studied. They need to be changed.*

RESPONSE: We have now updated Fig 5C so that symbols and colors for the four challenged marmosets are consistent with the other panels in the figure.

R. *Introduction, line79. It is not true that KSHV infected individuals over 50 are at risk for endemic or classical KS. It is true only in certain populations in certain parts of the world.*

RESPONSE: We thank the Reviewer for their observation and have clarified this sentence accordingly.

S. *The authors need to mention the rhesus macaque model. It has been extensively studied and had led to a better understanding of the KSHV pathogenesis even though this model was infected by the simian homologue of KSHV*.

RESPONSE: We thank the Reviewer for their suggestion and have now referenced the rhesus macaque/RRV model in the Introduction when introducing NHP models.

T. *Line 308, CD4+/CD3- cells are not necessary accessory cells in the stromal in spleen, they could be monocytes/macrophages.*

RESPONSE: To our knowledge there are no previous studies reporting the expression of CD4 on marmoset monocytes or macrophages. To further investigate this possibility, we examined our existing multi-parameter flow cytometry datasets to determine whether marmoset PBMC monocytes expressed CD4. As shown in **R1 Fig** below, we were unable to detect CD4 in marmoset monocytes. Given that our flow cytometry panel did not include markers to identify macrophages, we could not investigate this further; nevertheless, our observations are in line with the fact that expression of CD4 in human monocytes is not a well-conserved feature across species (PMID: 24942581).

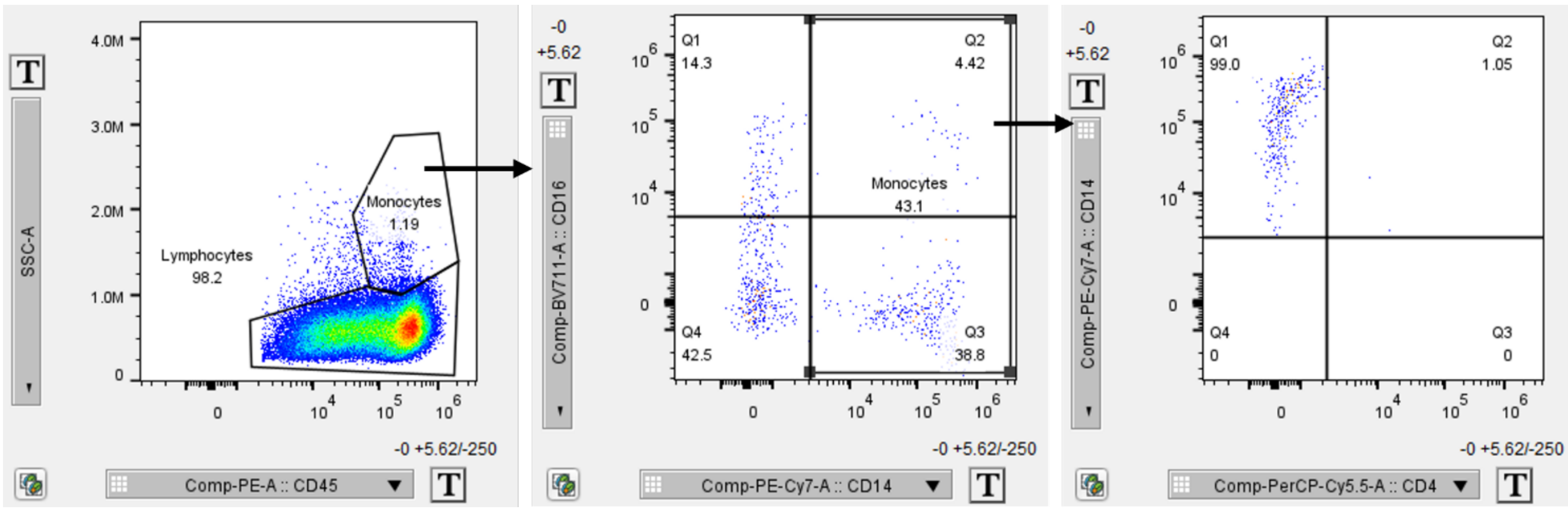

R1 Fig. Assessment of CD4 expression in marmoset monocytes. Marmoset PBMCs stained with the optimized flow cytometry Ab panel described in the manuscript were analyzed using the shown gating strategy to assess CD4 expression in monocytes.

<u>Minor Issues: Editorial and Data Presentation Modifications</u>

No comments.

REVIEWER 4

Summary & Major issues: Key Experiments Required for Acceptance

A. *There is no direct evidence that CD8 T cells respond to KSHV infection such as by immune phenotype, effector cytokine production or cell-killing. / Is a virus-specific T cell response generated in non-depleted animals? ELISPOTS can be used to profile IFNgamma or TNF production from splenocytes incubated with KSHV antigens.*

RESPONSE: We agree with the reviewer that defining and characterizing the virus-specific cellular responses elicited by KSHV challenge in marmosets will be important for the continued development and validation of this model. However, these studies were beyond the scope of the present work and were not included in the original study design. We plan to address these questions in future studies using standardized experimental approaches specifically designed to evaluate antigen-specific T cell responses, including effector cytokine production and other functional immune correlates.

B. *There is no direct evidence that CD8 T cell depletion leads to more KSHV reactivation from latency.*

RESPONSE: We agree with the Reviewer that our data do not provide direct evidence that $CD8^+$ T cell depletion induces KSHV reactivation from latency. We now show evidence of LANA-1 expression in spleen (**S12 Fig**), demonstrating that KSHV-infected cells in this model can indeed establish a latent infection. However, we acknowledge that these findings do not demonstrate that the observed viral shedding results from a transition from latency to the lytic cycle. Accordingly, we recognized that the term *"lytic reactivation"* was not sufficiently supported by our data and have replaced it with *"lytic replication"* throughout the manuscript, including the manuscript title, to more accurately reflect our findings.

C. *There is no molecular confirmation of the status of infection beyond qPCR in any tissue. / Is there any active viral transcription or are infectious particles detected? The KSHV viruses with fluorescent reporters should be amenable to flow cytometry or immunohistochemistry. There are also sensitive methods to detect virus transcription that would support the interpretation of an active infection. Relying on viral DNA detection is not appropriate to indicate infection. This could be the detection of the initial inoculum into the blood, abortive infection events, or surveilling cells that took up the particles and carried to lymph nodes. / The LN sites are interesting but not validated beyond PCR.*

RESPONSE: We agree with the Reviewer that molecular evidence beyond qPCR strengthens the interpretation of tissue infection. Given that the tissue samples were collected more than 100 days post-KSHV challenge, we consider it unlikely that the detected viral DNA represents residual inoculum rather than established infection. Importantly, in response to the Reviewers' recommendations, we performed LANA-1 IHC experiments and now demonstrate the presence of LANA-1-expressing cells in spleen (new **S12 Fig**), providing independent evidence supporting KSHV infection in this model. We acknowledge that we were unable to perform additional molecular or histological analyses of the lymph nodes because of limited remaining tissue.

D. *To establish common marmosets as 'a promising model for KSHV pathogenesis studies,' a deeper knowledge of the phenotype of the infected cells and cells that respond to infection should be performed. / What cell types does the virus infect in each tissue?*

RESPONSE: We fully agree with the Reviewer that identifying the cellular targets of KSHV infection in different tissues will be important for further establishing the common marmoset as a model of KSHV pathogenesis. However, due to limited sample availability and because this objective was beyond the scope of the original study, we were not able to perform additional characterization of the infected cells or cellular responses to infection. We now acknowledge this limitation in the revised Discussion and highlight it as an important direction for future studies.

E. *Viremia, salivary load, and anti-KSHV VCA IgG seem highly variable within and between experiments. / The authors report that tacrolimus led to inconsistent results in the two animals for each experiment, but there is variability among animals in each major experiment using their antibodies.*

RESPONSE: We agree with the Reviewer's assessment that responses to infection were variable both within and between experiments. We recognize that inherent differences in experimental protocols may have contributed to this variability, and have now added a new paragraph in the revised Discussion to further address these challenges and other limitations of the study. However, we would also like to highlight that common marmosets represent an outbred population, and therefore their immune responses are expected to more closely resemble those observed in the human population, where substantial inter-individual variability is common (PMID: 27062052; PMID: 18515794), rather than the relatively uniform responses typically observed in inbred laboratory animal strains. Thus, while this biological variability presents challenges for model development, it also reflects an important feature of the natural host response that may enhance the translational relevance of this model.

F. *What are the measurable criteria for determining if an animal is infected and for scoring reactivation versus low-level persistent replication? What cohort sizes are needed to reach statistical power? What are the key timepoints and sites of infection?*

RESPONSE: We define animals as infected if KSHV DNA detection in at least ≥2 distinct tissues. Regarding the use of the term "reactivation," as described in our response to **Comment B**, we have revised the manuscript to refer to the observed increases in oral viral shedding and serum anti-VCA antibody levels as increased "replication" rather than "reactivation." Further studies will be required to determine whether these increases represent true reactivation from latency or an increase in ongoing, low-level persistent replication following immunosuppression. Regarding cohort sizes and statistical power, we agree that increasing the number of animals in each group would strengthen the analyses. However, due to the high costs associated with non-human primate studies and the limited availability of *Callitrichine gamma-herpesvirus 3*-negative marmosets, we were unable to increase the number of marmosets during the study. We thus acknowledge in the revised Discussion that additional studies will be necessary to further establish and validate this model. We apologize if we misunderstood the Reviewer's question regarding key timepoints and sites of infection; we refer the Reviewer to our Discussion (paragraph on Line 782), which summarizes the organs in which we detected KSHV most frequently.

G. *It is unclear what the cutoff is for seropositivity in the anti-KSHV VCA IgG. VCA antibodies are generated but it seems that seroconversion to a lytic antigen and detection of viral DNA is not 1:1, thus very little productive infection may occur in this model. Were other antibodies examined, such as those against LANA or neutralizing antibodies against glycoproteins?*

RESPONSE: We have defined the anti-KSHV VCA/LANA-1 IgG ELISA seropositivity cut-offs based on pre-challenge values and have now delineated this threshold on the revised figures to improve clarity and facilitate interpretation of results. We agree with the Reviewer that the relationship between VCA seroconversion and detection of viral DNA may not be one-to-one and that limited productive infection could represent a feature of this model. However, additional characterization with larger cohorts and longitudinal sampling at multiple timepoints will be required to confirm this possibility and determine the extent of viral replication in this system. This is particularly relevant given that viral shedding in humans can be highly variable (PMID: 39371366, PMID: 39363326) and that viral DNA detection does not always directly correlate with seropositivity (PMID: 8657239, PMID: 27062052). Regarding antibody responses, we initially focused on the lytic antigen VCA because of its established use as a serological marker for KSHV infection (PMID: 24675986). However, we agree that assessing antibodies targeting latent antigens is key for further characterization of the model, and have therefore now evaluated plasma anti-KSHV LANA-1 IgG responses in the marmosets. We detected LANA-1 seroconversion in two animals, and now report these findings in the revised manuscript (**Fig 1 and Fig 7**).

H. *The CD4 antibody data seems promising in the validation experiment but then there is an issue with long-term activity of the reagent in a replicate experiment. It seems they might remove the CD4 antibody from this report (or shift entirely to supplement) since that is a technical issue with loss of binding activity that is a bit confusing and detracts from the study*.

RESPONSE: We agree with the Reviewer that the loss of mAb stability represents an important technical limitation that prevented us from achieving our original experimental objectives. However, in order to remain transparent and allow us to still draw meaningful conclusions from these experiments, we maintained this data in the main text and focused the analysis on the effect of successful depletion of $CD8^+$ T cells, treating anti-CD4-treated groups as essentially non CD4-depleted and data derived from these animals as such. This approach allows us to accurately represent the experimental outcomes while ensuring responsible and ethical use of previously generated animal data.

I. *Infectious virus is not isolated from the saliva. / The salivary DNA is interpreted as a marker of reactivation and shedding. Are infectious particles isolated from this material? Salivary DNA was reported for only one timepoint. Were more samples collected to identify the duration of DNA shedding? Was it cyclical in nature, related to the degree of CD8 T cell depletion, or just observed at a single timepoint? How can the authors differentiate low-level replication after dissemination to the oral cavity/salivary glands from reactivation in a possible different cell type where bona fide latency was established.*

RESPONSE: We agree with the Reviewer that determining whether infectious virus is present in saliva and defining the kinetics of viral shedding are important aspects for further characterization of this model. However, because the original experiment was not designed to assess infectious virion production in the saliva, the saliva samples were not collected or processed in a way that would allow adequate virus preservation, and therefore the reliability and interpretation of subsequent infectivity assays would be limited. In addition, most of our saliva samples were depleted for DNA isolation and qPCR assays, preventing further evaluation using additional assays. Regarding the duration and dynamics of viral shedding, sampling frequency restrictions prevented us from collecting saliva samples at additional timepoints, hence we could not further assess the nature of the shedding events beyond the timepoints presented in the Results. As described in our response to **Comment B**, we now refer to the observed increase in oral viral shedding and serum anti-VCA levels as an increase in "replication" rather than "reactivation", as our current data cannot distinguish between a switch from latency to lytic reactivation versus an increase in ongoing low-level replication. Future studies with experimental designs specifically optimized to evaluate viral reservoirs, cell types infected, infectious virus production, and longitudinal shedding patterns will be required to address these important questions.

J. *In multiple figures, it is not clear why % of lymphocytes is used to monitor depletion when they show in Figure 4 that data is better presented as absolute # cells*.

RESPONSE: We agree with the Reviewer that absolute cell counts provide a more accurate measure of lymphocyte depletion than percentages. Unfortunately, absolute cell counts were only available for the experiment presented in **Fig. 4/S5 Fig**. As acknowledged in the manuscript, the remaining experiments could therefore only be analyzed and presented using the available percentage/frequency data.

K. *The authors directly measure anti-CD4 and anti-CD8 antibodies. The initiation and endpoints are useful to show baseline and turnover of antibodies, but there is only one timepoint where antibodies are detected in Fig 3D-E and Fig 6C. An analysis of the immunophenotype of CD4 and CD8 T cells at the time of virus challenge and at endpoint in non-depleted vs depleted groups would be informative.*

RESPONSE: We agree with the reviewer that additional antibody measurement timepoints would be ideal. In this case our assessment was limited due to the sampling restrictions in marmosets: owing to the small marmoset body size (350–450 g), blood collection is restricted to approximately 1% of body weight per month, which limits both the volume of blood that can be collected and the frequency of longitudinal sampling. Regarding immunophenotyping of CD4 and CD8 T cells, we note that while we indeed assessed the frequency of these cells at the challenge timepoint and study endpoint (**Fig 6**), we agree that further immunophenotyping of their respective sub-populations would provide greater insights; however, the limited amount cells obtained at each sampling timepoint as per the aforementioned limitations prevented us from performing further analyses. We discuss this sampling limitation in our revised Discussion.

L. *The authors criticize other models for using 'high' titer of virus inoculum, yet they are using a half million infectious units and a direct IV infection*.

RESPONSE: We appreciate the Reviewer's concern regarding the challenge dose and would like to clarify our comparison with previously established KSHV models. In humanized mice, previously reported KSHV challenge doses range from 2.5x10^5 to 5x10^6 IU (PMID: 24516154, PMID: 38844783, PMID: 41350288, PMID: 38767375). Indeed, our tacrolimus experiments used 1-2x10^7 IU challenge doses to replicate previous work by the Jae Jung group, which used 5x10^7 IU (PMID: 19798430). However, in our mAb depletion experiments we were able to further reduce the challenge dose to 5x10^5 IU, which is within the same log or lower than doses used in humanized mice. Given the markedly larger size of marmosets relative to mice, and the fact that the KSHV target cells in humanized mice represent the natural host cells of KSHV (human immune cells), our dosing in marmosets could be considered relatively lower. Nevertheless, we acknowledge that the challenge dose remains an important parameter requiring further optimization.

M. *Direct IV infection is not a natural route of infection, perhaps modeling transmission by IV drug use or organ transplantation. Given they use a non-physiological IV route- it is not surprising that the spleen and liver that effectively filter the blood are the organs with the highest and most consistent KSHV copy numbers*.

RESPONSE: We agree with the Reviewer that intravenous (IV) inoculation does not represent the predominant natural route of KSHV transmission and may more closely model scenarios involving blood exposure, such as transfusion, organ transplantation, or other parenteral exposures. However, one of the original goals of this work was to further confirm reproducibility of the IV challenge approach, as this represented the most commonly used and best-characterized route for KSHV challenge in common marmosets at the time this study was initiated. As we now address in our revised Discussion, future studies should evaluate more physiologically relevant routes of infection, including mucosal and oral transmission pathways, particularly given our observation of oral viral shedding in infected animals,.

N. *There is no direct comparison of tacrolimus to the CD8 depletion regimen, [thus], given the variability between animals and between experiments, it is difficult to determine which approach is better without a direct comparison of impacts on T cells and endpoints including impacts on viremia, viral load in tissues, and/or viral DNA in the saliva. Why not examine immune suppression prior to infection with the two regimens using a matched age cohort of animals (n=2 each) at the same institution with the same virus stock/inoculum and same endpoints? This would clarify if the CD8 T depletion is superior and would better establish the model.*

RESPONSE: Given that tacrolimus proved to be a nonviable immunosuppressive option due to its toxicity to the marmosets, and the difficulty of directly monitoring tacrolimus-mediated immunosuppression , our goal in developing the mAbs was to establish a safer, more targeted, and experimentally traceable alternative. Therefore, we did not perform a direct head-to-head comparation between the two immunosuppressive strategies. Importantly, previous work (PMID: 19798430, Pre-print https://doi.org/10.1101/2024.10.31.621273) and ours has demonstrated that immunosuppression is not a pre-requisite for infection, thus our mAbs rather become a tool to study infection in the immunosuppressive context. Indeed, further experiments are needed to fully standardize and characterize immunosuppressive regimens and their effect on infection both before and after challenge, but these objectives are beyond the scope of our study.

O. *Throughout the paper, line graphs are used to connect the initiation of an experiment, a single timepoint during the experiment, and then end point (Fig 3D-E). This is a bit misleading in terms of the kinetics of the readout such as viral DNA release, since there is an apparent increase or decrease with time that is influenced by the graph's scale and not reflecting collected data. Bar graphs would be more appropriate if more timepoints are not included or sampling is limited.*

RESPONSE: We acknowledge that true kinetics may not be fully represented in our data given our limited sampling timepoints; however, we note that it is standard to present longitudinal data in this way (i.e. connected with lines) despite the discrete nature of this data (PMID: 27570844). Indeed, the line itself does not necessarily represent real data between timepoints, but the general trajectory of the data change, and helps to easily identify variation patterns in a subject or group. Nevertheless, we agree that additional

sampling would provide a more accurate characterization of viral and immunological kinetics, and we now discuss this limitation in the revised Discussion.

P. *Viral load is typically log transformed*.

<u>RESPONSE:</u> While we acknowledge this is traditionally true, here we have opted to display non-transformed data to allow appreciation of small differences between samples.

Q. *Statistics were not applied to the final experiment with the larger 6 animal groups for figure 7 and 8 to compare depleted (d-12, d-21) and non-depleted animals, whether IgG or viral load across tissues. The authors make conclusions about the levels of VCA IgG indicating primary exposure (non-depleted) vs lytic reactivation (depleted) without statistical analysis of trends over time or comparisons at timepoint between non-depleted and depleted cohorts to support statistical significance. Correlations might be used to identify the impact of degree of peak depletion on VCA IgG or viral load at different sites to better support the premise that CD8T cell depletion is leading to reactivation.*

<u>RESPONSE:</u> We appreciate the Reviewer's suggestion and have performed additional statistical analyses where appropriate. For the anti-VCA IgG data in **Fig 7**, we performed a mixed-effects analysis with Geisser-Greenhouse correction to evaluate treatment effects over time. This analysis did not detect a significant overall treatment effect (p=0.0638). We then performed repeated measures one-way ANOVA with Geisser-Greenhouse correction followed by Dunnett's multiple comparison test to assess changes over time within each treatment group. This analysis identified significant increases in anti-VCA IgG levels between the pre-infection timepoint (Day -21) and two post-infection timepoints in the Anti-CD8 (initiated Day -12) and Mock Anti-CD4 groups. **Fig 7** and its legend have been updated accordingly.
For viral DNA in **Fig 8**, two-way ANOVA followed by Tukey's multiple comparisons test did not identify significant differences between treatment groups in non-lymphoid tissues. In the lymphoid tissues, multiple comparisons test revealed significant differences in the spleen between the Mock Anti-CD4 groups and all three other treatment groups. However, given the fact that the ANOVA test showed no overall difference driven by treatment (p=0.2701), and a very modest difference driven by the interaction between tissue and treatment (p=0.0415), these results should be interpreted with caution. The small sample size of the groups could have also contributed to these results; indeed, by visual inspection the Mock Anti-CD4 and PBS groups seem to follow a similar trend, but the difference in sample size (n=6 vs n=4) could have precluded an accurate comparison. Additionally, tissue sampling bias, as now further discussed in our revised Discussion, can result in false negatives. Hence, reporting these statistics might be misleading and we have thus not reported them in the main manuscript.

R. *The paper is well-written, but highly technical in nature and chunks of data (control Figs 2 and 4 for instance) could be moved to the supplemental data or published elsewhere to keep the focus on successful strategies.*

<u>RESPONSE:</u> We thank the Reviewer for their commendation. We acknowledge that the study is technically extensive and contains a substantial amount of data. However, we have presented the data in its current form to address concerns raised during the original and current review rounds and to provide a transparent account of both the successful strategies and the challenges encountered during development of this model. We believe that including these control experiments provides important context for interpreting the final experimental approaches and conclusions.

<u>Minor Issues: Editorial and Data Presentation Modifications</u>

S. *Line 117 Not sure it is appropriate to cite a preprint since that has not completed peer-review*.

<u>RESPONSE:</u> We thank the Reviewer for this comment. This preprint was included in response to a request from Reviewer 3 during the initial review round (Comment O), and we have clearly indicated it as a preprint in-text for full transparency.
