# Supplementary material for: Newly developed monoclonal antibodies for marmoset immunosuppression enable KSHV lytic replication and salivary shedding: A promising model for KSHV pathogenesis studies
Source: PLoS Pathog. 2026 Oct 26;22(10):e1014698. doi: 10.1371/journal.ppat.1014698 (PMC13722297; doi:10.1371/journal.ppat.1014698)
Supplement: Peer review history

# Peer review: Decision Letter 0

**DOI:** 10.1371/journal.ppat.1014698.r001

**Authors:** Donna Neumann, Benjamin Gewurz

26 Apr 2026

Newly developed monoclonal antibodies for marmoset immunosuppression enable KSHV lytic reactivation and salivary shedding: a promising model for KSHV pathogenesis studies.

PLOS Pathogens

Dear Dr. Ogembo,

Thank you for submitting your manuscript to PLOS Pathogens. After careful consideration, we feel that it has merit but does not fully meet PLOS Pathogens’s publication criteria as it currently stands. Therefore, we invite you to submit a revised version of the manuscript that addresses the points raised during the review process.

Please submit your revised manuscript by Jun 25 2026 11:59PM. If you will need more time than this to complete your revisions, please reply to this message or contact the journal office at plospathogens@plos.org. When you're ready to submit your revision, log on to https://www.editorialmanager.com/ppathogens/ and select the 'Submissions Needing Revision' folder to locate your manuscript file.

Please include the following items when submitting your revised manuscript:

* A letter that responds to each point raised by the editor and reviewer(s). You should upload this letter as a separate file labeled 'Response to Reviewers'. This file does not need to include responses to any formatting updates and technical items listed in the 'Journal Requirements' section below.

* A marked-up copy of your manuscript that highlights changes made to the original version. You should upload this as a separate file labeled 'Revised Manuscript with Track Changes'.

* An unmarked version of your revised paper without tracked changes. You should upload this as a separate file labeled 'Manuscript'.

If you would like to make changes to your financial disclosure, competing interests statement, or data availability statement, please make these updates within the submission form at the time of resubmission. Guidelines for resubmitting your figure files are available below the reviewer comments at the end of this letter.

We look forward to receiving your revised manuscript.

Kind regards,

Benjamin E Gewurz, M.D., Ph.D.

Academic Editor

PLOS Pathogens

Donna Neumann

Section Editor

PLOS Pathogens

Editor-in-Chief

PLOS Pathogens

PLOS Pathogens

orcid.org/0000-0002-7699-2064

**Additional Editor Comments:**

As you will see, the reviewers including one new reviewer (reviewer #4) appreciated the manuscript, but significant concerns were raised. Please respond carefully to the reviewer #3 and#4 critiques.

**Journal Requirements:**

1) We ask that a manuscript source file is provided at Revision. Please upload your manuscript file as a .doc, .docx, .rtf or .tex. If you are providing a .tex file, please upload it under the item type u2018LaTeX Source Fileu2019 and leave your .pdf version as the item type u2018Manuscriptu2019.

2) Your Response to Reviewers should be uploaded as a single, individual file. Please remove your Response to Reviewers from your Cover Letter.

3) We noticed that you used the phrase 'data not shown' in the manuscript. We do not allow these references, as the PLOS data access policy requires that all data be either published with the manuscript or made available in a publicly accessible database. Please amend the supplementary material to include the referenced data or remove the references.

4) We do not publish any copyright or trademark symbols that usually accompany proprietary names, eg ©,  ®, or TM  (e.g. next to drug or reagent names). Therefore please remove all instances of trademark/copyright symbols throughout the text, including:

- ® on pages: 34, and 41

- TM on pages: 39, and 41.

5) In the online submission form, you indicated that your data will be submitted to a repository upon acceptance. We strongly recommend all authors deposit their data before acceptance, as the process can be lengthy and hold up publication timelines. Please note that, though access restrictions are acceptable now, your entire minimal dataset will need to be made freely accessible if your manuscript is accepted for publication. This policy applies to all data except where public deposition would breach compliance with the protocol approved by your research ethics board. If you are unable to adhere to our open data policy, please kindly revise your statement to explain your reasoning and we will seek the editor's input on an exemption.

6) Please revise your current Competing Interest statement to the standard "The authors have declared that no competing interests exist."

7) Some material included in your submission may be copyrighted. According to PLOS’s copyright policy, authors who use figures or other material (e.g., graphics, clipart, maps) from another author or copyright holder must demonstrate or obtain permission to publish this material under the Creative Commons Attribution 4.0 International (CC BY 4.0) License used by PLOS journals. Please closely review the details of PLOS’s copyright requirements here: PLOS Licenses and Copyright. If you need to request permissions from a copyright holder, you may use PLOS's Copyright Content Permission form.

Please respond directly to this email and provide any known details concerning your material's license terms and permissions required for reuse, even if you have not yet obtained copyright permissions or are unsure of your material's copyright compatibility. Once you have responded and addressed all other outstanding technical requirements, you may resubmit your manuscript within Editorial Manager.

Potential Copyright Issues:

i) Figures S2A, and S9A. Please confirm (a) that you are the photographer; or (b) provide written permission from the photographer to publish the photo(s) under our CC BY 4.0 license.

ii) Figure 4A. Please confirm whether you drew the images / clip-art within the figure panels by hand. If you did not draw the images, please provide (a) a link to the source of the images or icons and their license / terms of use; or (b) written permission from the copyright holder to publish the images or icons under our CC BY 4.0 license. Alternatively, you may replace the images with open source alternatives. See these open source resources you may use to replace images / clip-art:

- https://commons.wikimedia.org

- https://openclipart.org/

iii) Figures 1A, 1F, 3A, 6A, and S6A. We note that they are created through BioRender. Please confirm that you hold a Premium account and provide a pdf copy of the CC BY 4.0 Licence as provided by BioRender. For instructions on how to generate a CC BY 4.0 license for your figure, please see the guidelines here: https://help.biorender.com/hc/en-gb/articles/21282341238045-Publishing-in-open-access-resources

Please ensure the figure legend contains appropriate attribution text & a link to biorender.com

If you are using the free assets from BioRender, we are unable to publish these images as they are licenced under a stricter licence than CC BY 4.0. In this case we ask you to remove the BioRender images and replace them with open source alternatives.

See these open source resources you may use to replace images / clip-art:

- https://bioart.niaid.nih.gov/

- https://bioicons.com/

- https://healthicons.org/

- https://scidraw.io/

- https://reactome.org/icon-lib

- https://www.phylopic.org/images

- https://journals.plos.org/plosbiology/article?id=10.1371/journal.pbio.3002395.

Note: If the reviewer comments include a recommendation to cite specific previously published works, please review and evaluate these publications to determine whether they are relevant and should be cited. There is no requirement to cite these works unless the editor has indicated otherwise.

**Reviewers' Comments:**

Reviewer's Responses to Questions

**Part I - Summary**

Please use this section to discuss strengths/weaknesses of study, novelty/significance, general execution and scholarship.

Reviewer #1: This manuscript presents a significant methodological advancement in KSHV pathogenesis research through the development and characterization of marmoset-chimerized monoclonal antibodies (TAWA and OCHO) for immunosuppression studies. The work addresses a critical gap in the field by establishing the first non-human primate model capable of demonstrating KSHV lytic reactivation and salivary shedding.

Strengths: The study's primary innovation lies in creating marmoset-specific anti-CD4 and anti-CD8 monoclonal antibodies that overcome previous species compatibility limitations. The authors successfully demonstrate effective immunosuppression comparable to that seen in human AIDS patients, making this an clinically relevant model. Most notably, they achieve the first documented KSHV salivary shedding in marmosets, representing a paradigm-shifting breakthrough that enables future transmission and intervention studies. The experimental design employs multiple complementary detection methods (qPCR, flow cytometry, immunofluorescence) and includes appropriate controls throughout. The statistical analysis is rigorous with adequate sample sizes and proper test selection.

Technical Execution:

The antibody development and characterization work is methodologically sound. Flow cytometry demonstrates specific binding to marmoset T-cell populations, and in vivo depletion studies show sustained immunosuppression over clinically relevant timeframes. The KSHV infection protocols successfully establish latent infection in appropriate cellular compartments, and the demonstration of salivary viral DNA during immunosuppression provides compelling evidence for lytic reactivation.

Significance and Impact:

This model fills a crucial void in KSHV research. Previous animal models lacked the ability to recapitulate key aspects of human KSHV pathogenesis, particularly viral reactivation and shedding. The successful demonstration of salivary shedding makes this model invaluable for studying KSHV biology and testing therapeutic interventions. The work has immediate implications for understanding KSHV-associated diseases in immunocompromised populations.

Major Limitations:

The most significant concern involves inconsistent viral quantification methodologies across experiments. The authors employ different indicator cell lines (iSLK vs HEK293) and varying viral inocula (1×10^7 to 2×10^7 IU), making direct comparisons problematic since infectious units are cell-line dependent. Additionally, the manuscript uses both absolute copy numbers and ΔCt values for viral load measurements, introducing potential bias in data interpretation. These methodological inconsistencies complicate the interpretation of infection outcomes and limit reproducibility assessments.

The study also encounters practical challenges including loss of anti-CD4 antibody functionality during experiments, necessitating post-hoc experimental restructuring. While the authors demonstrate that replacement batches restore binding activity, the in vivo validation of these reagents remains limited.

Overall Assessment:

Despite methodological concerns, this work represents a substantial contribution to KSHV research. The core innovation - developing functional marmoset-specific immunosuppressive antibodies - is technically sound and addresses a real need in the field. The achievement of viral reactivation and salivary shedding in a non-human primate model is unprecedented and scientifically significant. While the viral quantification issues require attention, they do not undermine the fundamental contributions of establishing this novel experimental system.

Reviewer #2: This paper characterizes the marmoset infection model for KSHV further. It also introduces two tools, antibodies for the depletion of CD4 and CD8 T cells in marmosets and it now demonstrates seroconversion and viral shedding. The current version has much improved over a previous version that was submitted to this journal. This reviewer's prior concerns have been addressed and the study is now suitable for publication.

Reviewer #3: This manuscript reported on a series of marmoset studies and demonstrated that the animals can be infected by KSHV upon intravenous challenge by the virus. Currently, there is a lack of good small animal models to study KSHV infection and disease pathogenesis, other than humanized mice which lack an intact immune system. The macaque is another potential animal model but it can only be infected by the simian homologue of KSHV. Thus, developing a susceptible non-human primate model, like the marmoset will be valuable to the KSHV research field. The authors have demonstrated that suppressing the cell mediated immune response of the animals can promote infection, and the team has developed two chimeric anti-marmoset T cells monoclonal antibodies, one against CD4 and the other against CD8, that can effectively suppressed the T cells responses and used them in marmoset to enhance KSHV infection, which is the major focus of the manuscript. These antibodies will be valuable reagents to be used in future studies.

The immune suppressed animals were shown to be infectable by KSHV, and viral DNA was detectable in the saliva DNA. Viral DNAs were also detected in virous tissues in the sacrificed animals. This study has supported an earlier study showing that marmoset can be susceptible to infection upon T cell suppression. However, even for this study efficient infection was only observed in suppressed animals and was hardly detectable in normal animals even upon intravenous injections with a very high dose of virus. Infection and response to the infection was also highly variable from one animal to animal, as shown in figure 5. Only one animal cj2254 showed a notable anti-VCA antibody response and the others did not, making the marmoset still not a reliable infection model for pre-clinical study. It is understandable that marmoset availability is limited and endangered, thus the authors have combined the results from different studies and protocols, from different batches of animals and from different primate centers, that could have contributed to the variability the studies. Another concern is the use of different strains of KSHV, different doses of viruses in different experiments, from 5x105 to 109, using different sources of KSHV, which can potentially create substantial variability in various aspects of the study. The manuscript was also very poorly put together, including data from experiments that were not optimized, such as showing cell percentages rather than absolute cell counts, using antibodies with stability not verified, all resulting in concerns with the presented results.

Reviewer #4: Animal models for KSHV chronic infection and disease are needed to investigate pathogenesis and therapeutics. Humanized mice and common marmosets are two models that support limited KSHV infection. Here the authors aim to improve upon the common marmoset infection model by increasing the efficacy of immune suppression that is expected to increase virus infection/replication/reactivation. They find tacrolimus administration by the intravenous or oral route months after KSHV infection leads to disparate patterns and aim to identify novel antibodies against CD4 and CD8 T cells that will lead to improved immune suppression. They validate two novel chimeric antibodies but upon administration in a challenge experiment they learn that the anti-CD4 antibody becomes inactive. They continue with the CD8 T cell depletions and report that CD8 T cell depletion is associated with detection of KSHV DNA in saliva, faster seroconversion and the detection of viral DNA in more lymph nodes compared to non-depleted animals. This is a potentially useful NHP model for KSHV but many important aspects of the systems are not reported. Major limitations are that there is no direct evidence that CD8 T cells responds to KSHV infection such as by immune phenotype, effector cytokine production or cell-killing and that there is no direct evidence that CD8 T cell depletion leads to more KSHV reactivation from latency. There is no molecular confirmation of the status of infection beyond qPCR in any tissue. Infectious virus is not isolated from the saliva. The common marmoset animal model has the potential to validate therapeutics using a 5 month endpoint, but in the absence of understanding the biological underpinnings of lymph node infection or salivary production as they relate to KSHV-specific T cell responses and given the high variability between animals, this model as it stands now is underpowered in providing new insight regarding pathogenesis or protective immune responses that control infection.

**********

**Part II – Major Issues: Key Experiments Required for Acceptance**

Please use this section to detail the key new experiments or modifications of existing experiments that should be absolutely required to validate study conclusions.

Generally, there should be no more than 3 such required experiments or major modifications for a "Major Revision" recommendation. If more than 3 experiments are necessary to validate the study conclusions, then you are encouraged to recommend "Reject".

Reviewer #1: No additional experiments are required for acceptance. The current data, particularly the demonstration of salivary viral shedding during immunosuppression, provides sufficient evidence for the authors' conclusions regarding KSHV reactivation in this model system. The methodological concerns identified can be adequately addressed through textual modifications rather than new experimental work.

The inconsistent viral quantification approaches, while important limitations, reflect the practical challenges of developing this novel model system. Requiring standardization experiments would impose an unreasonable burden given that the fundamental scientific conclusions are well-supported by the existing data.

The loss of anti-CD4 antibody functionality during the study, while concerning for future applications, does not invalidate the core findings since the authors demonstrate restoration of activity with replacement reagents and provide appropriate documentation of this issue.

Reviewer #2: (No Response)

Reviewer #3: The major points are:

1. It was previously shown by the team a number of years ago that marmoset can be infected by KSHV upon treatment with tacrolimus, and it is not clear on the rationale that this treatment was again used for thid study in the earlier experiment, which has now demonstrated that its use was inadequate due to its toxicity. This has distracted away from the major focus of this manuscript, which centers on the development and effectiveness of the chimeric antibodies in enhancing KSHV infection.

2. While higher copy of the viral DNA can be detected in tissues in suppressed animals, and viral DNA can be detected in the saliva DNA. However, it can only be detected upon immune suppression, and no viral DNA in the PBMC was detected. This does not reflect the natural history of infection in humans, where there were periodic viremia and viral shedding in saliva in asymptomatic KSHV infected individuals without immure suppression. Therefore, it is not clear that marmoset will be optimal small animal model to study KSHV disease pathogenesis.

3. Figure 3 showed substantial suppression of both CD4 and CD8 cells as early as 8 hours after treatment due to non-optimized analyses of the cells. This differed from data shown in figure 4 from other animals studied. It is unclear why the authors chose to present data that are not properly analyzed to begin with.

4. Infection in the tissues were all demonstrated by qPCR reflecting copies per microgram DNA, but tt is not clear these are truly from infected cells harboring intact latent viruses or they were from remnants of archived viral DNA. IHC detection of viral protein to demonstrate infected cells will be needed.

5. It is not clear why viral DNA can be detected in the saliva of suppressed animals but no viral DNA in the blood was detected at all studied time points, which is normally not the case in humans.

6. Similarly, the authors have suggested the viral reactivation in the infected tissues led to an increase in anti-KSHV IgG in the study animals. This will be surprising since no viral DNA was detected in the blood PBMC.

7. It is not clear why the authors have included the skin lesion in the supplement data, indicating that it is KS-like but with no detectable LANA, and presumably no detectable viral DNA This could simply be a skin disease upon prolonged immune suppression.

Other points that need to address:

8. Figure 5C showed the same symbols for all 5 animals studied. They need to be changed.

9. Introduction, line79. It is not true that KSHV infected individuals over 50 are at risk for endemic or classical KS. It is true only in certain populations in certain parts of the world.

10. The authors need to mention the rhesus macaque model. It has been extensively studied and had led to a better understanding of the KSHV pathogenesis even though this model was infected by the simian homologue of KSHV.

11. Line 308, CD4+/CD3- cells are not necessary accessory cells in the stromal in spleen, they could be monocytes/macrophages.

Reviewer #4: To establish common marmosets as ‘a promising model for KSHV pathogenesis studies,’ a deeper knowledge of the phenotype of the infected cells and cells that respond to infection should be performed. If these tools do not exist, it is a severe limitation of the model moving forward. Viremia, salivary load, and anti-KSHV VCA IgG seem highly variable within and between experiments. What are the measurable criteria for determining if an animal is infected and for scoring reactivation versus low-level persistent replication? What cohort sizes are needed to reach statistical power? What are the key timepoints and sites of infection?

What cell types does the virus infect in each tissue? Is there any active viral transcription or are infectious particles detected? The KSHV viruses with fluorescent reporters should be amenable to flow cytometry or immunohistochemistry. There are also sensitive methods to detect virus transcription that would support the interpretation of an active infection. Relying on viral DNA detection is not appropriate to indicate infection. This could be the detection of the initial inoculum into the blood, abortive infection events, or surveilling cells that took up the particles and carried to lymph nodes.

It is unclear what the cutoff is for seropositivity in the anti-KSHV VCA IgG. VCA antibodies are generated but it seems that seroconversion to a lytic antigen and detection of viral DNA is not 1:1, thus very little productive infection may occur in this model. Were other antibodies examined, such as those against LANA or neutralizing antibodies against glycoproteins?

Is a virus-specific T cell response generated in non-depleted animals? ELISPOTS can be used to profile IFNgamma or TNF production from splenocytes incubated with KSHV antigens. The CD4 antibody data seems promising in the validation experiment but then there is an issue with long-term activity of the reagent in a replicate experiment. It seems they might remove the CD4 antibody from this report (or shift entirely to supplement) since that is a technical issue with loss of binding activity that is a bit confusing and detracts from the study.

The salivary DNA is interpreted as a marker of reactivation and shedding. Are infectious particles isolated from this material? Salivary DNA was reported for only one timepoint. Were more samples collected to identify the duration of DNA shedding? Was it cyclical in nature, related to the degree of CD8 T cell depletion, or just observed at a single timepoint? How can the authors differentiate low-level replication after dissemination to the oral cavity/salivary glands from reactivation in a possible different cell type where bona fide latency was established.

In multiple figures, it is not clear why % of lymphocytes is used to monitor depletion when they show in Figure 4 that data is better presented as absolute # cells.

The authors directly measure anti-CD4 and anti-CD8 antibodies. The initiation and endpoints are useful to show baseline and turnover of antibodies, but there is only one timepoint where antibodies are detected in Fig 3D-E and Fig 6C. An analysis of the immunophenotype of CD4 and CD8 T cells at the time of virus challenge and at endpoint in non-depleted vs depleted groups would be informative.

The authors report that tacrolimus led to inconsistent results in the two animals for each experiment, but there is variability among animals in each major experiment using their antibodies. The authors criticize other models for using ‘high’ titer of virus inoculum, yet they are using a half million infectious units and a direct IV infection. Direct IV infection is not a natural route of infection, perhaps modeling transmission by IV drug use or organ transplantation. Given they use a non-physiological IV route- it is not surprising that the spleen and liver that effectively filter the blood are the organs with the highest and most consistent KSHV copy numbers. The LN sites are interesting but not validated beyond PCR.

There is no direct comparison of tacrolimus to the CD8 depletion regimen, whether to examine the depletion or impact on KSHV infection. Thus, given the variability between animals and between experiments, it is difficult to determine which approach is better without a direct comparison of impacts on T cells and endpoints including impacts on viremia, viral load in tissues, and/or viral DNA in the saliva. Why not examine immune suppression prior to infection with the two regimens using a matched age cohort of animals (n=2 each) at the same institution with the same virus stock/inoculum and same endpoints? This would clarify if the CD8 T depletion is superior and would better establish the model.

Throughout the paper, line graphs are used to connect the initiation of an experiment, a single timepoint during the experiment, and then end point (Fig 3D-E). This is a bit misleading in terms of the kinetics of the readout such as viral DNA release, since there is an apparent increase or decrease with time that is influenced by the graph’s scale and not reflecting collected data. Bar graphs would be more appropriate if more timepoints are not included or sampling is limited. Viral load is typically log transformed.

Statistics were not applied to the final experiment with the larger 6 animal groups for figure 7 and 8 to compare depleted (d-12, d-21) and non-depleted animals, whether IgG or viral load across tissues. The authors make conclusions about the levels of VCA IgG indicating primary exposure (non-depleted) vs lytic reactivation (depleted) without statistical analysis of trends over time or comparisons at timepoint between non-depleted and depleted cohorts to support statistical significance. Correlations might be used to identify the impact of degree of peak depletion on VCAIgG or viral load at different sites to better support the premise that CD8T cell depletion is leading to reactivation.

The paper is well-written, but highly technical in nature and chunks of data (control Figs 2 and 4 for instance) could be move to the supplemental data or published elsewhere to keep the focus on successful strategies.

**********

**Part III – Minor Issues: Editorial and Data Presentation Modifications**

Please use this section for editorial suggestions as well as relatively minor modifications of existing data that would enhance clarity.

Reviewer #1: 1. Viral Quantification Methodology Discussion: The authors should add a comprehensive discussion in the Methods section comparing the different viral quantification approaches used across experiments. Specifically, they should:

- Acknowledge that different indicator cell lines (iSLK vs HEK293) produce non-comparable infectious unit measurements

- Explain the rationale for using different viral inocula across experiments

- Discuss the limitations of mixing absolute copy numbers with ΔCt relative measurements

- Provide recommendations for standardized quantification methods in future marmoset KSHV studies based on their experience

2. Flow Cytometry Validation Concerns: Address the potential interference between therapeutic TAWA antibody and detection antibodies in flow cytometry assays. The observed signal reduction upon co-incubation with L200 suggests possible epitope competition that could affect CD4+ T cell quantification accuracy. The authors should discuss this limitation and its potential impact on depletion measurements.

3. Antibody Stability and Replacement Documentation: Provide more detailed discussion of the anti-CD4 antibody functionality loss and the validation of replacement batches. While the authors appropriately acknowledge this issue, they should expand on the implications for reproducibility and provide stronger justification for their conclusions about TAWA reliability.

4. Sampling Strategy Limitations: The authors mention potential false negatives in tissue assessments due to localized infection. They should briefly discuss strategies to mitigate this in future studies, such as sampling multiple sites within organs or using larger tissue sections.

5. Editorial Corrections:

- Line 464: Remove word duplication ("quantify" appears twice)

- Ensure consistent nomenclature for viral strains and antibody designations throughout

6. Enhanced Limitations Discussion: Expand the Discussion section to more thoroughly address the methodological inconsistencies and their potential impact on data interpretation, while maintaining appropriate context about the pioneering nature of this work. These modifications will significantly strengthen the manuscript's methodological transparency and provide valuable guidance for future studies utilizing this novel model system.

Reviewer #2: (No Response)

Reviewer #3: (No Response)

Reviewer #4: Line 117 Not sure it is appropriate to cite a preprint since that has not completed peer-review.

**********

PLOS authors have the option to publish the peer review history of their article (what does this mean?). If published, this will include your full peer review and any attached files.

If you choose “no”, your identity will remain anonymous but your review may still be made public.

**Do you want your identity to be public for this peer review?**  For information about this choice, including consent withdrawal, please see our Privacy Policy.

Reviewer #1: **Yes:**  Brent A. Stanfield, Ph.D.

Reviewer #2: No

Reviewer #3: No

Reviewer #4: No

[NOTE: If reviewer comments were submitted as an attachment file, they will be attached to this email and accessible via the submission site. Please log into your account, locate the manuscript record, and check for the action link "View Attachments". If this link does not appear, there are no attachment files.]

**Figure resubmission:**

**Reproducibility:**

# Author comment: Author response to Decision Letter 1

**DOI:** 10.1371/journal.ppat.1014698.r002

26 Aug 2026

Attachment Submitted filename: PPATHOGENS-D-26-00589R1_Rebuttal.pdf

# Peer review: Decision Letter 1

**DOI:** 10.1371/journal.ppat.1014698.r003

**Authors:** Donna Neumann

3 Oct 2026

Dear Dr. Ogembo,

We are pleased to inform that your manuscript, "Newly developed monoclonal antibodies for marmoset immunosuppression enable KSHV lytic replication and salivary shedding: a promising model for KSHV pathogenesis studies.", has been editorially accepted for publication at PLOS Pathogens.

Before your manuscript can be formally accepted and sent to production, you will need to complete our formatting changes, which you will receive by email within a week. Please note that your manuscript will not be scheduled for publication until you have made the required changes.

IMPORTANT NOTES

(1) Copyediting and Proofreading: The corresponding author will receive a typeset proof for review, to ensure errors have not been introduced during production. Please review the PDF proof of your manuscript carefully, as this is the last chance to correct any errors. Please note that major changes, or those which affect the scientific understanding of the work, will likely cause delays to the publication date of your manuscript.

(2) Appropriate Figure Files: Please remove all name and figure # text from your figure files. Please also take this time to check that your figures are of high resolution, which will improve the readbility of your figures and help expedite your manuscript's publication. Please note that figures must have been originally created at 300dpi or higher. Do not manually increase the resolution of your files. For instructions on how to properly obtain high quality images, please review our Figure Guidelines, with examples at: http://journals.plos.org/plospathogens/s/figures.

We encourage you to use PACE (http://pace.apexcovantage.com/), a figure-checking tool which helps authors ensure all figures meet PLOS requirements so that figures can publish in the highest quality. PACE is a digital diagnostic and conversion tool for figure files. It will provide information about any failed check(s) and, if able, will automatically convert the figure into an acceptable file that passes quality checks.

To use PACE, you must first register as a user. Then, login and navigate to the UPLOAD tab, where you will find detailed instructions on how to use the tool.

(3) Striking Image: Please upload a striking still image to accompany your article if one is available (you can include a new image or an existing one from within your manuscript). Should your paper be accepted, this image will be considered for our monthly issue image and may also appear on our website to feature your article. Please upload this as a separate file, selecting "striking image" as the file type upon upload. Please also include a separate "Other" file with a caption, including credits and any potential copyright information. Please do not include the caption in the main article file. If your image is from someone other than yourself, please ensure that the artist has read and agreed to the terms and conditions of the Creative Commons Attribution License at http://journals.plos.org/plospathogens/s/content-license. Please note that PLOS cannot publish copyrighted images.

(4) Press Release or Related Media: If you or your institution will be preparing press materials for this manuscript, please inform our press team in advance at plospathogens@plos.org as soon as possible. We ask that you contact us within one week to plan ahead of our fast Production schedule. If you need to know your paper's publication date for related media purposes, you must coordinate with our press team, and your manuscript will remain under a strict press embargo until the publication date and time.

(5) Update your Profile Information: Now that your manuscript has been provisionally accepted, please log into Editorial Manager and update your profile, if needed. Go to http://www.editorialmanager.com/ppathogens, log in, and click on the "Update My Information" link at the top of the page. Please update your user information to ensure an efficient production and billing process.

(6) LaTeX users only: Our staff will ask you to upload a TEX file in addition to the PDF before the paper can be sent to typesetting, so please carefully review our Latex Guidelines http://journals.plos.org/plospathogens/s/latex in the meantime.

Thank you again for supporting Open Access publishing; we are looking forward to publishing your work in PLOS Pathogens.

Best regards,

Donna M Neumann, PhD

Section Editor

PLOS Pathogens

Donna Neumann

Section Editor

PLOS Pathogens

Dominique Soldati-Favre

Editor-in-Chief

PLOS Pathogens

***********************************************************

Reviewer's Responses to Questions

**Part I - Summary**

Please use this section to discuss strengths/weaknesses of study, novelty/significance, general execution and scholarship.<br/><br/>

Reviewer #1: This manuscript presents a significant methodological advancement in KSHV pathogenesis research through the development and characterization of marmoset-chimerized monoclonal antibodies (TAWA and OCHO) for immunosuppression studies. The work addresses a critical gap in the field by establishing the first non-human primate model capable of demonstrating KSHV lytic reactivation and salivary shedding.

Reviewer #4: I appreciate the importance of advancing in vivo models of KSHV. I am skeptical that KSHV infection of common marmosets as presented in this paper will be a rigorous pathogenesis model to explore treatments, but this paper does advance model development with the addition of an immunosuppressive monoclonal antibody to deplete CD8 T cells. The welcome addition of LANA protein detection by IHC supports an active infection program. The numerous text revisions that relay the data with less bias and point out the limitations are appropriate. I am largely satisfied with the response to reviewers and the revisions made. This data should be communicated to the field to spur further validation that may provide a system to test interventions.

--------------------

**Part II – Major Issues: Key Experiments Required for Acceptance**

Please use this section to detail the key new experiments or modifications of existing experiments that should be absolutely required to validate study conclusions.<br/><br/>Generally, there should be no more than 3 such required experiments or major modifications for a "Major Revision" recommendation. If more than 3 experiments are necessary to validate the study conclusions, then you are encouraged to recommend "Reject".<br/><br/>

Reviewer #1: NA

Reviewer #4: (No Response)

--------------------

**Part III – Minor Issues: Editorial and Data Presentation Modifications**

Please use this section for editorial suggestions as well as relatively minor modifications of existing data that would enhance clarity.<br/><br/>

Reviewer #1: NA

Reviewer #4: (No Response)

--------------------

PLOS authors have the option to publish the peer review history of their article (what does this mean?). If published, this will include your full peer review and any attached files.

If you choose “no”, your identity will remain anonymous but your review may still be made public.

**Do you want your identity to be public for this peer review?**  For information about this choice, including consent withdrawal, please see our Privacy Policy.

Reviewer #1: No

Reviewer #4: No

# Editor report: Acceptance letter

**DOI:** 10.1371/journal.ppat.1014698.r004

**Authors:** Donna Neumann

Dear Dr. Ogembo,

We are delighted to inform you that your manuscript, "Newly developed monoclonal antibodies for marmoset immunosuppression enable KSHV lytic replication and salivary shedding: a promising model for KSHV pathogenesis studies.," has been formally accepted for publication in PLOS Pathogens.

We have now passed your article onto the PLOS Production Department who will complete the rest of the pre-publication process. All authors will receive a confirmation email upon publication.

Thank you again for supporting open-access publishing; we are looking forward to publishing your work in PLOS Pathogens.

Best regards,

Dominique Soldati-Favre

Editor-in-Chief

PLOS Pathogens
